# Glioblastoma epigenome profiling identifies SOX10 as a master regulator of molecular tumour subtype

**DOI:** 10.1038/s41467-020-20225-w

**Published:** 2020-12-18

**Authors:** Yonghe Wu, Michael Fletcher, Zuguang Gu, Qi Wang, Barbara Costa, Anna Bertoni, Ka-Hou Man, Magdalena Schlotter, Jörg Felsberg, Jasmin Mangei, Martje Barbus, Ann-Christin Gaupel, Wei Wang, Tobias Weiss, Roland Eils, Michael Weller, Haikun Liu, Guido Reifenberger, Andrey Korshunov, Peter Angel, Peter Lichter, Carl Herrmann, Bernhard Radlwimmer

**Affiliations:** 1 Division of Molecular Genetics, German Cancer Research Center (DKFZ), Im Neuenheimer Feld 280, 69120 Heidelberg, Germany; 2 Heidelberg Center for Personalized Oncology (DKFZ-HIPO), Im Neuenheimer Feld 280, 69120 Heidelberg, Germany; 3 Division of Theoretical Bioinformatics, German Cancer Research Center (DKFZ), Im Neuenheimer Feld 280, 69120 Heidelberg, Germany; 4 Division of Signal Transduction and Growth Control, DKFZ/ZMBH Alliance, Im Neuenheimer Feld 280, 69120 Heidelberg, Germany; 5 Medical Faculty, Institute of Neuropathology, Heinrich Heine University, Moorenstr. 5, 40225 Düsseldorf, Germany; 6 German Cancer Consortium (DKTK), Partner site Essen/Düsseldorf, German Cancer Research Center (DKFZ), Im Neuenheimer Feld 280, 69120 Heidelberg, Germany; 7 Department of Neurology and Brain Tumor Center, University Hospital Zurich, Frauenklinikstrasse 26, CH-8091 Zurich, Switzerland; 8 Division of Molecular Neurogenetics, DKFZ-ZMBH Alliance, Im Neuenheimer Feld 280, 69120 Heidelberg, Germany; 9 Department of Neuropathology, University of Heidelberg, Im Neuenheimer Feld 220, 69120 Heidelberg, Germany; 10 Clinical Cooperation Unit, Neuropathology, German Cancer Research Center (DKFZ), Im Neuenheimer Feld 220-221, 69120 Heidelberg, Germany; 11 Health Data Science Unit, Medical Faculty Heidelberg, Im Neuenheimer Feld 267, 69120 Heidelberg, Germany; 12 Division of Molecular Genetics, German Cancer Research Center (DKFZ), Im Neuenheimer Feld 280, 69120 Heidelberg, Germany

**Keywords:** Cancer genomics, CNS cancer

## Abstract

Glioblastoma frequently exhibits therapy-associated subtype transitions to mesenchymal phenotypes with adverse prognosis. Here, we perform multi-omic profiling of 60 glioblastoma primary tumours and use orthogonal analysis of chromatin and RNA-derived gene regulatory networks to identify 38 subtype master regulators, whose cell population-specific activities we further map in published single-cell RNA sequencing data. These analyses identify the oligodendrocyte precursor marker and chromatin modifier SOX10 as a master regulator in RTK I-subtype tumours. In vitro functional studies demonstrate that *SOX10* loss causes a subtype switch analogous to the proneural–mesenchymal transition observed in patients at the transcriptomic, epigenetic and phenotypic levels. SOX10 repression in an in vivo syngeneic graft glioblastoma mouse model results in increased tumour invasion, immune cell infiltration and significantly reduced survival, reminiscent of progressive human glioblastoma. These results identify SOX10 as a bona fide master regulator of the RTK I subtype, with both tumour cell-intrinsic and microenvironmental effects.

## Abstract (web summary)

Glioblastoma is divided into four subtypes based on molecular profiling at the methylome and transcriptome level. Here the authors perform an integrative analysis of these subtypes resulting in the identification of SOX10 whose loss induces a mesenchymal phenotype and promotes tumour progression.

## Introduction

Glioblastoma is a highly malignant brain cancer with a particularly poor prognosis despite aggressive treatment comprising surgical resection and radiochemotherapy with temozolomide^1^. Recent large-scale genomics studies^2–5^ have identified four mRNA expression/DNA-methylation subtypes of glioblastoma and their hallmark genetic lesions: (1) IDH, characterised by glioma CpG island methylation phenotype (G-CIMP) hypermethylation due to mutations in the isocitrate dehydrogenase (*IDH*) *1* or *2* genes^3,6,7^; (2) MES (mesenchymal), associated with NF1 aberrations and increased tumour infiltration by tumour-associated macrophages/microglia^5^; (3) RTK I (receptor tyrosine kinase I), in which tumours commonly have *PDGFRA* gene amplifications; and (4) RTK II, exhibiting the classical *EGFR* gene amplification^8^. The MES, RTK I and RTK II subtypes correspond to the mesenchymal, proneural and classical RNA expression subtypes^4^, which were recently refined based on the analysis of exclusively IDH wildtype glioblastoma^5^. Transitions between these subtypes have been observed during the treatment of patients^9,10^ and may lead to worse prognosis^5,9^. It remains unclear whether these transitions are due to tumour cell plasticity or expansion of pre-existing resistant subpopulations.

Cancer Master Regulators (MRs) are proteins that define and regulate tumour cellular states^11^. It has been proposed that the systematic identification and characterisation of cancer MRs will provide a better understanding of basic cancer biology and potential therapeutic vulnerabilities^12^. While recent large-scale studies of transcriptomes and promoter-biased epigenomes have provided valuable insights into glioblastoma heterogeneity and cellular state transitions^2,5,9,13–18^, a comprehensive, genome-wide survey of the epigenetic landscape of primary glioblastoma subtypes using multidimensional data from the same patient tumour samples is, as yet, not available. Consequently, glioblastoma subtype MRs and their interactions with and effects on GB epigenetics remain largely unknown.

Here, we present an integrated epigenetic analysis of the four subtypes of adult glioblastoma. We performed methylome, transcriptome and epigenome profiling on a cohort of 60 untreated patient tumours and show that enhancers vary across subtypes. We identified 10 consensus subtype MRs based on our analysis of these matched tumour epigenetic and gene expression data. Repression of the RTK I MR SOX10 in human glioblastoma cell lines caused a subtype transition to a mesenchymal cellular state via the remodelling of active enhancers. We further show, using a recently described immunocompetent syngeneic mouse model that SOX10 loss leads to a dramatic decrease in survival, increased tumour invasion and immune cell infiltration. These results show that GB subtype transitions can have striking effects on clinically relevant tumour phenotypes and, as such, require further investigation.

## Results

### Primary glioblastoma epigenome profiling

**Fig. 1:**
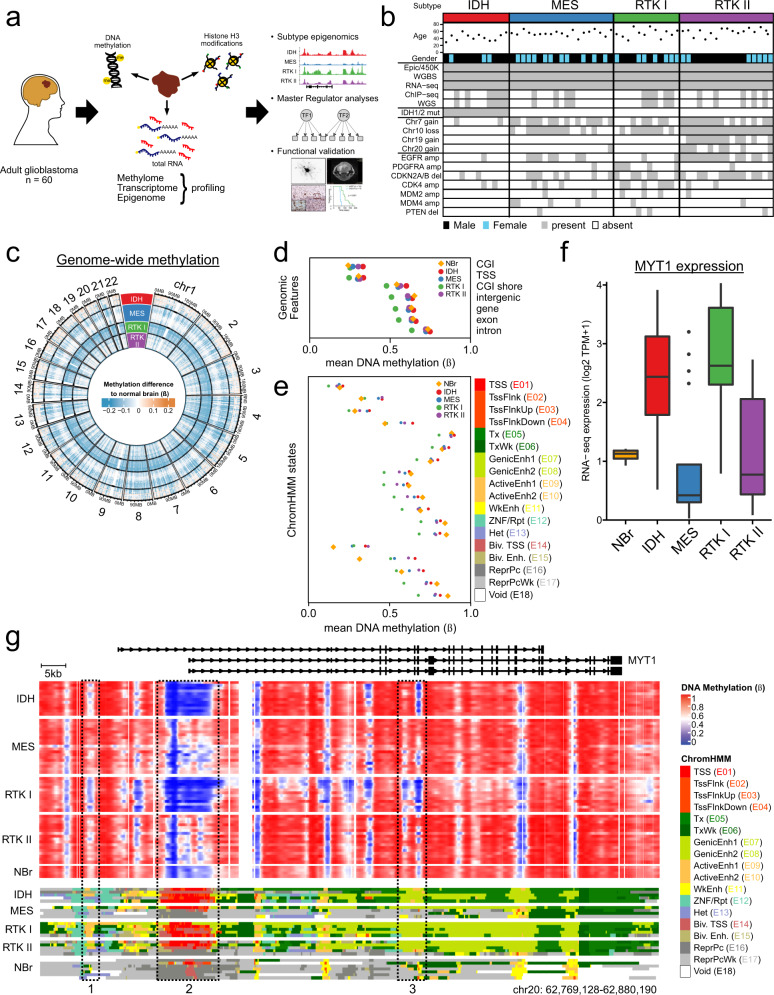
A comprehensive dataset of glioblastoma subtype epigenomics. **a** Study design. Based on extensive molecular profiling of 60 glioblastoma tumours, epigenetic glioblastoma subtypes were characterised, subtype Master Regulators were derived based on epigenome and transcriptome data, and functionally validated in glioblastoma cell lines and a syngeneic mouse model. **b** Characteristics of the 60 glioblastomas used in this study, including age, gender, methylation subtype, *IDH1* and *IDH2* mutation status and copy number aberrations. **c** Genome-wide differences in DNA methylation between glioblastoma subtypes and control brain tissue. Mean methylation (WGBS beta-values) for 100 kbp genome bins were determined for each subtype, and the difference to the control brain average. **d** Mean subtype DNA methylation (WGBS beta) at genomic features. **e** Mean subtype DNA methylation (WGBS beta) for each state of the 18-state ChromHMM model. **f** MYT1 RNA-seq expression (log_2_ TPM + 1) in subtypes, visualised as Tukey boxplots. Boxes correspond to the 25th, 50th/median and 75th percentiles; whiskers denote 1.5× the IQR from the median. Points mark outliers beyond 1.5× IQR. NBr, *n* = 4; IDH, *n* = 12; MES, *n* = 19; RTK I, *n* = 12; RTK II, *n* = 17 samples. **g** Integrative view of the glioblastoma epigenomic landscape. For *MYT1*, an oligodendrocyte marker gene, per-subtype methylation (top, beta-values) and ChromHMM annotation (bottom) are displayed. Control non-neoplastic, control brain ChromHMM annotations from the Roadmap Epigenome are included as a reference. DNA hypomethylation (top) in the IDH and RTK I subtypes correlates with active TSS (E01–04, box 2) and enhancer (E07–E011, boxes 1 and 3) ChromHMM states (bottom).

We selected 60 adult glioblastoma primary tumours and 4 normal brain samples (Supplementary Data 1, 2) for DNA methylome (methylation microarrays and whole genome bisulphite sequencing (WGBS)) and transcriptome (strand-specific, rRNA-depleted, total RNA sequencing (ssRNA-seq); mean 2 × 10^8^ reads) profiling (see Supplementary Data 3–5 for quality control data). The key resources used in this study are listed in Supplementary Table 1. Tumours were subtyped using a methylation microarray classifier (Supplementary Fig. 1a and Supplementary Data 6)^3^; the four subtypes (IDH: 12; MES: 19; RTK I: 12; RTK II: 17) and both major genotypes, IDH wildtype, present in about 90% of primary tumours (MES, RTK I, RTK II; 48), and IDH mutated (IDH; 12) were well represented. Subtyping based on WGBS data could clearly identify the IDH and RTK II groups, while MES and RTK I were less distinct. Consistent with this, methylation at gene-based features was generally variable across subtypes; however, the MES and RTK I subgroups could not be differentiated based on TSS or CGI methylation (Supplementary Fig. 1b, c) suggesting that these regions feature comparable methylation, and non-CGI and intergenic regions appear to be similar in the MES and RTK II subtypes (Supplementary Fig. 1c). For a subset of 20 tumours, we also profiled the H3K27ac, H3K4me1, H3K4me3, H3K36me3, H3K27me3 and H3K9me3 histone modifications by chromatin immunoprecipitation and sequencing (ChIP-seq) (Fig. 1a). The mutation status of *IDH1* and *IDH2* was determined by pyrosequencing. All IDH samples had *IDH1* R132H mutations and G-CIMP^6^ (Supplementary Fig. 1), while remaining tumours were IDH wildtype (IDH^wt^). These CIMP-, IDH^wt^ subtypes (MES, RTK I and RTK II) exhibited the classic glioblastoma copy number alterations (CNAs) consisting of gain of chromosome 7, loss of chromosome 10 and focal *CDKN2A/B* deletion. Amplifications of *EGFR* and gain of chromosomes 19 and 20 were strongly prevalent in RTK II, and *PDGFRA*, *CDK4* and *MDM2 or MDM4* amplifications were more frequent in RTK I tumours (Fig. 1b).

We next examined methylation differences between glioblastoma subtypes and normal brain. Large regions of the genome were hypomethylated in tumours relative to control, non-neoplastic normal brain tissue (Fig. 1c). Relative to the other tumour subtypes, RTK I tumours showed global hypomethylation. Similarly, IDH tumours were globally hypermethylated, showing that the G-CIMP phenotype extends beyond CpG islands and manifests across all genomic features. Tumour hypomethylation relative to normal tissue was most pronounced in intergenic regions, while CpG islands were relatively hypermethylated (Fig. 1d). Overall, these results agree with the current understanding of methylation changes in cancers.

We then used the 18-state Roadmap Epigenome ChromHMM model^19^ with our tumour histone mark ChIP-seq data to annotate each sample’s genome. We defined consensus subtype ChromHMM states and calculated their mean subtype methylation (Fig. 1e). We found that active TSS states (E01–E04) have relatively low methylation, while transcription (E05-E06), repressive (E12–13, E16–E17) and non-functional (E18) states have relatively higher methylation. Generally, the IDH subtype is the most hypermethylated, while RTK I is the most hypomethylated. Interestingly, the bivalent TSS and enhancer states (E14–E15) showed the broadest ranges, with a striking degree of tumour-specific hypermethylation, suggesting that differences in the methylation of tumour subtype and normal tissue may be more prevalent in genomic loci of defined function. These effects are frequently subtype-specific, as illustrated by myelin transcription factor 1 (MYT1), a regulator of oligodendrocyte differentiation. MYT1 is overexpressed in IDH and RTK I glioblastoma relative to normal brain, and shows corresponding hypomethylation and active chromatin states in the gene promoter and in known enhancer regions (Fig. 1f, g).

### Active enhancers are highly variable across glioblastoma subtypes

**Fig. 2:**
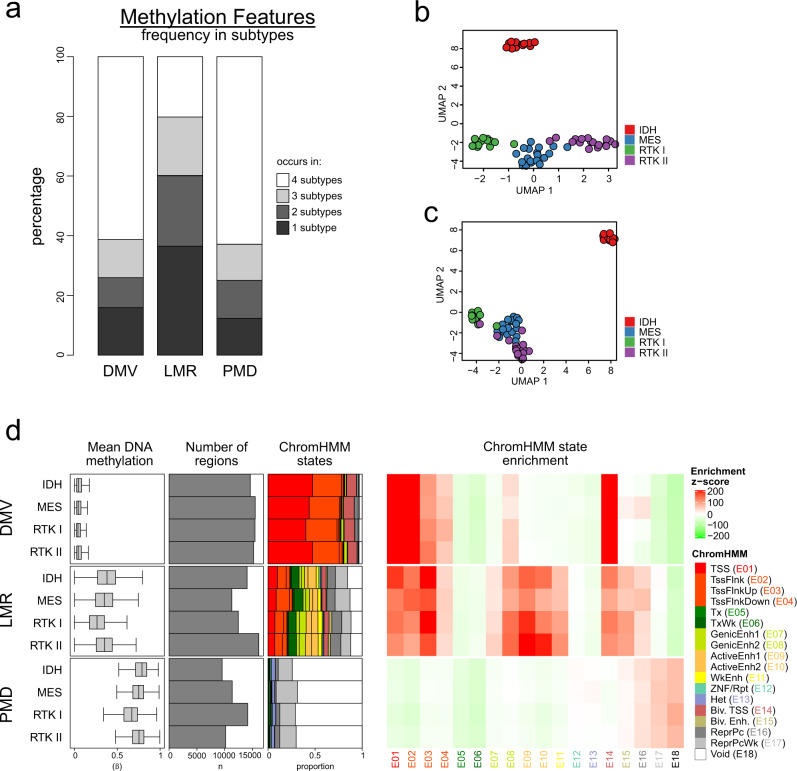
Active enhancer-LMR regions show highly variable methylation across glioblastoma subtypes. **a** Barplots showing the extent of subtype sharing of DNA methylation valleys (DMVs, left), lowly methylated regions (LMRs, middle) and partially methylated domains (PMDs, right). **b** Uniform manifold approximation and projection (UMAP) plot of the study glioblastoma samples based on the analysis of DNA methylation in LMRs. **c** UMAP plot of the glioblastoma samples based on the analysis of DNA methylation at enhancers. **d** Summary statistics for subtype DMVs, LMRs and PMDs, showing (left-right): mean DNA methylation (WGBS beta; boxes correspond to the 25th, 50th/median and 75th percentiles; whiskers denote 1.5× the IQR from the median); number of regions; ChromHMM state annotation; and ChromHMM state enrichment (see Methods for details of enrichment statistic calculation) for each subtype’s feature set. IDH, *n* = 12; MES, *n* = 19; RTK I, *n* = 12; RTK II, *n* = 17 samples.

We next took advantage of our unbiased WGBS data to call methylation features, i.e. differential methylation valleys (DMVs), partially methylated domains (PMDs) and lowly methylated regions (LMRs), which are enriched in promoter, heterochromatin and enhancer states, respectively^20–22^. We found that more than 60% of PMDs and DMVs were shared across all subtypes, and fewer than 17% were present in only one subtype each. Conversely, 37% of LMRs were specific for one subtype, suggesting that variable DNA methylation at LMRs is a substantial contributor to differences between subtypes (Fig. 2a). Consistent with this hypothesis, Uniform Manifold Approximation and Projection (UMAP) of LMR WGBS data clearly separated the subtypes, no matter whether all samples or only IDH^wt^ samples were analysed (Fig. 2b, c).

To learn more about the function of PMDs, LMRs and DMVs in glioblastoma, we used our ChromHMM model to annotate these features for each subtype (Fig. 2d). As expected, DMVs were strongly enriched in TSS states (E01–E03, E14) and PMDs contained mostly quiescent and repressive states (E16–E18). Chromatin states found in LMRs were more diverse, with the largest proportion (23%) being enhancer states (E07–E11 and E15). In the LMRs that are found only in one subtype, the enrichment of enhancer states was even more pronounced with 36%, indicating the importance of enhancers for defining subtype identities.

Focusing on active enhancers (E9–E10), we noted that 64% of tumour active-enhancer regions were unique to tumours and not shared in normal brain^19^. Of these, 59% tumour-specific active enhancers were unique to a single subtype, while only about 6% were shared by all subtypes. These results suggest that GB subtypes and their differing gene expression programmes are, at least in part, the result of subtype-specific enhancer activity.

### Core regulatory circuitry analysis identifies subtype Master Regulators

**Fig. 3:**
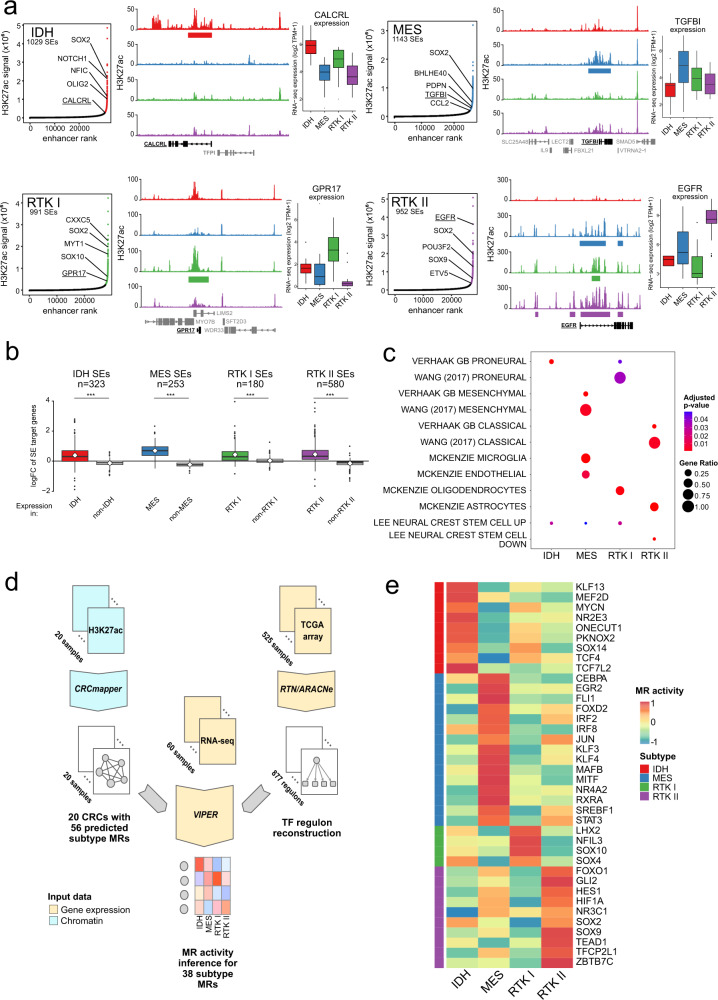
Core regulatory circuitry analysis identifies primary glioblastoma subtype Master Regulators. **a** Superenhancer (SE) identification using glioblastoma subtypes’ H3K27ac profiles. Left: Hockey stick plots showing enhancers (*x*-axis) ranked by their H3K27ac intensity (SES-normalised values, *y*-axis) are shown for the four subtypes. Selected SEs are labelled with their target genes. Centre: Exemplary subtype SEs, with mean subtype H3K27ac profiles for *CALCRL* (IDH), *TGFBI* (MES), *GPR17* (RTK I) and *EGFR* (RTK II). Subtype SEs are depicted as coloured bars below each H3K27ac profile. Right: RNA-seq gene expression (log_2_ TPM + 1) for the indicated genes, by subtype, visualised as Tukey boxplots. IDH, *n* = 12; MES, *n* = 19; RTK I, *n* = 12; RTK II, *n* = 17 samples. Boxes correspond to the 25th, 50th/median and 75th percentiles; whiskers denote 1.5× the IQR from the median. Points mark outliers beyond 1.5× IQR. **b** Tukey boxplots showing the gene expression log FC (limma) for target genes of each subtype SEs (defined by ANOVA on H3K27ac signal, minimum log fold change 1, Benjamini–Hochberg adjusted *P*-value = 0.1), comparing expression in that subtype to the average of the other 3. Mean log FC is indicated by the white diamond; *n* indicates the number of target genes; Boxes correspond to the 25th, 50th/median and 75th percentiles; whiskers denote 1.5× the IQR from the median. Points mark outliers beyond 1.5× IQR. ***Two-tailed *t*-test, *P*-value <2.2 × 10^−16^. **c** Selected gene signature enrichment results for the target genes of each subtype’s SEs. The size of each circle corresponds to the ratio of SE target genes in that gene signature, while the colour represents the adjusted *P*-value. **d** Overview of subtype Master Regulator (MR) identification. Firstly, 56 MRs were predicted with CRCmapper on the tumour H3K27ac profiles (*n* = 20). We extended this MR activity inference to the full tumour cohort (*n* = 60 samples) using VIPER to predict these 56 MRs’ activity within a gene regulatory network inferred in the TCGA cohort (*n* = 525 samples). In total, 38 subtype MRs were identified. **e** Heatmap showing the mean subtype activity for each MR (*n* = 38).

Enhancer activity is mediated by transcription factor proteins, including MRs. We therefore set out to identify the subtype MRs that are active in this heterogeneous enhancer landscape. Superenhancers (SEs) are a class of genomic loci that regulate cell identity genes, including MRs^23^. We performed subtype SE calling on our H3K27ac glioblastoma profiles (Fig. 3a). Some SEs exhibited subtype-specific enrichment; for example, RTK II tumours have an intronic SE in *EGFR* that is associated with higher H3K27ac signal and EGFR expression in this subtype. SEs with higher subtype H3K27ac signal correlated with target gene up-regulation in that subtype, suggesting that SEs regulate genes that are important for subtype identity in glioblastoma (Fig. 3b). This interpretation receives additional support from our observation that subtype LMRs are present in 65% of subtype SEs, on average.

We annotated each subtype’s SE’s target genes with mSigDB genesets and found significant enrichment for known glioblastoma subtype signatures^4,5^ (Fig. 3c). Furthermore, genes upregulated in neural crest stem cells were enriched in IDH, MES and RTK I, while these genes were mostly downregulated in RTK II. Oligodendrocyte markers were enriched in RTK I, while astrocyte markers were enriched in RTK II, agreeing with recent reports that glioblastomas co-opt SE landscapes used in normal CNS development^15^.

Core regulatory circuit (CRC) analysis identified a set of 56 candidate MRs across the four subtypes. We extended this analysis to the full cohort using a gene expression-based approach (Fig. 3d). To do so, we inferred a glioblastoma gene regulatory network using the TCGA gene expression microarray cohort (*n* = 525)^2^. We then used VIPER analysis^24^ to infer the activity of the 38 candidate CRC MRs that appear in this network. MRs were assigned to a subtype based on the average maximum VIPER NES (IDH: 9; MES: 16; RTK I: 4; RTK II: 9; Fig. 3e and Supplementary Data 7), including the previously reported MES MRs *CEBPA* and *STAT3*^13^.

### Master Regulator activity in cell types

Glioblastoma consist of mixtures of cells of different tumour subtypes, as well as normal cell populations such as tumour-associated macrophages, which are especially prevalent in MES^5,16,18,25,26^. We addressed this potential confounding of MR predictions made using our bulk tissue data, by extending our analysis to published single-cell RNA-seq profiles of IDH^wt^ glioblastoma^25^.

**Fig. 4:**
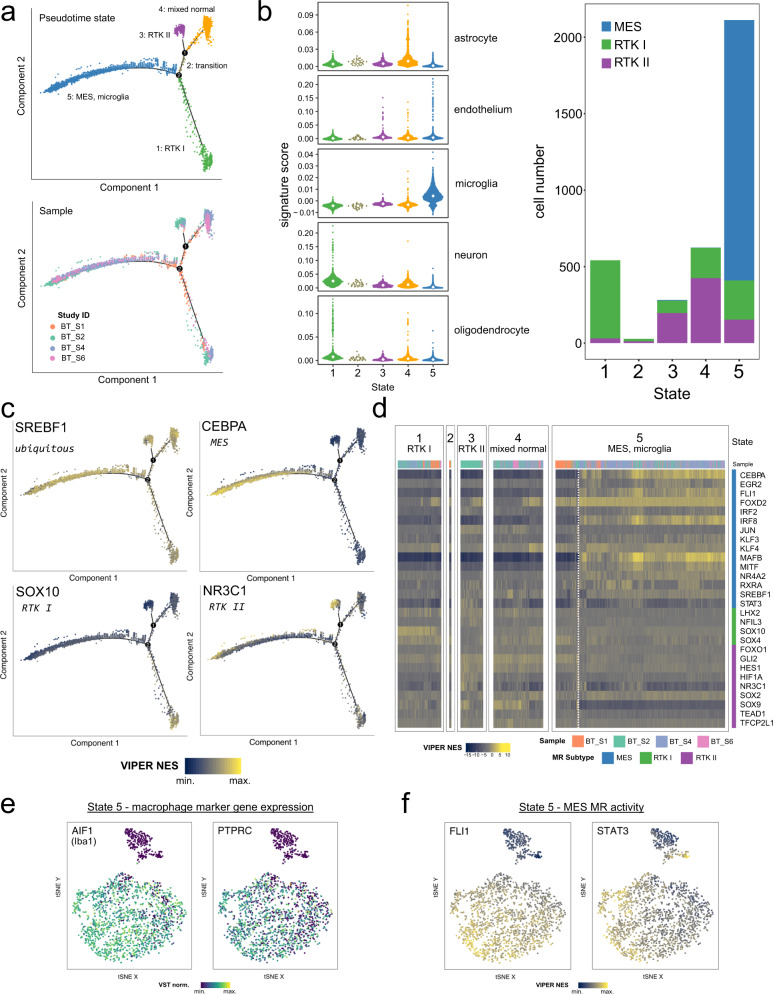
Validation of core regulatory circuitry Master Regulator predictions using glioblastoma single-cell RNA-seq. **a** Pseudotime trajectory inferred with monocle using QC-filtered single cells from the Darmanis (2017)^25^ glioblastoma dataset. At top, the cells are coloured by the inferred pseudotime state. Below, cells are coloured by their source sample in the original study. **b** Assignment of glioblastoma subtype and normal cell identities to pseudotime states. Normal brain cell type signature scores (McKenzie et al., 2018)^28^; median scores are indicated by the white diamond; left), and subtypes assigned based on the VIPER-inferred activity of CIMP^-^ CRC MRs (*n* = 25 analysable MRs) in the TCGA gene regulatory network were calculated for each cell. **c** Visualisation of relative MR activity (*SREBF1*, ubiquitous, top left; *CEBPA*, MES, top right; *SOX10*, RTK I, bottom left; *NR3C1*, RTK II, bottom right) on the pseudotime trajectory. The scaled VIPER NES was calculated for each MR using each cell’s expression profile and the RTN-derived regulons. **d** Heatmap visualisation of VIPER NES for the *n* = 28 CIMP- subtype CRC MRs, split by state. Each column corresponds to a single cell and is annotated with the source tumour sample. Within each state, samples were clustered by their activity profiles. The dashed white line delineates two subpopulations of cells in state 5 with differing MR activity. **e** tSNE projection of the MES tumour and TAM/microglial single cells in pseudotime state 5 (*n* = 2112) using the MES CRC MR (*n* = 13) activity matrix. Each cell is coloured by its relative expression of the macrophage markers *AIF1* (left) and *PTPRC* (right). **f** As in (**e**), but coloured by the scaled MR activity (VIPER NES) of *FLI1* (left) and *STAT3* (right).

Following cell quality control and filtering, we performed a pseudotime analysis^27^. Cells formed a 5-state trajectory, consisting of four terminal branches and one intermediate state (Fig. 4a). It is important to note that we did not distinguish between tumour and normal cells in this pseudotime analysis, and therefore tumour and normal cells with similar gene expression programmes will group together. Therefore, we annotated each state by scoring each cell for a set of normal brain cell signatures^28^ and assigning cells a glioblastoma subtype based on VIPER-calculated MR activity (Fig. 4b). Based on these results and examination of individual marker genes, each terminal branch was assigned the following identity: state 1 represents RTK I tumour cells and normal oligodendrocytes; state 3 are RTK II tumour cells and normal astrocytes; state 4 is a mixture of normal cell types; and state 5 consists of MES tumour cells and macrophages.

We next visualised relative MR activity scores on the pseudotime trajectory, to confirm that the subtype predicted in our CRC analysis is consistent at this single-cell level (Fig. 4c). While some MRs exhibited ubiquitous activity (*SREBF1*), we observed that subtype MRs generally have higher activity in the cells of that state (*CEBPA* in MES, *SOX10* in RTK I, *NR3C1* in RTK II), as predicted.

We clustered cells based on MR activity within each state (Fig. 4d), and observed that four MES MRs (*CEBPA, FLI1, MAFB, MITF*) separate two subpopulations within state 5. Interestingly, even within the cells with high activity of these four factors, further MR heterogeneity is observable (e.g. *RXRA*). This suggested us that MR activity might discriminate cell types.

Therefore, we further examined the 2112 cells in state 5. A t-SNE projection of the cells’ MES MR (*n* = 13) VIPER activity matrix splits this state into two sub-clusters. Visualisation of microglial/macrophage marker gene expression (*AIF1*, *PTPRC*; Fig. 4e) identified the major population as these infiltrating immune cells, as expected. This suggests that the remaining population are MES glioblastoma cells. We found that the MRs CEBPA, FLI1, MAFB, MITF showed higher activities in this immune population, whereas STAT3, which was previously reported to induce mesenchymal transformation in NSCs^13^, appears to be active in both populations (Fig. 4f). Therefore, our analysis of scRNA-seq data were consistent with the analyses of bulk tissue data, in that it identified three tumour branches to which the respective subtype MRs could be assigned. Furthermore, for the MES subtype, which is known to be enriched with infiltrating immune cells, we were able to use scRNA-seq data to compare MR activity in tumour cells and tumour-associated macrophages.

### Loss of SOX10 results in an RTK I-to-MES transition

To independently validate our CRC analysis, we analysed gene expression-based GB regulatory networks using the Reconstruction of Transcriptional Networks and Analysis of Master Regulators (RTN) package^29^. Along with the previously used TCGA network (Fig. 3d; Supplementary Data 8), we used a network inferred using an additional 569 microarray samples (Supplementary Data 9) to cross-validate our predictions. We defined glioblastoma subtype RNA expression signatures (Supplementary Fig. 2 and Supplementary Data 10), and used these to identify 117 subtype MRs that were statistically (two-sided *t*-test) significantly active in the same subtype, with the same direction of activity, in both networks (Supplementary Fig. 3 and Supplementary Data 7, 11).

**Fig. 5:**
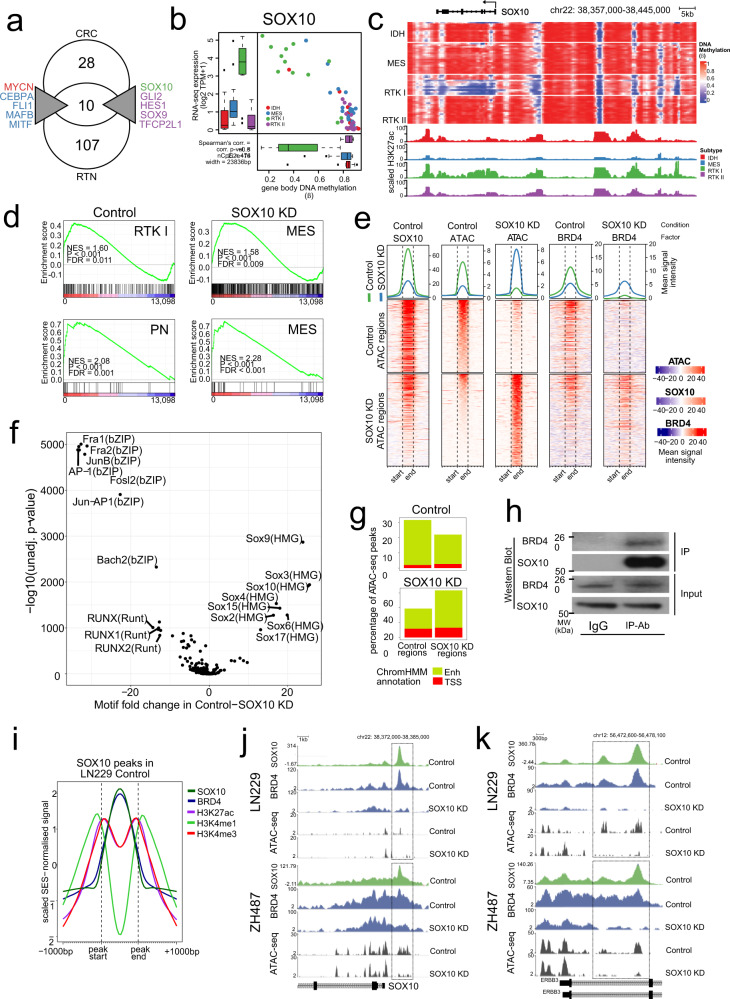
*SOX10* is a Master Regulator of the RTK I subtype. **a** Consensus Master Regulators. **b** Correlation of DNA methylation and *SOX10* expression within the *SOX10* gene body. Boxes in Tukey plots correspond to the 25th, 50th/median and 75th percentiles; whiskers denote 1.5× the IQR from the median. Points mark outliers beyond 1.5× IQR.IDH, *n* = 12; MES, *n* = 19; RTK I, *n* = 12; RTK II, *n* = 17 samples. **c** Epigenome landscape of *SOX10* in glioblastoma. Per-sample methylation (WGBS beta, top) and subtype mean H3K27ac intensity (SES-normalised, bottom) are shown. **d** GSEA plots showing enrichment of proneural and mesenchymal gene signatures in control and SOX10 KD LN229 cells. Upper row: limma subtype signatures of tumour cell-specific gene expression; lower row: tumour cell-specific signatures of Wang et al. (2017)^5^. GSEA-calculated statistics for gene set enrichment are shown. *P*-values (all < 0.001) and FDR values were computed empirically using a permutation test (*n* = 1000 permutations) based on the enrichment score. **e** EnrichedHeatmap visualisation of genome regions with differential chromosome accessibility in LN229 control and SOX10 KD cells, as identified by ATAC-seq analysis. SES-normalised signals of SOX10 ChIP-seq, ATAC-seq and BRD4 ChIP-seq are displayed. Signal intensity is shown in the blue–red heatmaps, where each row shows a single ATAC peak, as indicated by the vertical dashed lines, and 1 kbp further 5′ and 3′. The lineplots at the top of each heatmap display the mean signal intensity across all the regions in that category (control: green; SOX10 KD: blue). **f** Volcano plot of de novo motif finding with HOMER from the differentially bound ATAC-seq peaks in LN229 cells. The significantly enriched motifs are labelled. **g** ChromHMM annotations of LN229 ATAC-seq peaks. Active TSS (E01–E04) and Enhancer (E07–E11) states in the NT (left) and SOX10 KD conditions (right) are shown. **h** Western blot of SOX10 and BRD4 co-immunoprecipitation in the cell line LN229 (two independent experiments). **i** Factor co-occupancy at SOX10 peaks in LN229. The SES-normalised signal for peak regions and 1 kbp up and downstream for SOX10, BRD4, H3K27ac, H3K4me1 and H3K4me3 were separately scaled. **j, k** Changes in SOX10 and BRD4 binding and ATAC-measured chromatin accessibility at the RTK I subtype genes *SOX10* (**j**) and *ERBB3* (**k**). SES-normalised ChIP-seq and ATAC-seq signal is shown in the NT and SOX10 KD conditions in the LN229 (top) and ZH487 (bottom) cell lines. The boxes indicate regulatory regions where SOX10, BRD4 and ATAC-seq signal change in a co-ordinated manner.

Overlap of these RNA-based predictions with chromatin-based CRC MRs (Fig. 3d) gave a consensus list of 10 MRs (Fig. 5a), from which we selected *SOX10* for functional validation. SOX10 is an oligodendroglial lineage transcription factor^30^, and regulates a distinct epigenetic state that is linked to chromatin remodelling and therapy resistance in melanoma, which, like glioblastoma, originates from the neural-crest^31,32^. This evidence of its involvement in clinically relevant state transitions makes SOX10 a particularly interesting candidate to study epigenetic control and remodelling of subtype gene regulation in glioblastoma. In addition, SOX10 has been shown to affect cell fate decisions in neural lineage development in mice, and was described to antagonise the function of the transcription factor NFIA in driving astrocytic differentiation in normal development and later, overexpression in mouse tumour models^33,34^.

*SOX10* is over-expressed in RTK I tumours (Fig. 5b), correlating with genic hypomethylation and increased H3K27ac signal (Fig. 5c). We screened glioblastoma cell lines and selected two lines exhibiting high *SOX10* expression and promoter hypomethylation, characteristic of RTK I patient samples for use as in vitro models: ZH487, a primary glioblastoma cell line that we established from an RTK I patient sample, and the conventional primary glioblastoma cell line LN229. Furthermore, SOX10 ChIP-seq identified a large number of binding sites that are shared by the two cell lines, showing that these are appropriate models of SOX10 activity (Supplementary Fig. 4).

Suppression of SOX10 expression (Supplementary Fig. 5a) leads to extensive changes in RNA expression that we analysed by gene set enrichment analysis using our subtype-specific gene signatures, from which we selected only the tumour-specific genes (Supplementary Data 12) using a published gene list^5^. This analysis showed that SOX10 suppression resulted in RTK I-to-MES transition in the LN229 and ZH487 cell lines. We confirmed this finding applying the proneural (PN), mesenchymal (MES) and classical subtype gene signatures that selectively account for tumour cell-intrinsic effects (Fig. 5d and Supplementary Fig. 5b). Consistent with this finding, VIPER-inferred activity levels of the RTK I (*n* = 3, excluding SOX10) and MES (*n* = 15) MRs correlated with the control and SOX10 KD conditions (Supplementary Fig. 5c). The observed RTK I-MES transcriptomic transition was accompanied by increased cell invasion in trans-well invasion assay and organotypic ex vivo brain slice assays^35^ (Supplementary Fig. 5d, e), suggesting that an RTK I-to-MES transition had indeed occurred.

### SOX10 repression remodels the glioblastoma enhancer landscape

SOX10 has been implicated as a chromatin modifier^36–38^, suggesting us that the effects of SOX10 loss in this RTK I-to-MES transition may be mediated via chromatin changes. This was supported by ATAC-seq analyses showing that chromatin accessibility significantly (two-sided *t*-test) decreased at RTK I MR loci including *SOX10, SOX8* and *ERBB3*, and increased at MES MR loci such as *RUNX2*^13^, *FOSL2 and SERPINE1* following SOX10 suppression (Supplementary Data 13, 14). Consistently, SOX10 binding sites identified in ChIP-seq data were preferentially located in genomic regions with increased accessibility in the control rather than in SOX10 KD cells (89% vs. 49%) (Fig. 5e and Supplementary Fig. 6a). Differential motif enrichment analysis of these ATAC-seq regions found enrichment of SOX motifs (SOX9, SOX10, SOX3, SOX4, SOX15, SOX2, SOX17) in control, and predicted MES MR motifs (Fosl1/2, Jun-AP1, RUNX, TEAD) in SOX10 KD cells (Fig. 5f and Supplementary Fig. 6b).

ChromHMM annotation of ATAC-seq peaks revealed chromatin-accessibility changes to preferentially affect enhancer, but not TSS, states (Fig. 5g), indicating the importance of enhancers for subtype identity and agreeing with our analysis of subtype LMRs (Fig. 2). To verify that SOX10 binds to active enhancers in RTK I cells, we analysed the occupancy of Bromodomain containing 4 (BRD4) protein, a marker of active enhancers^39^. Mapping of BRD4 binding to the ATAC-seq regions showed redistribution of BRD4 following SOX10-mediated changes in chromatin accessibility, confirming the remodelling of the active enhancer landscape (Fig. 5e). JQ1 inhibition of BRD4 binding was sufficient to block up-regulation of the MES MR RUNX2 following SOX10 KD, suggesting that the RTK I-to-MES transition is dependent on enhancer dynamics (Supplementary Fig. 6d–f). Co-immunoprecipitation (Co-IP) confirmed that SOX10 and BRD4 physically interact (Fig. 5h), and SOX10 binding sites showed strong BRD4 binding and histone modifications typical of active enhancers (Fig. 5i and Supplementary Fig. 6c), suggesting that SOX10 recruits this co-factor to RTK I active enhancers. Consistent with this hypothesis, we observed loss of BRD4 binding and chromatin accessibility at regulatory regions of RTK1 genes following SOX10 repression (Fig. 5j, k).

In summary, these results suggest that SOX10 maintains the RTK I cellular state via direct regulation of RTK I genes. Loss of SOX10 results in chromatin accessibility changes, enhancer remodelling and the release of BRD4 from RTK I enhancers. At this point it remains unclear, which factors are recruiting BRD4 to MES enhancers, leading to the manifestation of the MES cellular state.

### SOX10 repression results in mesenchymal phenotype in vivo

**Fig. 6:**
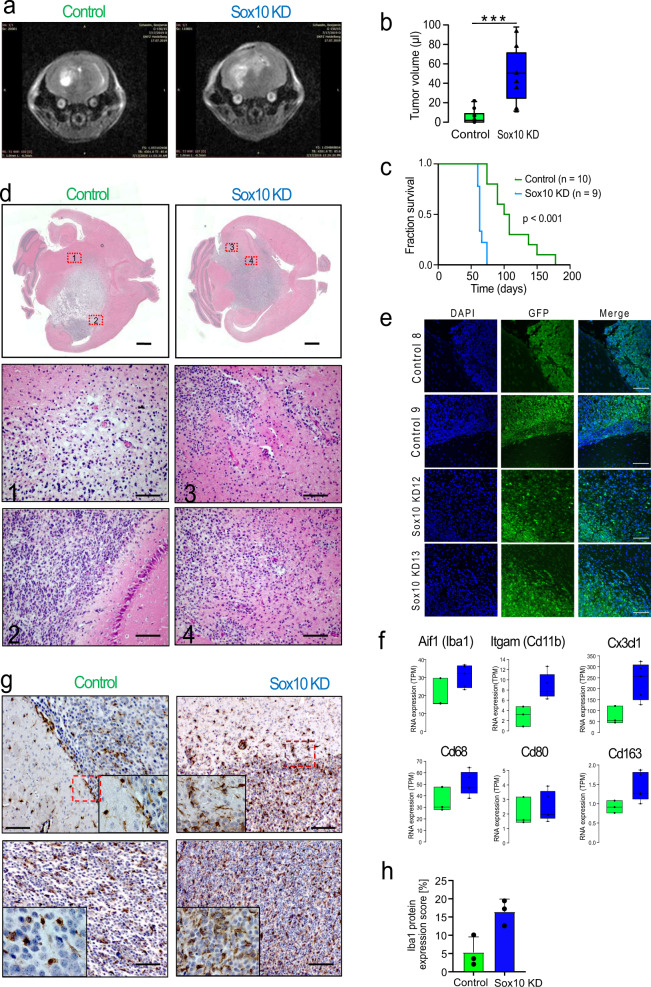
Loss of SOX10 induces a mesenchymal phenotype in vivo. **a** Representative MRI images of mouse brains bearing control (left) and SOX10 KD (right) tumours, taken 57 days post cell injection. **b** Median tumour volumes (µl) measured using MRI 57 days post-injection. NT: 1.85 µl, *n* = 10 animals; SOX10 KD: 50.3 µl, *n* = 9 animals. One-sided *t*-test, *P* = 8.65e-5. Boxes in Tukey plots correspond to the 25th, 50th/median and 75th percentiles; whiskers denote 1.5× the IQR from the median. Points mark outliers beyond 1.5× IQR. **c** Kaplan-Meier survival analysis. NT: median 104 days, *n* = 10 animals; SOX10 KD: median 63 days, *n* = 9 animals. Two-sided log-rank test, *P* = 4.89e-5. **d** H&E stainings of NT (left) and SOX10 KD (right) tumours were performed for two animals per group. Scale bars indicate 200 and 100 µm in gross and detail views, respectively. **e** Staining of tumour margins in 2 control and 2 SOX10 KD tumours with DAPI and antibodies against GFP, which is expressed only in tumour cells. Scale bars correspond to 100 µm. **f** RNA expression of myeloid and microglia marker genes in *n* = 3 (shNT) and *n* = 5 (shSOX10) tumours. Boxes in Tukey plots correspond to the 25th, 50th/median and 75th percentiles; whiskers denote 1.5× the IQR from the median. Points mark outliers beyond 1.5× IQR. **g** Immunohistochemistry staining of Aif1 (Iba1) at the tumour margin (top) and in the tumour bulk (bottom). Red boxes indicate the areas shown in close-up. Scale bar: 100 µm; two animals per group; 5 fields examined in each animal. **h** Quantification of Aif1 (Iba1) staining. Aif1-positive areas were computed for 10 fields per sample and 3 samples per condition. Mean ± standard deviation are shown; Two-sided *t*-test; *P* = 0.025.

The mesenchymal subtype of glioblastoma has been associated with increased tumour cell invasion and immune cell infiltration in patients^5,9^. We therefore turned to a recently established immunocompetent syngeneic graft mouse model of glioblastoma with the genetic background of neural stem-cell specific Pten/Tp53 double knockout^40,41^ to investigate the role of SOX10 in these phenotypes. Repression of SOX10 resulted in faster in vivo tumour growth and a highly significant decrease in median survival time of engrafted mice (NT: 104 days, *n* = 10; SOX10 KD: 63 days, *n* = 9; *P* < 0.001, Fig. 6a–c).

H&E staining of whole-brain sections showed that control tumours exhibit a fairly defined area of the tumour bulk and a marginal zone of tumour cells invading the surrounding tissue while in the case of SOX10 KD cells tumour boundaries appear more disrupted (Fig. 6d). This impression was supported by staining of GFP, which is expressed only in the tumour cells, showing better-defined tumour margins in control than in knockdown tumours (Fig. 6e). These findings suggest possible increased invasion of the surrounding normal tissue by tumour cells after SOX10 repression, consistent with our in vitro trans-well invasion and organotypic ex vivo brain slice assays (Supplementary Fig. 5d–f).

RNA profiling of microenvironment-related genes revealed increased expression of markers for TAMs and resident microglia (Aif1, Itgam, Cd68 and Cx3cl1), and macrophage M1/M2 polarisation (Cd80 vs. Cd163) in knockdown tumours (Fig. 6f). In addition, immunohistochemistry staining identified increased numbers of Aif1 (Iba1) positive cells in SOX10 KD compared to control tumours (Fig. 6g). At the tumour margins, Aif1-positive cells showed a microglia-like morphology while they appeared more roundish in the tumour bulk (Fig. 6g, top vs. bottom row). In agreement with the RNA expression data, quantification of Aif1 staining showed an increase in tumour-associated macrophage infiltration in the SOX10 KD tumours (Aif1 positive area: NT: 4.57%; SOX10 KD: 17.11%; 10 fields of view in 3 tumours in each condition; *P* < 0.001) (Fig. 6h). In summary, these results show that SOX10 repression causes a phenotypic switch to a mesenchymal state in vivo, resulting in increased immune-cell infiltration and significantly decreased survival time.

**Fig. 7:**
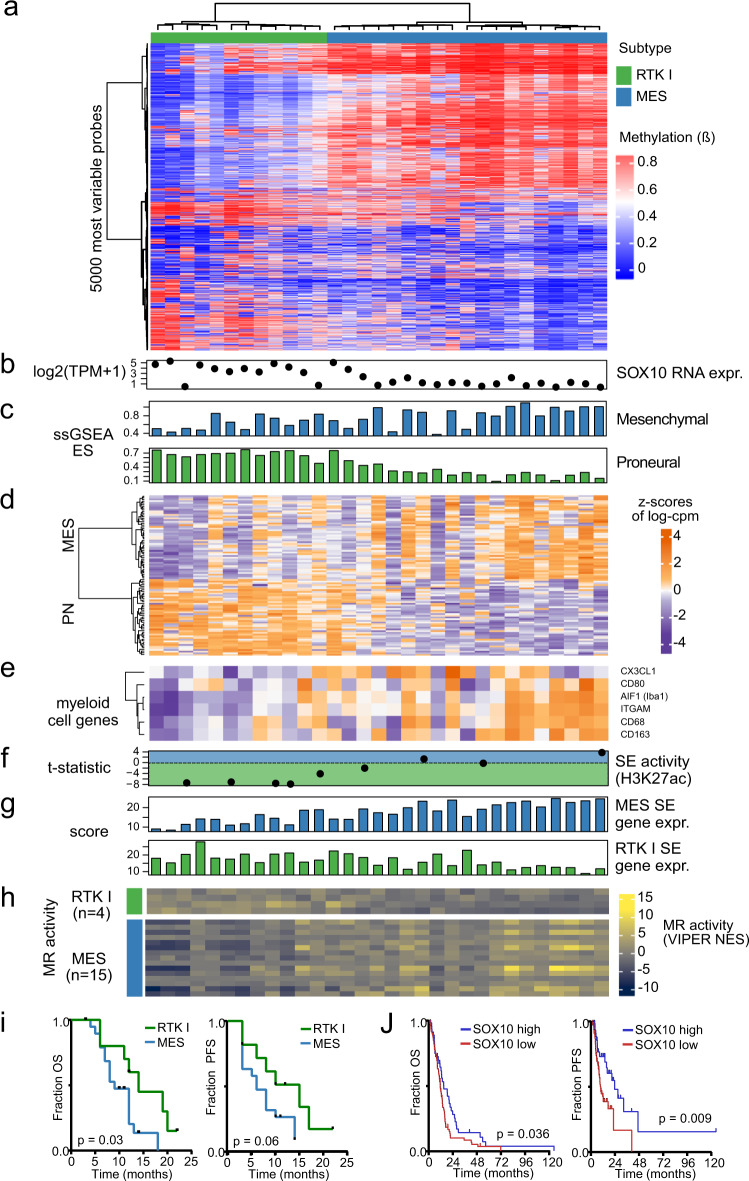
Genetic and epigenetic patterns of RTK I-to-MES transition in primary glioblastoma tissues. **a** Clustering of the 5000 most variable microarray probes in RTK I and MES tumours (*n* = 31) from our cohort identified 3 clusters consisting of RTK I tumours, MES tumours with intermediate genotypes and MES tumours with typical MES genotypes. **b** SOX10 RNA-seq (log_2_ TPM + 1) expression. **c** Wang PN and MES subtype signature ssGSEA score. **d** Relative expression of Wang PN and MES subtype marker genes. **e** Relative expression of myeloid cell marker genes. **f** RTK I and MES SEs differential H3K27ac enrichment (*t*-statistic) R. **g** Expression score of each subtype-specific SE’s target genes (MES: *n* = 422; RTK I: *n* = 279). **h** MR activity (VIPER NES) of the RTK I (*n* = 4) and MES CRC MRs (*n* = 12). **i** Kaplan-Meier survival curves for the RTK I and MES subgroups, considering overall (left panel; *P* = 0.030) and progression-free survival (right panel; *P* = 0.060). **j** Kaplan-Meier survival curves for MES tumours (*n* = 132) of the TCGA glioblastoma cohort stratified by average SOX10 expression. Overall survival (left panel; *P* = 0.036); progression-free survival, (right panel; *P* = 0.009). Group cut-off: average expression; statistical significance was determined by two-sided log-rank test.

Finally, we returned to our primary tumour data to find evidence of SOX10-associated RTK I-to-MES transition in the RTK I and MES patient samples. Clustering of the 5000 most variable microarray probes in these 31 tumours identified 2 subtype clusters showing a gradient of methylation (Fig. 7a). We found that SOX10 expression is higher in RTK I than in MES tumours, and observed the same trend in proneural and MES^5^ gene expression (Fig. 7b–d). In agreement with results in vivo, MES tumours also exhibited higher myeloid marker gene expression than in RTK I (Fig. 7e). The differential H3K27ac enrichment of RTK I and MES SEs, the expression of SE-defined subtype identity genes and MR activity of the RTK I and MES MRs identified in the CRC analysis also correlated with this methylation gradient (Fig. 7f–h). In addition, the RTK I and MES tumour patients of our study cohort showed significantly different patients’ survival, and low SOX10 expression in MES-subtype glioblastoma of the TCGA cohort significantly correlated with adverse prognosis (Fig. 7i, j). These data add further support to the concept of a gradient of SOX10-dependent molecular and phenotypic characteristics in human glioblastoma.

## Discussion

In this study, using chromatin and transcriptome gene regulatory network analyses, we show that glioblastoma subtypes have distinct enhancer landscapes and Master Regulator (MR) repertoires. In patient-derived and adherent cell line models, we found that SOX10 is an RTK I MR, and that its repression results in a transcriptomic and phenotypic RTK I-to-MES transition via remodelling of the enhancer landscape. Finally, we demonstrate that repression of SOX10 in an advanced syngeneic mouse model has a major effect on in vivo phenotypes, including altered growth patterns, increased immune cell content and a significant decrease in survival.

Past methylome studies of glioblastoma have used microarray^2,3^ or RRBS^9^ data that sample a relatively small proportion of the genome. In contrast, our study generated WGBS data from a large number of glioblastoma primary tumours, which allowed us to analyse CpG methylation genome-wide, and describe subtype methylation differences that could not previously be appreciated. We found that the G-CIMP hypermethylation characteristic of IDH glioblastoma is not limited to CpG islands, but affects all genomic features and functional chromatin states. Furthermore, our analysis highlighted the importance of LMR and enhancer methylation for differentiating glioblastoma subtypes.

We next identified the MRs that operate within these enhancer landscapes using complementary analyses of chromatin and transcriptome data generated from primary patient samples, providing the most comprehensive analysis to date. Previously, smaller studies had already provided evidence for the importance of enhancers and bivalent promoters for subtype identity^42,43^; however, these studies used RNA transcription-based subtype classification schemes that did not differentiate between IDH^wt^ and IDH-mutated proneural-subtype tumours, or included the meanwhile abandoned concept of a “Neural” subtype of glioblastoma^2,4^. These classification schemes are only partially consistent with current concepts of glioblastoma subtyping^1–3,5–7,44,45^ and their findings, therefore, difficult to relate to ours.

We validated our subtype MR predictions using published scRNA-seq data from IDH^wt^ tumours^25^. Using this approach, we could separate cell states corresponding to normal cells and the three IDH^wt^ glioblastoma subtypes. Recent single-cell studies of glioblastoma have proposed a variety of cell state models^5,16,18,25,26^. However, xenotransplantation studies have demonstrated that even a single glioblastoma cell can regenerate the cellular heterogeneity of the parent tumour^16^. Our analysis implicates enhancers and subtype MRs as key contributors to this cell state plasticity.

Our complementary analyses identified 10 high-confidence, consensus subtype MRs. We selected the RTK I MR candidate *SOX10* for functional characterisation. *SOX10* is a member of the developmentally important SRY-related HMG-box containing (SOX) family of TFs^46^. In the CNS, *SOX10* is an oligodendrocytic marker gene, and its activity is an example of tumour cells co-opting a developmental pathway to escape the terminal cell differentiation state^34,47^. In melanocytes, SOX10 binds promoters and distal elements^48^ and can recruit chromatin-modifying co-factors such as SMARCA4^38^ and Chd7^36^.

We found that SOX10 maintains the RTK I transcriptomic state via regulation of subtype genes and its loss caused a shift to a mesenchymal phenotype. This transition is dependent on enhancer remodelling, as demonstrated by blockade of BRD4 activity using JQ1 inhibition, a phenomenon that was first described in recurrent glioblastomas^10^. Interestingly, recent data suggest that the reverse, MES-to-RTK I transition might be forced by depletion of the AP1 transcription factor FOSL1 in an NF1-mutant model^49^, supportive of a high level of plasticity of subtype identities. Other work has shown that RTK I-to-MES transition can be induced by therapy and correlates with resistance development^50–54^. Recent data indicate that MES glioblastoma has the worst prognosis of all subtypes^5,9,18,42^. Our in vivo results suggest that loss of SOX10, leading to changes in myeloid cells, may be the underlying cause of this decrease in survival, an interpretation consistent with the correlation of low SOX10 expression and adverse survival that we observed for human MES-type glioblastoma patients.

Notably, loss of SOX10 has also been linked to adverse outcomes in other neural crest-derived tumours. For example, its loss in melanoma leads to transcriptome rewiring and drug resistance^32^. It is striking that neural crest cells undergo an analogous mesenchymal transition during normal development, suggesting that even in highly divergent tissues, hijacking of common developmental pathways by cancer can occur^46,55^.

Neural lineage development has been shown to be regulated by sequentially interacting SOX transcription factors^56^, including SOX2, a reported marker of the proneural glioblastoma subtype and regulator of cell plasticity and astrocytic differentiation^57,58^. In our tumour cohort, SOX2 RNA-expression was about 2× lower in MES compared to other subtypes, but still remained at a very high level, about 3× higher than in normal brain. Furthermore, SOX2 showed strong H3K27ac activation in all glioblastoma subtypes (Fig. 3a), and its RNA expression did not significantly change in the SOX10 knockdown models. We therefore have no evidence on RNA-level to support a subtype-specific role of SOX2 or its relevance in the context of RTK I-to-MES transition induced by the loss of SOX10.

In extensive studies of murine neural developmental pathways and their potential role in human gliomagenesis, SOX9-dependent activation of the transcription factor nuclear factor I-A (NFIA) was shown to antagonise SOX10 function and result in astrocytic differentiation of the tumour cells^33,34^. Although we identified SOX9 as an RTK II MR (Fig. 5a) and observed consistent upregulation of SOX9 in our SOX10 knockdown models, loss of SOX10 for unknown reasons did not result in the upregulation of NFIA RNA expression. This possibly provides an explanation to why we observed RTK I-to-MES rather than RTK I-to-RTK II transition after SOX10 suppression (Fig. 5d and Supplementary Fig. 5b).

Our subtype MR analysis identifies many candidates beyond SOX10 that are known to play roles in CNS development, suggesting a model in which MR activity maintains and interacts with genetic and epigenetic factors to define a glioblastoma cell state. Evidence of plasticity, such as genetic, epigenetic and regulatory features of RTK I-to-MES transition, can be readily visualised in the multiple layers of data that we generated from primary tumours. The role of tumour cell-extrinsic components such as the microenvironment will add further complexity to this tumour cell-intrinsic plasticity, as suggested by our in vivo experiments. In this context, elucidating the mechanisms that promote myeloid-cell invasion and immune suppression will be of particular clinical relevance for the development of immunotherapy approaches. These findings are mirrored by a recent publication showing that knockdown of SOX10 is sufficient to convert a cell from melanocyte-like to mesenchymal-like in melanoma, and demonstrating that microenvironmental cues likely play a critical role in regulating melanoma cell state^59^. If, as is increasingly plausible, subtype plasticity contributes to therapy failure in glioblastoma, drug combinations simultaneously targeting both tumour cell growth and epigenetic plasticity may block the escape of cancer cells to a therapy-resistant state and thus lead to improved patient outcomes.

## Methods

### Primary tissue samples

Snap-frozen primary glioblastoma tumour samples and clinical data were collected at the time of primary diagnosis between 1994 and 2011 at the Burdenko Neurosurgery Institute (Moscow, Russia). Informed consent was obtained from all patients. Use of the material and clinical data for this study was approved by the ethics board at the Burdenko Neurosurgery Institute (Moscow, Russia). The patient cohort consisted of 32 males and 28 females with an average age of 52.5 ± 11 years (mean and standard deviation). Patients with IDH-subtype tumours tended to be younger than patients with tumours of the MES, RTK I and RTK II subtypes (42.6 ± 9.5 vs. 55.0 ± 9.9 years; mean and standard deviation). IDH1/2 mutation status were determined using either pyrosequencing or Sanger sequencing^60,61^. Samples of post-mortem normal brain were purchased from Biocat (Heidelberg, Germany).

### Cell lines and cell culture details

The human glioblastoma cell line LN229 (p53 mut, PTEN wt, p16 del; established from a white, 60-year old female in 1979) was obtained from ATCC (Cat#CRL-2611) and cultured in DMEM supplemented with 10% FCS, Penicillin/Streptomycin and glutamine. ZH487 patient-derived glioblastoma cells were established at the University of Zurich Hospital. ZH487 cells were cultured in Neurobasal medium (Cat#12348017, NBM) supplemented with 2% B27 (Cat#12587010, retinoic acid-free, Invitrogen), EGF (20 ng/ml, AF-100-15, Peprotech), FGF (20 ng/ml, Cat#100-18B, Peprotech) and glutamine (0.5 mM). HEK293T cells were used for lentivirus production and maintained as monolayer cultures in antibiotic-free DMEM supplemented with 10% FCS. All cells were cultured under 10% CO_2_ at 37 °C with humidity. Cell line identities were verified by the Multiplex human Cell line Authentication Test (MCA), and cells were tested for mycoplasma contamination with the Multiplex cell test (both Multiplexion GmbH, Friedrichshafen, Germany).

### In vivo syngeneic mouse model

Animal experiments performed for this study comply with all relevant ethical regulations and were approved by the Regierungspräsidium Karlsruhe, Germany (reference no. G-156-15). The primary mouse glioblastoma cell line with *Pten/Tp53* double knockout was established by the lab of Prof. Peter Angel in the German Cancer Research Center (DKFZ)^40,41^. mGB1 cells were characterised as the Proneural/RTK I subtype based on RNA-seq profiling with high SOX10 expression. mGB1 cells were culture at 37 °C in DMEM/F12 medium supplement with N2 supplement, EGF (20 ng/ml), FGF (20 ng/ml), Penicillin/Streptomycin and glutamine. SOX10 knockdown was carried out using lentivirus transduction with the shSOX10 (TRCN0000244290, Supplementary Table 3). All cells were Puromycin selected and SOX10 knockdown level was RT-PCR validated before injection; 200k cells (shNT and shSOX10, in 1 µl volume) were intracranially injected into adult C57/B6 mice (6 weeks female) brain under anaesthesia with Isoflurane. MRI scanning was performed in the DKFZ MRI core facility on 57 days post-injection.

### Whole genome bisulphite sequencing (WGBS)

Primary GB whole-genome bisulphite library preparation was carried out as described previously^20^. Briefly, 5 μg of genomic DNA was sheared using a Covaris device. After adaptor ligation, DNA fragments with insert lengths of 200–250 bp were isolated using an E-Gel electrophoresis system (Life Technologies) and bisulphite converted overnight using the EZ DNA Methylation kit (Zymo Research). The fragments were PCR amplified using the FastStart High Fidelity PCR kit (Roche) for 6–8 cycles. Library aliquots were then purified and size selected with AMPure beads (New England BioLabs) and quality controlled with a Bioanalyzer (Agilent). Each library was sequenced using 2 lanes on an Illumina HiSeq 2000 in the DKFZ Genomics and Proteomics core facility.

### Whole genome sequencing (WGS)

DNA (500 ng per sample) for whole genome sequencing were submitted to the DKFZ Genomics and Proteomics core facility and the library preparations were carried out using the standard protocol from Illumina. Each library was sequenced using 1 lane on an Illumina HiSeq X Ten.

### RNA sequencing

Primary GB RNA-seq libraries were prepared as described using methods to preserve strand specificity and deplete rRNA. Sequencing was carried out on the HiSeq 2000 platform with 1 lane per sample. All samples profiled by WGBS sequencing, WGS, 450k/Epic methylation microarray and RNA sequencing were genotyped in silico to exclude sample swaps.

RNA-seq of GB cell lines (LN229 and ZH487, Control vs. shOX10) were performed using the polyA-selected RNA-seq libraries preparation protocol with the TruSeq stranded RNAseq Illumina kit by the DKFZ genomics & Proteomics core facility. Libraries were multiplexed-sequenced using 1 lane on a HiSeq 2000 v4, generating 50 bp single-end reads. RNA-seq libraries preparation of mouse tumour samples (shNT, *n* = 3; shSOX10, *n* = 5) were also used in the above mentioned PolyA protocol. Libraries were multiplexed-sequenced using 2 lanes on a HiSeq 2000 v4, generating 50 bp single-end reads.

### ChIP-seq

Then, 10 µg each of H3K27Ac (Cat#AM39133, Active Motif), H3K4me1 (Cat#AM38297, Active Motif), H3K4me3 (Cat#AM39159, Active Motif), H3K9me3 (Cat#AM39161, Active Motif), H3K27me3 (Cat#07-449, Millipore) and H3K36me3 (Cat# AM61101, Active Motif) were used for ChIP library preparation of GB patient samples, which was performed at Active Motif according to proprietary methods. Libraries were multiplexed so that all libraries for each individual IP were sequenced on 1–4 lanes using the Illumina HiSeq 2000 platform. For the LN229 histone mark experiments, LN229 cells (LN229-shSOX10 without (Control) or with doxycycline induction of SOX10 knockdown) were cross-linked with 1% methanol-free formaldehyde for 10 min. After quenching with glycine, cells were washed three times with PBS and the cell pellet was treated with 4 U MNase per 1 × 10^6^ cells for 15 min. MNase was stopped with 10× Covaris buffer and the chromatin was sheared for an additional 15 min with the LE220 Covaris device. The soluble chromatin was then recovered, quantified, and 2 µg chromatin was used in each immunoprecipitation (IP) with 2 µl each of each antibody (as above). Following the IP and washes with Covaris buffer, Li-buffer and TE, chromatin was digested with proteinase K and purified with AMPure beads. The purified DNA was cloned into illumina sequencing libraries with the NEBNext Ultra library preparation kit (NEB) according to standard protocols. For SOX10 and BRD4 ChIP-seq experiments, cells were cross-linked with 1% methanol-free formaldehyde for 15 min and quenched with 0.125 M glycine. Chromatin was isolated by adding lysis buffer and Dounce homogenisation. Collected chromatin was sheared via sonication to an average length of 300–500 bp. Input genomic DNA was prepared from collected chromatin by treatment with RNase, proteinase K and de-crosslinking under heat, and then isolated by ethanol precipitation. Pellets were re-suspended and DNA quantified on a NanoDrop spectrophotometer. Estimated total chromatin yield was calculated based on this amount; 30 µg of chromatin was pre-cleared with protein A agarose beads (Invitrogen), and DNA precipitated using 4 µg of antibody against SOX10 or BRD4. This DNA was isolated from the beads by washing followed by SDS buffer elution, RNase/proteinase K treatment and de-crosslinking under heat (65 °C overnight incubation). DNA was then purified using phenol-chloroform extraction and ethanol precipitation. Sequencing libraries were prepared and input DNA via standard protocols (enzymatic end-polishing, dA-addition and adaptor ligation) on an Apollo 342 NGS Library Prep system (Wafergen Biosystems/Takara). Prepared libraries were then sequenced (50 bp, single-end) on an Illumina HiSeq 2000.

### ATAC-seq

ATAC-seq was performed in biological duplicates, as previously described^62^. Briefly, viable frozen cells were incubated with Tn5 in 0.1% Igepal CA-630 (37 °C, 30’). Transposition was stopped with EDTA and DNA purified using AMPure beads. After DNA purification, barcodes were added using PCR and DNA re-purified on AMPure beads. These prepared libraries were then sequenced (50 bp, single-end) on an Illumina HiSeq 2000.

### Gene expression microarray profiling of cell lines

DNase-treated total RNA (500 ng) was prepared for gene expression profiling on Affymetrix HG-U133-Plus2 and Illumina HumanHT-12 v4 Expression BeadChip microarrays at the DKFZ Genomics & Proteomics Core Facility. The GSEA results from Fig. 5d and Supplementary Fig. 5b were generated with the microarray data from the Affymetrix Human U133Plus 2.0 platform. For LN229 cells, the control group included the non-treated control and non-targeting sgRNA control, and for the SOX10 knockdown group, three guild RNA targeting SOX10 were used. For ZH487 cells, non-treated control and shNT was used as control with biological replicates and three shSOX10 shRNA were used to achieve SOX10 repression also with biological replicates.

### Methylation microarray data processing and CNV calling

Here, 450k and EPIC DNA methylation array data were processed and analysed as previously described^44^ using the minfi (1.24.0)^63^ and conumee (1.3.0)^64^ Bioconductor packages. In brief, > 500 ng of DNA from snap-frozen samples was used as input material. minfi was used to extract raw signal intensities from IDAT files, and both colour channels corrected for background and dye-bias. Beta values were calculated using an offset of 100. CNVs were called using the standard conumee procedure, using two sets of 50 control samples with balanced CN profiles. Copy number aberrations were called from the conumee-processed values using the following numerical thresholds: for *x* < −1, as a deletion; −1 < *x* < −0.2 as a loss; −0.2 < *x* < 0.2 as no copy number change; 0.2 < *x* < 1 as a gain; *x* > 1 as an amplification.

### Subtype classification of patient samples using methylation microarrays

From the previous GB classification,^3^ 8000 probes (Supplementary Data 6) were used to cluster the methylation microarray data. We used only probes that appear on both 450k and EPIC microarrays (*n* = 7386). The methylation beta value matrix was used to calculate the sample pairwise Euclidean distance. This distance matrix was then used to hierarchically cluster samples using the ‘ward.D’ method.

### Clustering of MES and RTK I patient sample methylation microarrays

The MES (*n* = 19) and RTK I (*n* = 12) glioblastomas were clustered together (Fig. 7) to identify substructure in these subtypes. The 5000 most variable probes (by SD) were selected and the samples and probes hierarchically clustered using the Euclidean distance and the ‘ward.D’ method.

### RNA-seq processing and expression quantification

Reads were aligned to the appropriate reference genome (hg19/mm10) with the Gencode reference transcriptome (v19/M2) using STAR (v2.3.0e). Read counts for each gene were quantified as the total number of reads mapping to exons using htseq-count (0.6.0) for human or featureCounts (Subread v1.5.3) for mouse samples. Gene expression values for each sample were quantified using the transcripts per million (TPM) metric.

### Tumour RNAseq and limma subtype gene signature analysis

Raw read counts per gene were pre-filtered, retaining those genes with > 10 reads in > 6 samples for further analysis. Normalisation factors for the counts were calculated using the *calcNormFactors* function in ‘edgeR’ (3.20.1). *voomWithQualityWeights* from ‘limma’ (3.34.4) was used to transform the raw counts. limma differential expression analysis was used to compare the gene expression of each GBM subtype versus the other 3 (example contrast: IDH / ((MES + RTK I + RTK II)/3)). Genes were defined as significant for a subtype if they passed a BH adjusted *P-v*alue threshold of 0.001. Signatures from isolated mouse normal brain cell populations^63^ were used to compare subtypes to normal cell populations, and to GB subtype signatures. The enrichment of each gene set was tested in our samples using the ‘ssgsea’ method of the ‘GSVA’ R package (1.26.0), using TPM expression values. ESTIMATE^65^ (1.0.13) was used with default settings to determine immune and stromal cell content. Genes specifically up-regulated in a subtype (log FC > 0, adj. *P-v*alue < 0.05) were functionally annotated with Gene Ontology terms (“org.Hs.eg.db”, 3.5.0) using the *enrichGO* function from the R package “clusterProfiler” (3.6.0). MR activity in network A was inferred using the ‘R’ package VIPER (1.14.0; see below for further details).

### WGBS processing

For each sample, reads were mapped to the human genome (hg19) with bwa-mem (0.7.8) with a customised WGBS pipeline^20^. CpGs overlapping variable sites with a minor allele frequency higher than 0.25 were removed. Low coverage CpGs with 2 or fewer reads in more than 50% of the cohort were also removed from the analysis. The mean methylation of the two cytosines in a CpG dinucleotide (one C on forward strand and the other on reverse strand) was calculated by weighting their CpG coverage, i.e. *m* = (*m*_1_*c_1_ + *m*_2_*c_2_)/(*c*_1_ + *c*_2_), where *m*_1_ and *m*_2_ are the number of methylated CpGs of the two neighbouring cytosines and *c*_1_ and *c*_2_ are the corresponding CpG coverage. Similarly the mean coverage for the CpG dinucleotide is calculated by weighting the coverage itself: *c* = (*c*_1_*c_*1*_ + *c*_2_*c_2_)/(*c*_1_ + *c*_2_). Finally, the bsseq R package (1.10.0) was applied to smooth the methylation data and impute the missing methylation values with default parameters.

### Methylation feature (DMVs, LMRs, PMDs) analysis

The segmentation of the methylation features, partially methylated domains (PMDs), lowly methylated regions (LMRs) and DNA methylation valleys (DMVs) were performed by a customised pipeline. Further details can be found in Supplementary Methods. Briefly, chromosomes were split into blocks based on inter-CpG distance, and the mean and standard deviations of methylation of each block used to classify segments into low, intermediate and high methylation separately for each sample. DMVs and LMRs were then defined based on the characteristics of each block and its neighbours. PMDs were called using MethylSeekR (1.14.0)^66^ with 10 kbp minimum width. Subtype consensus regions (DMVs, LMRs, PMDs) were defined as segments with a cross-sample coverage of at least 4. Neighbouring LMRs were merged if the inter-LMR distance was less than 1 kb. Enrichments of other genomic features (e.g. CGIs, ChromHMM annotations) were computed by calculating the Jaccard Coefficient for the base-pair length of the two feature sets. This statistic was compared to a background of 1000 CpG content-matched regions to calculate *z*-scores and *P-v*alues for the enrichment. Classification of samples based on methylation patterns (enhancer and LMR methylation) was done using a projection of the methylation matrix using uniform manifold approximation and projection (UMAP)^67^.

### Subtype sharing methylation features

For each type of methylation features (DMVs, LMRs, PMDs), consensus methylation features in each subtype were first determined by selecting the genomic regions that occurred in more than 50% of samples in that subtype. The extent of subtype sharing methylation features was calculated as the fraction of the total width of regions that occur in 1, 2, 3 or 4 subtypes.

### Chromatin state enrichment

For one set of methylation features and one set of genomic regions with a certain chromatin state, the Jaccard coefficient was used as the measurement of the overlap of two sets of regions, which was calculated as total base pairs of the intersected regions divided by the total base pairs of union of the two sets of regions. The significance of the Jaccard coefficient was calculated by permuting the methylation features restricted in a specific genomic background where the average CpG content was similar as in the methylation features. The selection of background regions was applied as follows: The methylation features were first split into small windows where the window size denoted as *w* was calculated as the 25^th^ quantile of all widths of the methylation features. *w* was additionally rounded to the thousand digit. The window size is set to 10 kb if it is larger than 10 kb, and it is set to 1 kb if it is smaller than 1 kb. To the fact that small windows might cause bias for the calculation of CpG content due to the sparsity of CpG distribution, windows with a width less than *w*/4 were removed. For all the windows after filtering, the CpG content was defined as number of CpG sites per 1 kb window and denoted as *p*. To find proper background regions in the genome, the genome was split by windows with width *w* and CpG content was calculated for each window. Background windows with CpG content between the 5^th^ percentile and the 95^th^ percentile of *P* were finally selected as background regions. The methylated regions were randomly permuted within the background regions by using bedtools (v2.27.1) for 1000 times. In each permutation, the Jaccard coefficient was calculated. Finally, the *z*-score calculated as (*s*-*μ*)/*σ* was used as the measurement of the enrichment, where *s* is the Jaccard coefficient for the two sets of regions, *μ* and *σ* are the mean and standard deviation of the Jaccard coefficient in the random permutations.

### ATAC-seq and ChIP-seq processing

ATAC-seq and ChIP-seq datasets were processed using a custom pipeline implemented in Snakemake (v. 3.13.3). Briefly, reads were trimmed using the Trimgalore tool (https://github.com/FelixKrueger/TrimGalore) and aligned using Bowtie2^68^ (v. 2.3.4.3) with standard parameters. Duplicates and multi-mapping reads were removed using the samtools package and the XS flag in the bam files. For the ChIP-seq data, input control (tumours: WGS; LN229: H3 ChIP-seq) and corresponding IP datasets were scaled using the SES method and converted into a bigwig track using the bamCompare tool of the deepTools2 suite^69^. For the ATAC-seq data, genome-wide coverage was calculated. Peaks were called using the callpeak mode in MACS2 (v. 2.1.1.20160309) (https://github.com/taoliu/MACS) for broad and narrow peaks. In addition, SICER^70^ was used to call peaks using the gap 600 and window 200 parameters. Various QC parameters (FRiP, PCR bottleneck coefficient, cross-strand correlation) were determined according to the ENCODE guidelines^71^. In addition, visual QC was performed using the signal profile at TSS of annotated genes and the fingerprint method from the deepTools2 suite.

### Chromatin segmentation with ChromHMM

Chromatin segmentation was defined using the ChromHMM (v. 1.19) tool. ChIP-seq (H3K27ac, H3K27me3, H3K36me3, H3K4me1, H3K4me3, H3K9me3) and corresponding input data were binarized using ChromHMM’s “BinarizeBam” command. The Roadmap Epigenome 18-state model^19^ was used to segment the genome of each sample. For the tumours, the consensus state for a subtype was defined as the state with the highest frequency in a given segment, and a minimum frequency of 50%.

### Superenhancer analysis

The union of H3K27ac peaks for each subtype’s samples were used as input regions for the ROSE2 superenhancer analysis pipeline, stitching together regions within 12.5 kbp of each other. Sample H3K27ac signal was calculated using ‘bigWigAverageOverBed’ (v2), and enhancers were ranked by the subtype average enrichment. SEs were defined using the default parameters for ROSE2. Subtype SEs were defined by combining all four subtype SE lists and then performing ANOVAs on the H3K27ac signal intensities, with a minimum log fold change of 1 and a Benjamini–Hochberg adjusted *P*-value threshold of 0.1. For comparisons of MES and RTK I SE activity, two statistics were calculated. Firstly, from the H3K27ac signal intensities, a *t*-statistic based on the relative distributions of signal in the two subtypes’ SEs was computed. Secondly, subtype-specific SE lists for MES (*n* = 422) and RTK I (*n* = 279) were defined as those SEs that have no overlap with any other subtype’s SE. A “subtype-specific SE gene score” was calculated using the mean expression of these targets. The comparison between SEs and subtype LMRs were done by overlapping SEs and LMRs requiring a 50% overlap of the LMR to count it as an overlap.

### Core regulatory circuit analysis

Core regulatory circuits were determined using a modified version of the CRCmapper tool^72^. Instead of assigning the SE to the closest gene as implemented in the original CRCmapper tool, we computed the Spearman correlation of the H3K27ac signal on the SE over all samples, with the gene expression of all genes located within 500 kb around the SE across the same set of samples, provided the SE and the gene are located in the same topological associated domain (TAD). We assigned the highest correlated gene (within the range and within the same TAD as the SE) as the SE target gene. The rest of the CRCmapper procedure remains as implemented in the original tool: briefly, sets of autoregulatory TFs are identified by selecting TFs that are target genes of SE (where the target gene is determined as described above), under the condition that these SEs contain binding motifs for the corresponding TF. Then, cliques of autoregulatory TFs are identified in which the SEs contain binding motifs for all other TFs in the clique.

### Gene regulatory network inference with RTN

Two cohorts of glioblastoma gene expression microarray data were collected: A (from TCGA; *n* = 525 samples profiled on the Affymetrix HT HG-U133A)^2^ and B (samples with the pathological diagnosis “glioblastoma” in the metadata from the following five studies: E-MTAB-3073, GSE4290, GSE7696, GSE16011 and GSE43378; *n* = 569 samples profiled on the Affymetrix HG-U133A Plus2)^73–77^. The raw data were read into R and normalised using the ‘gcrma’ package (2.50.0). Study-associated batch effects were removed from cohort B using the *ComBat* function in ‘sva’ (3.26.0), specifying the study ID as the ‘batch’ option. ‘RTN’ (2.3.4) was used for the following steps of the analysis. Firstly, as the expression of any single gene can be measured by multiple microarray probes, the probe with the highest coefficient of variation in the expression matrix was kept for analysis. Regulatory relationships (‘edges’) between *n* = 1333 TFs (classes ‘a’, ‘b’ and ‘other’ as defined in^69^) and target genes were inferred using the ARACNe algorithm^78^. The direction of TF-target gene regulation (positive or negative) was inferred using Pearson correlation. TF-target edge *P*-values were calculated by permuting the Mutual Information matrix 1000 times, retaining edges with a BH-adjusted *P*-value < 0.01. The network was bootstrapped 100 times and TF-target edges found in 95% of the bootstrap samples retained. Finally, indirect TF-target edges were removed using the Data Processing Inequality (DPI) filter with a tolerance of 0.

### Identification of subtype Master Regulators with RTN

The limma subtype gene expression signatures were used to pre-filter potential subtype MRs using the *tna.mra* (BH adjusted *P*-value < 0.05) function in ‘RTN’. TFs passing the MRA pre-filtering step were then tested in a 1-tail GSEA using the *tna.gsea1* function, using the limma-voom calculated log fold change as the GSEA phenotype. TFs regulating fewer than 15 genes were removed. Significant TFs with a BH-adjusted *P*-value < 0.01 (tested in 10,000 permutations) were retained. Subtype MRs were then identified using the 2-tailed GSEA test as implemented in the *tna.gsea2* function, using the limma-voom calculated log fold change as the GSEA phenotype and the TF regulons inferred in the transcriptional network as the gene sets. TFs with a BH adjusted *P*-value < 0.01 (calculated using 10,000 permutations) were called as significantly active in that subtype. Common network MRs (*n* = 117) were defined as those passing this significance threshold within the same subtype, with the same direction of activity as measured by 2-tail GSEA differential Enrichment Score (dES), in both networks. The 2-tail GSEA dES in the two networks were calculated for each subtype signature for each of the consensus MRs. The results were visualised with the ‘ComplexHeatmap’ package. Finally, the two networks (A and B) were compared as follows: 2-tail GSEA dESs for each subtype signature were calculated for all TFs in both transcriptional networks (*n* = 512). The correlation of dESs between the two networks was calculated using Spearman’s rank correlation and visualised using the ‘ggplot2’ package (2.2.1).

### Single-cell RNA-seq analysis

The counts matrix from a published dataset (GSE84465) was analysed using the ‘monocle’ (2.10.1) package in R (v3.5.1). Briefly, the most variably expressed genes with a minimum mean expression of 0.1 were used to reduce dimensionality in a tSNE (first 4 principal components in 3 dimensions, regressing out the number of genes detected and the patient of origin), and cells clustered (*ρ* = 40, *δ* = 20). This cell clustering was used to identify genes detected in at least 10% of cells and differentially expressed between clusters, and the top 1000 ranked by statistical significance were used as the ordering genes for pseudotime analysis (again regressing out the number of genes detected and the source tumour). Normalised expression values (variance-stabilising transformed, VST) were used in downstream analyses. Normal brain cell population signatures (astrocytes, endothelia, microglia, neurons, oligodendrocytes) from McKenzie et al.^28^ were used to score cells. Briefly, for each gene in a signature, a background was defined consisting of the 100 genes with the smallest absolute expression difference. This matched background was subtracted from the expression value for each signature gene, and the sum of all of these background-corrected signature gene expression values was defined as the score. The Bioconductor package ‘viper’ (v1.14.0)^24^ was used to infer single-cell MR activity. The VST-normalised expression matrix was pre-filtered to remove genes with expression SD in the bottom quartile. MR regulons from network A were used to calculate the VIPER NES, and visualised in tSNE and pseudotime plots. MR activity profiles of VIPER NESs were visualised in heatmaps using ‘ComplexHeatmap’ (1.18.1), using Euclidean distance and the average clustering method. Subtype MR scores were defined by transforming each MR’s NES into *z*-scores, and then calculating the mean for all MRs of that subtype; the predicted subtype for a cell was the subtype with the maximum mean *z*-score. Cells in pseudotime State 5 (MES, microglia) were separated using tSNE (‘Rtsne’ 0.13) on the MES MR activity matrix inferred with network A. Gene expression (VST-normalised) and MR activity (network A) were visualised as for the pseudotime analysis. The RTK II MR ZBTB7C was not included in this analysis since its regulon was too small in RTN network A, which was used in this context.

### Cell line expression microarray data processing and analysis

Raw Affymetrix microarray data were read into R and normalised using the ‘gcrma’ R package (2.50.0). Probes without an annotated gene were removed from the analysis, and the batch effect removed using *ComBat* from the ‘sva’ package for the ZH487 samples. Separately for each cell line model, samples were combined into two groups: control (untreated and non-targeting controls) or knockdown. Standard ‘limma’ differential expression analysis between the control and knockdown groups was performed, and genes with an adjusted *P*-value of < 0.05 were defined as significantly dysregulated. MR activity was inferred using each sample’s processed expression profile as input for VIPER (v1.14.0) analysis with network A. limma-calculated log FC profiles from LN229 and ZH487 cell lines with and without SOX10 repression were analysed by ‘GSEA’ (v3.0). Glioblastoma signature gene sets (Verhaak_glioblastoma: Proneural, Neural, Mesenchymal and Classical) were downloaded from the GSEA website (http://www.broadinstitute.org/gsea/). Results were visualised using Volcano plots. The activity of the MES and RTK I CRC MRs for these samples was calculated using VIPER and the RTN-inferred TCGA network A (*n* = 525 samples), taking the average of replicates for each condition; conditions’ MR activity profiles were clustered using the Euclidean distance and the ‘ward.D2’ method. Significance analysis of TF activities upon SOX KD (Fig. S5C) was done using a two-sided *t*-test to compare WT and SOX10 KD TF activities for each TF.

### ChIP-seq and ATAC-seq analysis

MACS2 peaks calls were used as the input set of regions for this analysis in R (v3.4.3). For ATAC-seq, a consensus peakset was defined from the two biological replicates for each condition by taking the merged peaks generated by the *findOverlapsOfPeaks* function from the R/Bioconductor package ChIPpeakAnno (3.12.4). Signal intensity was calculated using ‘bigWigAverageOverBed’, using the SES-normalised.bigWig file for the factor of interest and the bed file of regions of interest, and visualised using the ‘EnrichedHeatmap’ package (1.9.2). Separately for the two cell lines, differential ATAC peaks were identified using the R/Bioconductor package ‘DiffBind’ (2.6.6) using an FDR threshold of 0.05. Peaks were functionally annotated based on the largest state overlap in a comparison to LN229 cell line ChromHMM states. States were collapsed to the following summary states: E01-E04 were defined as TSS; E07-E11 were defined as Enh.

### ATAC-seq motif finding

HOMER (v4.9.1) de novo motif finding was used with the default settings, apart from defining the background to be the union of ATAC peaks in both conditions (control + shSOX10) for that cell line.

### Visualisation of genomic data

The circlize package (0.4.6)^78^ was used for the circular visualisation of genome-wide methylation differences and chromatin states transitions. The ComplexHeatmap package (1.19.1)^79^ is used for visualisation of heatmaps and complex summary plots. The EnrichedHeatmap package (1.9.2)^80^ is used for visualisation of epigenetic signals at genomic regions. Genome browser views were visualised using the WashU Epigenome Browser or Gviz (1.22.3)^81^. The epik package (https://github.com/jokergoo/epik) is used for general integrative visualisation and analysis.

### SOX10 knockdown systems

CRISPRi SOX10 knockdown LN229 cells were used for gene expression microarray experiments; 20 nt sgRNA sequences were designed using the CRISPR web design tool (http://crispr.mit.edu), targeting a genomic window of −50 to +200 bp relative to the transcription start site as defined by the NCBI RefSeq database. sgRNA oligonucleotides were cloned into a 5′ BstXI-BlpI 3′ digested backbone of a pU6-sgRNA EF1Alpha-puro-T2A-BFP expression plasmid by adding additional sequences to obtain compatible sticky ends (see Supplementary Table 3 for oligonucleotide sequences). Stable dCas9-expressing LN229 cells were transduced with lentivirus containing gRNAs targeting SOX10, or lentivirus containing negative guide RNA using an MOI of 2.5. Positive transduced cells were selected with puromycin (1 µg/ml) for 48 h. SOX10 knockdown was evaluated 4 days after viral transduction. Inducible SOX10 knockdown cells were used in RNA-seq, ChIP-seq (SOX10, BRD4 and 6 histone marks) and ATAC-seq experiments. Inducible SOX10 knockdown cells were established by infecting LN229 cells with pLKO-Tet-On non-targeting (nt) shRNA and pLKO-Tet-On SOX10 shRNA (TRCN0000018988) lentiviral particles and puromycin selection (1 µg/ml) for 7 days. shRNA expression was induced by adding 1 µg/ml doxycycline to the medium for at least 7 days. Cells were cultured in DMEM containing 1 g/l glucose (D5921, Sigma) supplemented with 10% tetracycline-free fetal bovine serum (Clontech), 1% penicillin and streptomycin (P/S) mix and glutamine (0.5 mM). SOX10 knockdown in ZH487 cells were carried out with constitutive shRNA lentivirus infection system (shSOX10-1, 2 and 3) (see Supplementary Table 3 for shRNA sequences).

### RNA isolation, cDNA synthesis and qRT-PCR

Total RNA was isolated using the RNeasy Micro kit (Qiagen) according to the manufacturer’s protocol; 1000 ng was reverse transcribed using random hexamer primers and QuantiTect Rev. Transcription Kit (Qiagen) according to manufacturer’s instructions. Each cDNA sample was analysed in technical triplicate with the Applied Biosystems Prism 7900HT Fast Real-Time PCR System and Absolute SYBR Green ROX Mix (ABgene). The relative amount of specific mRNA was normalised to levels of ARF1 and DCTN2 mRNA. Expression levels were calculated according to the Δ*C*_t_ method. Primer sequences are given in Supplementary Table 2.

### ChIP-qPCR

For the LN229 BRD4 ChIP-PCR experiments, 10 million cells per condition (doxycycline inducible system, control vs. SOX10-KD, DMSO treated vs. JQ1 (500 nM, 6 h)) were cross-linked with 1% methanol-free formaldehyde for 10 min and quenched with 0.125 M glycine. Chromatin was isolated by adding cell lysis buffer (50 mM HEPES pH 7.9,140 mM NaCl,1 mM EDTA,10% glycerol, 0.5% NP-40, 0.25%Triton-100) with protease inhibitor cocktail (Roche, Cat#11836170001). Collected chromatin was sheared via sonication in low SDS shearing buffer (0.1% SDS; 1 mM EDTA;10 mM Tris, pH 8.1) to an average length of 300–500 bp with Covaris S2 system under the conditions indicated (Covaris MicroTube; duty cycle 5%; intensity 4; cycle per burst 200, sonification time 5 min). Input genomic DNA was prepared from collected chromatin by treatment with RNase, proteinase K and de-crosslinking under heat, and then purified with the QIAquick PCR purification kit (Cat#28106, Qiagen). Sonicated chromatin DNA buffer was diluted by adding 10% Triton X-100 and 5 M NaCl (final concentrations of IP buffer: 0.1% SDS; 1 mM EDTA; 10 mM Tris, pH 8.1; 1% Triton-100; 150 mM NaCl). The chromatin was pre-cleared with magnetic protein A/G beads (Cat #CS204457, Millipore), and the samples subjected to immunoprecipitation with 10 µg antibody against BRD4 with overnight incubation in the 4 °C cold room with rotation. The next day, 20 µl fully resuspended Magnetic Protein A/G Beads was added to each sample and incubated in the cold room with rotation for 2 h. Chromatin DNA was purified from the beads by sequential washing with Low Salt Wash Buffer (0.1% SDS; 1% Triton-100; 2 mM EDTA; 20 mM Hepes-KOH, pH 7.9;150 mM NaCl), High Salt Wash Buffer (0.1%SDS; 1% Trtiton X-100; 2 mM EDTA; 20 mM Hepes-KOH, pH7.9; 500 mM NaCl), LiCl Wash Buffer (100 mM Tris-HCl, pH 7.5; 0.5 M LiCl; 1%NP-40; 1%Sodium Deoxycholate) and TE buffer (10 mM Tris-HCl, pH 8.0; 0.1 mM EDTA). Then the magnetic beads containing DNA was eluted with the Elution buffer (10 mM Tris-HCl, pH 8.0; 0.1 mM EDTA; 1% SDS) and de-crosslinking with RNase/proteinase K treatment under heat (65 °C) for 2 h. DNA was then purified with QIAquick PCR purification column. The purified DNA was ready for qRT-PCR analysis. The ChIP primers used are listed in Supplementary Table 2. The relative binding of the investigated proteins to the gene of interest was calculated from qPCR data by calculating the percentage of recovery from the ChIP to the initial input.

### Western blotting

Cells were washed with PBS and were lysed in modified RIPA lysis buffer (0.5% SDS) supplemented with protease inhibitors cocktails and phosphatase inhibitor phosSTOP. The cells were sheared and clear supernatant was collected for protein concentration measurement with the BCA assay. The protein lysates were diluted to 0.5 µg/µl with NuPAGE™ LDS Sample Buffer and reducing agent and boiled at 95 °C for 5 min. A total of 5 µg of protein samples were loaded and resolved on 4–12% Bis-Tris Protein gels according to manufacturer’s instructions. After SDS-PAGE, the proteins were then transferred onto transfer buffer pre-wetted PVDF membrane. Membranes were blocked in 5% skimmed milk or BSA in TBS-T at room temperature for 1 h with gentle shaking. The membranes were incubated with anti-SOX10 (Cat#sc-17342, Santa Cruz, 1:1000 dilution), anti-BRD4 (Cat#A301-985A100, Bethyl Lab., 1:2000 dilution) or anti-alpha-Tubulin (Cat#T9026, Sigma, 1:5000 dilution) in 5% skimmed milk TBS-T at 4 °C overnight with gentle shaking. The membranes were washed with TBS-T for 10 min and repeated three times. Corresponding horseradish peroxidase (HRP) conjugated secondary antibodies (Anti-mouse IgG, Cell Signalling Technology, Cat#7076, 1: 5000; anti-rabbit IgG, Cell Signalling Technology Cat#7074, 1:5000; anti-goat IgG, Santa Cruz sc-2354, 1:5000) were added and incubated for 1 h with gentle agitation at room temperature. The membranes were washed again with TBS-T three times for a total of 30 min before addition of ECL reagents or ECL plus reagents. Signals were subsequently detected by light-sensitive film. Alpha-tubulin was used as loading control. Uncropped images of the western blots are provided in Supplementary Fig. 7.

### Co-immunoprecipitation

Here, 10 million LN229 cells per condition were harvested and washed with PBS. The cells were lysed with Pierce IP lysis buffer (Cat#87787, Life Technologies) supplemented with protease inhibitor cocktail (Cat#11836170001, Roche). Samples were incubated on ice for 30 min with intermittent vertexing. After centrifugation, the supernatant was collected and pre-cleared with Protein G Dynabeads (Cat#10003D, Life Technologies). Protein concentration was determined and Input sample was collected (500 µg each); 10 µg SOX10 antibody (Cat#155279, Abcam) per condition was used to immunoprecipitate protein. Protein G beads were added to the lysate and subjected to overnight incubation, with rotation, in a cold room. The supernatant was removed, isolated beads were washed with TBST three times and resuspended in 20 µl 2× loading buffer (Cat#NP0007, Abcam). Samples were incubated at 70 °C for 10 min and the supernatant was collected via magnetic separation. A further 10 µl 2× loading buffer was added to the beads, and the beads were incubated at 95 °C for 5 min. The supernatant was collected and combined with the previous eluate. This combined eluate was then resolved using SDS-PAGE and the proteins visualised via Western Blot.

### In vitro invasion assay

Here, 200,000 ZH487 cells were seeded into each well of a 6-well plate. Cells were transduced with shRNA targeting SOX10 and NT control, expanded for 4 days, then collected, counted and seeded (50,000 per well) for 36 h in the Neurobasal growth medium (without B27/EGF/FGF) in Biocoat™ Matrigel invasion chambers (8 µm pores, Cat# 354480, BD Bioscience, Bedford, MA). Invasion was then induced by incubation with full growth medium supplemented with B27/EGF/FGF in the lower chamber. Non-invading cells were removed and the remaining cells fixed and stained with haematoxylin. Images were taken with a light microscope (Zeiss, Germany) at 100× magnification.

### Ex vivo brain slice invasion assay

The assay was performed as described^35^. Briefly, a 6–8 week old C57Bl/6 N mouse was euthanized, the brain was isolated and the cerebellum removed with a scalpel. The brain was cut in 350 μm thick coronal slices with a vibratome (Leica VT1200 S). The slices were cultivated on top of a filter (Cat#PICM03050, Millipore) in a 6-well plate with a medium composed of: MEM (Cat# M2279, Sigma), 25% heat-inactivated horse serum (Cat# 26050070, Life Technologies), 25 mM HEPES (Cat#H0887-100 ml, Sigma), 1 mM L-glutamine (Cat#G7513, Sigma), 5 mg/ml glucose (Sigma Cat#G8769), 100 U/ml penicillin/streptomycin (Cat#P4333, Sigma). Control (shNT) and inducible SOX10-KD LN229 cells were treated with Dox for at least 7 days. LN229, control and SOX10-KD, glioma cells cultivated in medium (DMEM, Cat# D5671, Sigma; 10% FBS, Cat#F7524, Sigma; 2 mM L-glutamine) were trypsinized and counted. 1 × 10^6^ cells/ml PBS were incubated with 5 μl lipophilic dye DiD (1 mg/ml in DMSO, Cat#60014, Biotium) for 30 min at 37 °C. After two washing steps 500 cells/well for control LN229 and 1200 cells/well for LN229 SOX10-KD were seeded in a flat-bottom 96-well plate coated with 50 μl low melt agarose (Cat# M3049.0010, Genaxxon; 1% in PBS). After 3 days spheroids were collected and manually implanted in the brain slices using a blunt Hamilton syringe (701 N; 10 μl; 26 s/51/3) and a binocular microscope. Three days after implantation the brain slices were fixed with 4% PFA and cleared according to the SeeDB protocol^82^.

### Immunohistochemistry of mouse tumours

For immunohistochemistry experiments, paraffin embedded (4 μm) tumour tissues were first subjected to deparaffinization and citric acid-based antigen retrieval was performed following standard protocols. Sections were either stained with hematoxylin and eosin (H&E) or subjected to immunohistochemistry Iba1 (Cat#019-19741, Wako, 1:2000). Immunohistochemistry images were obtained with light microscope (Zeiss, Germany) with 20× and 100x objectives. Iba1 staining quantification was performed with ImageJ (NIH) using random areas from the tumour core region. For immunofluorescence staining, sections were stained for GFP (Cat#13970, abcam, 1:500 and anti-Chicken IgY (H + L) secondary antibody, Alexa Fluor 488, Invitrogen A-11039, 1:1000) and immunofluorescence images of GFP were captured using Leica TCS SP8 confocal microscope with 20× objective.

### Quantification and statistical analysis

Statistical analysis was performed using R and Students *t*-test. Kaplan-Meier analysis was performed to estimate the survival time of different GBM subgroups and a log rank test was used to test for differences of more than one survival curve. Details of the statistical tests applied are stated in the figure legends and the main text. In all Tukey-style boxplots, the box corresponds to the 25^th^, 50^th^/median and 75^th^ percentiles and the whiskers denote 1.5× the IQR from the median. Outliers beyond 1.5× IQR are shown as points. Other boxplots indicate mean values ± standard deviation.

### Reporting summary

Further information on research design is available in the Nature Research Reporting Summary linked to this article.

## Supplementary information

Supplementary Information

Description of Additional Supplementary Files

Supplementary Data 1

Supplementary Data 2

Supplementary Data 3

Supplementary Data 4

Supplementary Data 5

Supplementary Data 6

Supplementary Data 7

Supplementary Data 8

Supplementary Data 9

Supplementary Data 10

Supplementary Data 11

Supplementary Data 12

Supplementary Data 13

Supplementary Data 14

Reporting Summary

## Data Availability

The processed DNA-methylation array, WGBS, RNA-Seq, ChIP-Seq, ATAC-Seq and whole genome sequencing data used in this study is available at the gene expression omnibus (GEO) database under accession code GSE121723). The raw WGBS, RNA-Seq, ChIP-Seq, ATAC-Seq and whole genome sequencing data data are deposited in the European Genome-Phenome Archive (EGA) database under accession code EGAS00001003953. The data is available under restricted access, which can be obtained by contacting the HIPO data access committee (katja.beck@nct-heidelberg.de). The gene expression datasets used for the RTN analysis were obtained from E-MTAB-3073 (https://www.ebi.ac.uk/arrayexpress/experiments/E-MTAB-3073/), and GSE4290, GSE7696, GSE16011 and GSE43378 (https://www.ncbi.nlm.nih.gov/geo/query). The remaining data are available within the Article, Supplementary Information or available from the authors upon request.

## Code availability

Custom analysis scripts are available at https://github.com/dkfz-b060/gb_code.
