# Supplementary material for: Glioblastoma epigenome profiling identifies SOX10 as a master regulator of molecular tumour subtype
Source: Nat Commun. 2020 Dec 18;11:6434. doi: 10.1038/s41467-020-20225-w (PMC7749178; doi:10.1038/s41467-020-20225-w)
Supplement: Supplementary file 1 — Supplementary Information [file 41467_2020_20225_MOESM1_ESM.pdf]

Supplementary Information

Glioblastoma epigenome profiling identifies SOX10 as a master regulator of molecular tumour subtype

Yonghe Wu[1,2*+], Michael Fletcher[1*], Zuguang Gu[2,3*], Qi Wang[3], Barbara Costa[4], Anna Bertoni[1], Ka-Hou Man[1], Magdalena Schlotter[1], Jörg Felsberg[5,6], Jasmin Mangei[1], Martje Barbus[1], Ann-Christin Gaupel[1], Wei Wang[1], Tobias Weiss[7], Roland Eils[2,3,], Michael Weller[7], Haikun Liu[8], Guido Reifenberger[5,6], Andrey Korshunov[9,10], Peter Angel[4], Peter Lichter[1,2,6], Carl Herrmann[11]§, Bernhard Radlwimmer[12]§

Affiliations
[1] Division of Molecular Genetics, German Cancer Research Center (DKFZ), Im Neuenheimer Feld 280, 69120 Heidelberg, Germany
[2] Heidelberg Center for Personalized Oncology (DKFZ-HIPO), Im Neuenheimer Feld 280, 69120 Heidelberg, Germany
[3] Division of Theoretical Bioinformatics, German Cancer Research Center (DKFZ), Im Neuenheimer Feld 280, 69120 Heidelberg, Germany
[4] Division of Signal Transduction and Growth Control, DKFZ/ZMBH Alliance, Im Neuenheimer Feld 280, 69120 Heidelberg, Germany.
[5] Institute of Neuropathology, Medical Faculty, Heinrich Heine University, Moorenstr. 5, 40225 Düsseldorf, Germany
[6] German Cancer Consortium (DKTK), partner site Essen/Düsseldorf, German Cancer Research Center (DKFZ), Im Neuenheimer Feld 280, 69120 Heidelberg, Germany
[7] Department of Neurology and Brain Tumor Center, University Hospital Zurich, Frauenklinikstrasse 26, CH-8091 Zurich, Switzerland
[8] Division of Molecular Neurogenetics, DKFZ-ZMBH Alliance, Im Neuenheimer Feld 280, 69120 Heidelberg, Germany.
[9] Department of Neuropathology, University of Heidelberg, Im Neuenheimer Feld 220, 69120 Heidelberg, Germany
[10] Clinical Cooperation Unit Neuropathology, German Cancer Research Center (DKFZ), Im Neuenheimer Feld 220-221, 69120 Heidelberg, Germany
[11] Health Data Science Unit, Medical Faculty Heidelberg, Im Neuenheimer Feld 267, 69120 Heidelberg, Germany. carl.herrmann@bioquant.uni-heidelberg.de
[12] Division of Molecular Genetics, German Cancer Research Center (DKFZ), Im Neuenheimer Feld 280, 69120 Heidelberg, Germany. b.radlwimmer@dkfz.de

* These authors contributed equally.
§These authors jointly supervised this work.

Supplementary Fig. 1. Sample subtyping and genome-wide methylation dynamics in glioblastoma. **a** Glioblastoma samples (*n* = 60) clustered using methylation array probes (*n* = 7386) from Sturm et al., 2012. **b** Consensus subtyping based CpG Island (CGI) methylation and non-CGI methylation applied with hierarchical partitioning. Results are presented as follows: the inner circle corresponds to the methylation array subtyping as depicted in panel (a). The outer circle represents the cluster that the sample is assigned to in that data type, colored by the cluster identity. We found that CGI methylation cannot distinguish the MES and RTK I subtypes, while for non-CGI methylation the MES subtype splits into two subgroups, of which one is closer to the RTK II samples. These results illustrate that the methylation dynamics of GB subtypes vary depending on the features analysed. **c** Density heatmaps showing per-sample DNA methylation (*y*-axis, WGBS beta) genome-wide, in TSSs, exons, introns, and in intergenic regions. Heatmap colors correspond to density of the methylation distribution in each sample (column). Samples are labelled with their subtype. Below the heatmaps, multidimensional scaling (MDS) plots for sample methylation corresponding to (A) As observed in the methylation consensus subtyping in panel (b), the separation of subtypes is dependent on the methylation feature examined.

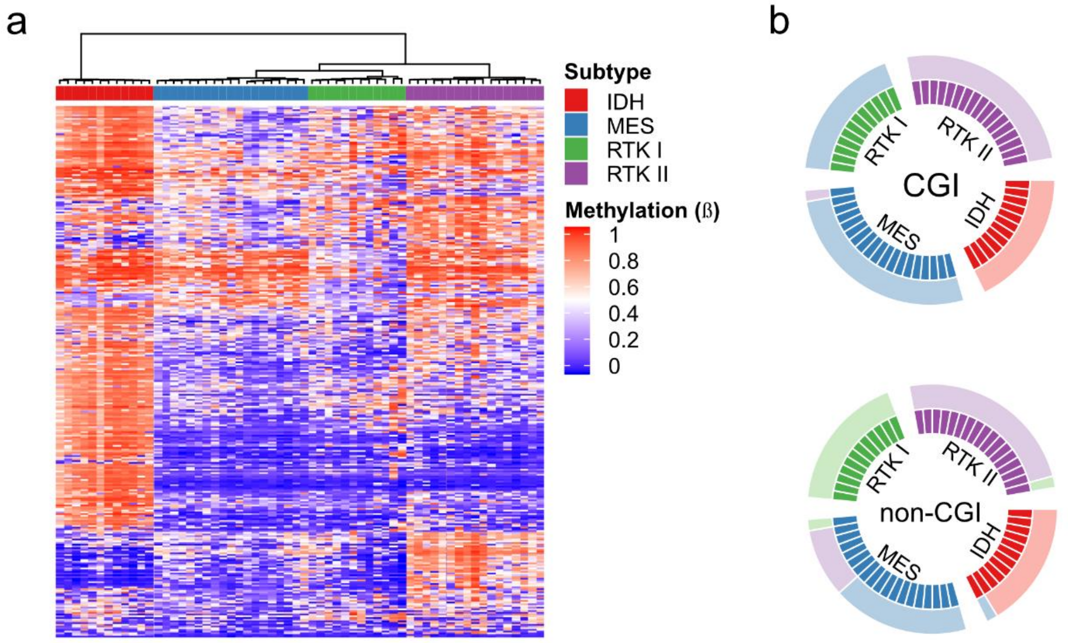

Hierarchical clustering of n=60 GBM samples (methylation array data). 7386 probes from Sturm et al. (2012) common to 450k and EPIC platforms; Euclidean distance, hclust method: ward.D.

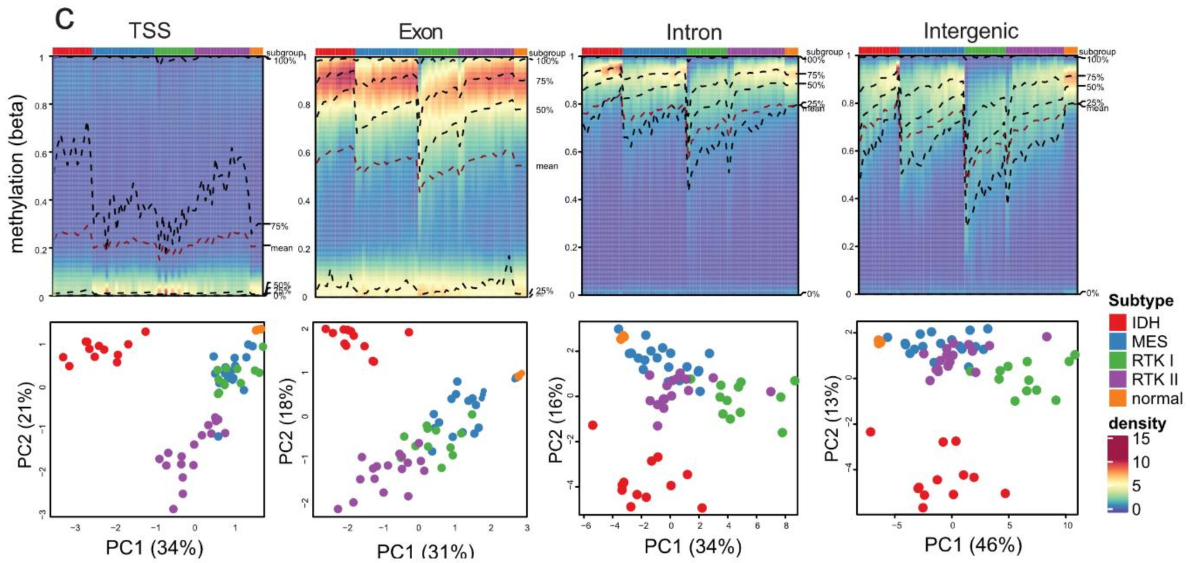

Supplementary Fig. 2. limma analysis of subtype gene signatures. **a** Venn diagram showing overlap of the limma-defined subtype gene signatures. For each subtype, gene expression was compared to the other 3 subtypes weighted equally (example: IDH vs (MES+RTK I+RTK II)/3). Genes with adj. *P*-value < 0.001 were deemed significant. **b** Multi-dimensional scaling (MDS) plot of all genes present in the 4 subtype gene signatures in all subtyped glioblastomas (*n* = 60) using limma-voom log-cpm values. Samples are coloured by their subtype. **c** Examples of subtype genes. From left: FREM3, IDH; CXCL5, MES; ERBB3, RTK I; MEOX2, RTK II. log-cpm values from limma-voom are plotted, grouped by subtype, as Tukey boxplots and violin plots. limma 2-sided p-values (corrected using the Benjamini-Hochberg method) are displayed. IDH, *n* = 12; MES, *n* = 19; RTK I, *n* = 12; RTK II, *n* = 17 samples. **d** Cahoy mouse brain cell signature ssGSEA enrichment and ESTIMATE scores for each subtype, shown as Tukey boxplots and violin plots. IDH, *n* = 12; MES, *n* = 19; RTK I, *n* = 12; RTK II, *n* = 17 samples. **e** Gene Ontology term annotation of subtype gene expression (significant terms at adj. *P*-value < 0.1 (two-sided) are shown; correction for multiple testing using the Benjamini-Hochberg method). Up-regulated genes for each subtype (logFC > 0, adj. *P*-value < 0.05) were tested for Gene Ontology enrichment. The number of tested genes for each subtype is indicated.

Supplementary Fig. 2

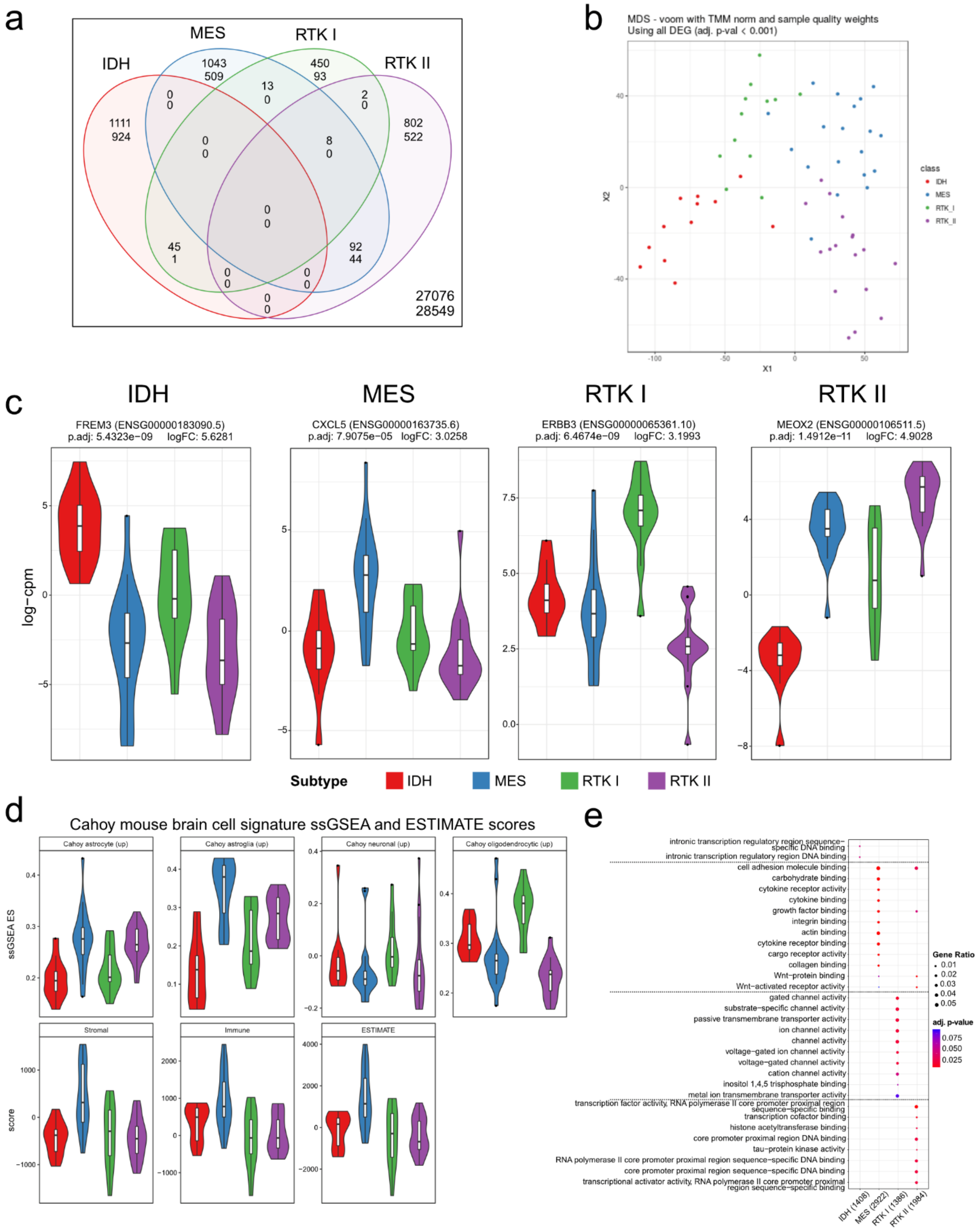

Supplementary Fig. 3. RTN analysis. **a** MDS plots showing the result of ComBat batch effect removal on the samples in cohort B. Each point corresponds to a sample, coloured by the source study. **b** Comparison of networks A and B using TF subtype activation scores. For TFs common to both networks (*n* = 506), activity was defined as the 2-tail GSEA differential enrichment score (dES) for each TF regulon for each subtype signature. Common subtype MRs (adjusted *P*-value < 0.01 with the same direction of activation in both networks) are coloured, and the Spearman's rank correlation is displayed. **c** Overlap of significant MRs identified in networks A and B. The overlap (*n* = 126) is larger than the number of common subtype MRs (*n* = 117) as the subtype is not taken into account. **d** Activity of common subtype MRs (*n* = 117) across all subtypes. A heatmap of the TF activity (two-tailed dES-test, red activation; blue repression) is shown, with TFs (rows) clustered by the dES scores. Subtype significant MRs (adj. *P*-value < 0.01; correction for multiple testing using the Benjamini-Hochberg method) are labelled with asterisks. We identify previously reported GB MRs (FOSL1/2, CEBP) and CNS developmental TFs (HES1, JUND, GLI2, POU3F2, SOX9, TCF3, NFIC, MAFB, BNC2, ASCL1, MYT1, MYT1L, SOX10). The two-tailed dES-test is similar to the GSEA permutation test (n=1000 permutations) using the computed enrichment score.

Supplementary Fig. 3

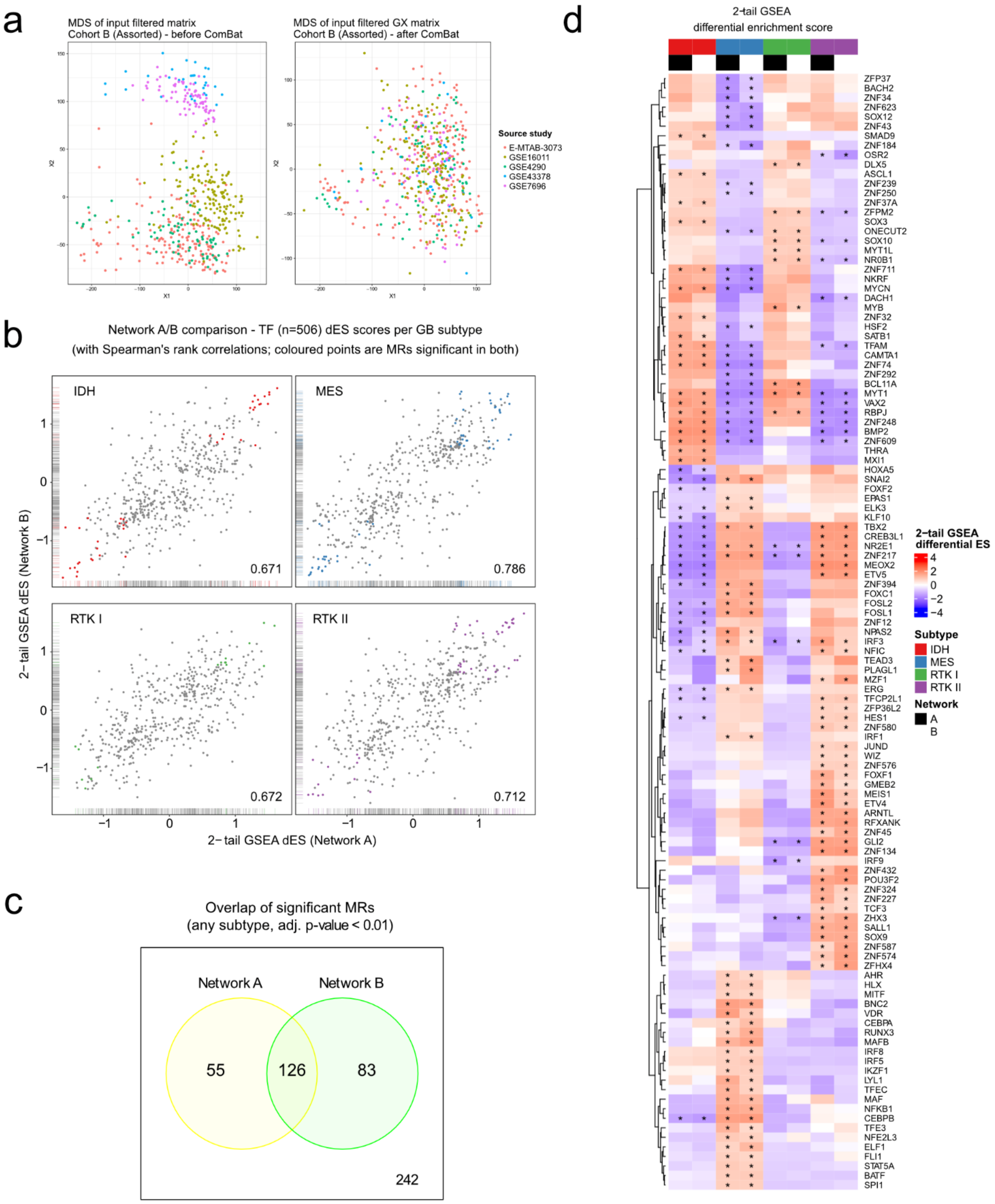

Supplementary Fig. 4. Cell line model selection. **a** SOX10 locus DNA methylation (beta) assayed by the 450k/EPIC DNA methylation arrays in GB cell lines (top) and the MES and RTK I subtype averages in this study's tumours (bottom). The distance from the assayed CpG to the SOX10 TSS is indicated above the heatmap. **b** SOX10 gene expression (Affymetrix microarray intensities) in GB cell line models. **c** Genome browser visualisation of the SOX10 locus. Histone modifications (H3K27ac, H3K4me1, H3K4me3) and the ChromHMM annotation from a single representative RTK I tumour and LN229 cells are shown, along with SOX10 binding (ChIP-seq), chromatin accessibility (ATAC-seq) and RNA-seq in LN229 and ZH487 cells. **d** Venn diagram of the overlap of SOX10 binding sites identified using ChIP-seq in the ZH487 (left) and LN229 (right) cell line models. The most significant result of *de novo* motif finding with HOMER, annotated as SOX10, for these common binding sites is displayed. Enrichment P-value for SOX10 was computed based on a binomial test comparing the frequency of the motif in foreground vs. background sequences.

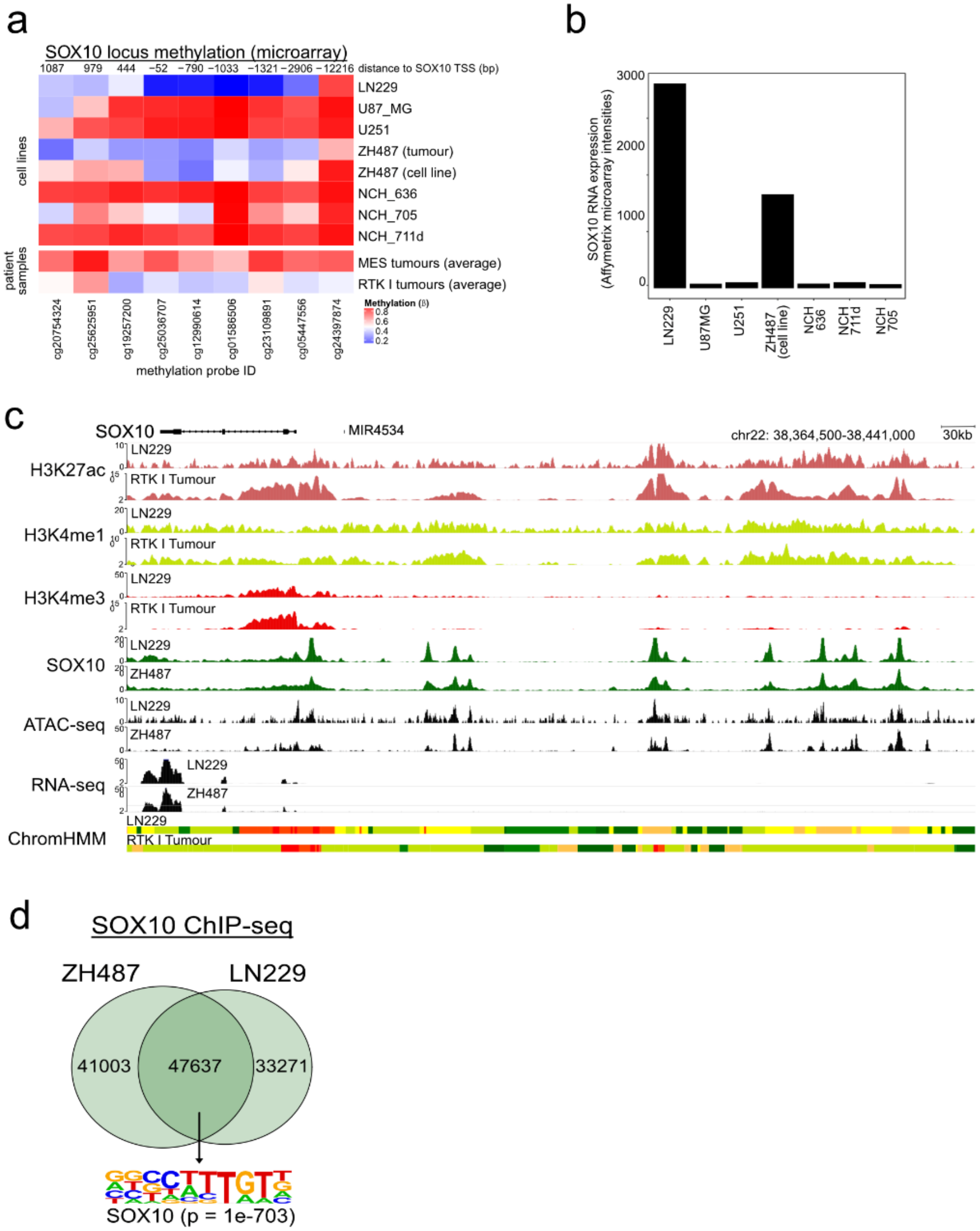
a
SOX10 locus methylation (microarray)
1087 979 444 −52 −790 −1033 −1321 −2906 −12216 distance to SOX10 TSS (bp)
LN229
U87_MG
U251
ZH487 (tumour)
ZH487 (cell line)
NCH_636
NCH_705
NCH_711d
MES tumours (average)
RTK I tumours (average)
cell lines
patient samples
cg20754324
cg25625951
cg19257200
cg25036707
cg12990614
cg01586506
cg23109891
cg05447556
cg24397874
methylation probe ID
Methylation (β)
0.8
0.6
0.4
0.2
b
SOX10 RNA expression (Affymetrix microarray intensities)
3000
2000
1000
0
LN229
U87MG
U251
ZH487 (cell line)
NCH 636
NCH 711d
NCH 705
c
SOX10
MIR4534
chr22: 38,364,500-38,441,000
30kb
H3K27ac
H3K4me1
H3K4me3
SOX10
ATAC-seq
RNA-seq
ChromHMM
LN229
RTK I Tumour
ZH487
d
SOX10 ChIP-seq
ZH487
LN229
41003
47637
33271
SOX10 (p = 1e-703)

Supplementary Fig. 5. SOX10 repression causes a PN-MES transcriptomic switch. **a** Western blot showing efficient repression of SOX10 protein expression in ZH487, using shRNA (shSOX10), and LN229, using CRISPRi (sgSOX10); based on three independent experiments, each. Alpha-tubulin loading control is also displayed. Molecular weights (kDa) measured by standards are indicated on the left. **b** GSEA plots showing enrichment of a mesenchymal gene signature and concurrent depletion of a proneural gene signature in SOX10 KD relative to control ZH487cells. GSEA-calculated statistics for gene set enrichment are shown. Top row: limma signatures; Bottom row: Wang signatures. *P*-values (control/RTK I: 0.018; control/PN_: 0.074; KD/MES: < 0.001; KD/MES-Wang: < 0.001) and FDR values are computed empirically using a permutation test (n=1000 permutations) based on the enrichment score. **c** Master Regulator activity heatmap (VIPER NES) for the RTK I and MES CRC MRs, in the LN229 and ZH487 cell line models. Average expression profiles for each condition (for LN229, n = 1 replicate; for ZH487, n = 2 replicates) were used in a VIPER analysis using RTN network A. Each column corresponds to a cell line condition, and is annotated with the cell line and treatment (top) and VIPER-inferred activity of SOX10 (bottom). *: two-tailed t-test, *P*-value < 0.05. **d** Trans-well invasion assay of ZH487 cells without (top, control) and with SOX10 repression (bottom). Cell numbers were quantified for n =10 wells per condition in one experiment; scale bar: 50 µm. **e** *Ex vivo* organotypic brain slice invasion assay. LN229 cells with and without SOX10 repression (shNT+Dox vs. shSOX10+Dox) were seeded into freshly harvested murine brain slices (350 µm) and left for 3 days. Representative, inverted colour images and bar plots of mean cell-spike length (pixels) of one experiment are shown; on average 15 spikes were examined per cell; control, n = 21 cells; SOX10 KD, n = 15 cells; ; scale bar: 100 µm. Mean values are plotted in (d) and (e); whiskers indicate the standard deviation.

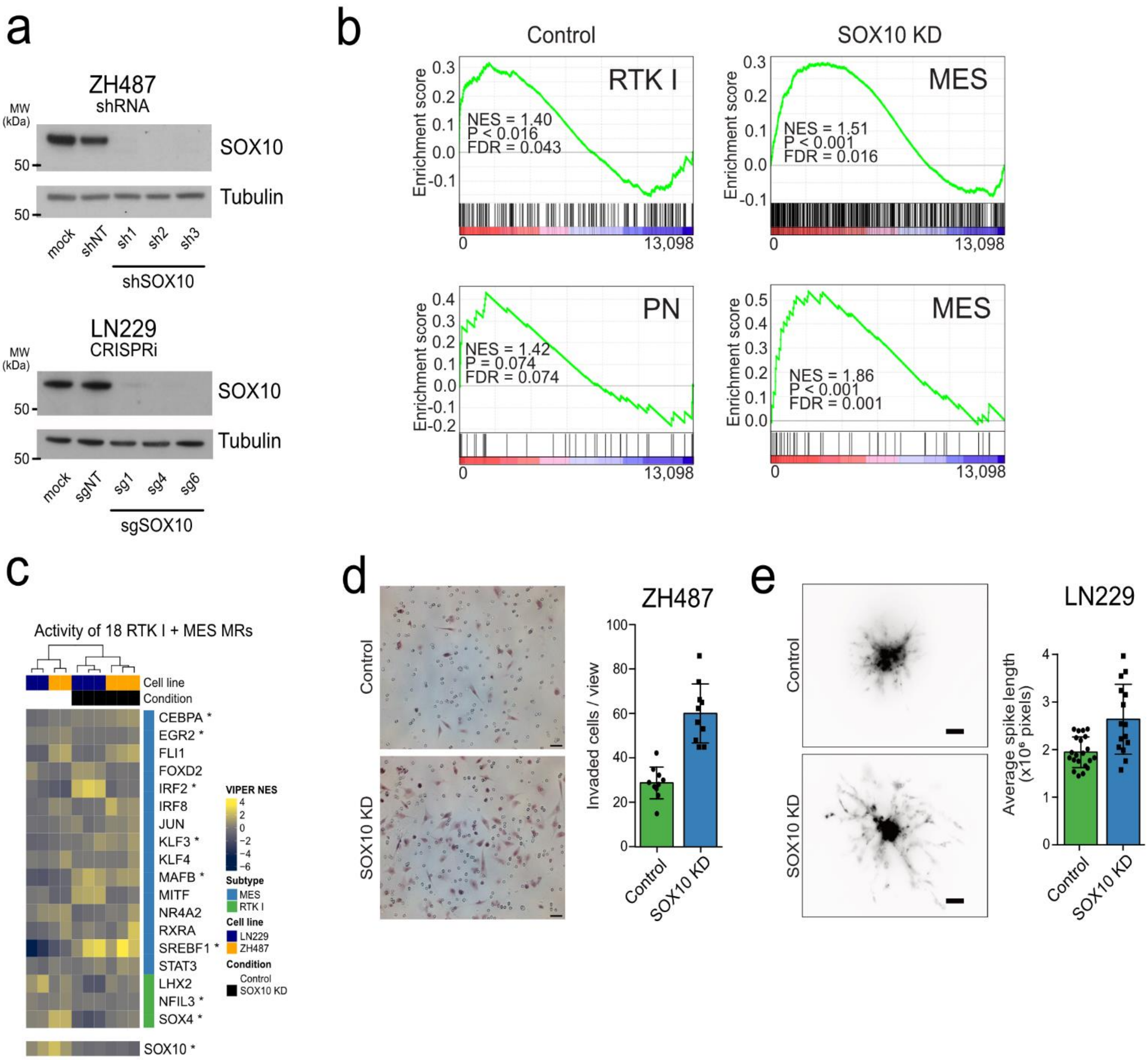
a
ZH487
shRNA
MW (kDa)
SOX10
Tubulin
50
mock
shNT
sh1
sh2
sh3
shSOX10
LN229
CRISPRi
SOX10
Tubulin
mock
sgNT
sg1
sg4
sg6
sgSOX10
b
Control
SOX10 KD
RTK I
NES = 1.40
P < 0.016
FDR = 0.043
MES
NES = 1.51
P < 0.001
FDR = 0.016
PN
NES = 1.42
P = 0.074
FDR = 0.074
MES
NES = 1.86
P < 0.001
FDR = 0.001
Enrichment score
0
13,098
c
Activity of 18 RTK I + MES MRs
Cell line
Condition
CEBPA *
EGR2 *
FLI1
FOXD2
IRF2 *
IRF8
JUN
KLF3 *
KLF4
MAFB *
MITF
NR4A2
RXRA
SREBF1 *
STAT3
LHX2
NFIL3 *
SOX4 *
SOX10 *
VIPER NES
Subtype
MES
RTK I
Cell line
LN229
ZH487
Condition
Control
SOX10 KD
d
Control
SOX10 KD
ZH487
Invaded cells / view
Control
SOX10 KD
e
Control
SOX10 KD
LN229
Average spike length (x10⁶ pixels)
Control
SOX10 KD

Supplementary Fig. 6. ZH487 genomics experiments. **a** EnrichedHeatmap visualisation of genome regions with differential chromosome accessibility in control and SOX10 KD ZH487 cells, as identified by ATAC-seq analysis. SES-normalised signals of SOX10 ChIP-seq, ATAC-seq, and BRD4 ChIP-seq are displayed. Signal intensity is shown in the blue-red heatmaps, where each row shows a single ATAC peak, as indicated by the vertical dashed lines, and 1kbp further 5' and 3'. The line plots at the top of each heatmap display the mean signal intensity across all the regions in that category (control: green; SOX 10 KD: blue). **b** Volcano plots of de novo motif finding with HOMER from the differentially bound ATAC-seq peaks in the ZH487 cells. The significantly enriched motifs are labelled. **c** Visualisation of ChIP-seq signal at SOX10 peaks in the ZH487 model. Per-factor scaled, SES-normalised signal is visualised for each SOX10 peak, extended for a further 1kbp on either side. **d** The epigenomic landscape of RUNX2 in the LN229 cell line in control (NT) and shSOX10 conditions. The tracks display (from top to bottom): RNA-seq; ATAC-seq; ChIP-seq signal for BRD4, H3K27ac and H3K4me1; and the ChromHMM annotation. Regions of interest are denoted with boxes (Prom, promoter; Enh. 1-3, candidate enhancer regions). **e** RUNX2 RNA expression levels in LN229 and ZH487 cells measured by RT-qPCR following JQ1 treatment (500nm, 6h) and SOX10 repression. **f** JQ1 treatment blocks BRD4 recruitment to RUNX2 enhancer regions in the LN229 model. The four open chromatin regions labelled in (H) (Prom, Enh. 1-3) were tested for BRD4 binding using ChIP-qPCR following SOX10 repression and JQ1 treatment (500nm, 6h). In (e) and (f) the mean values of 3 technical replicates of one experiment are shown.

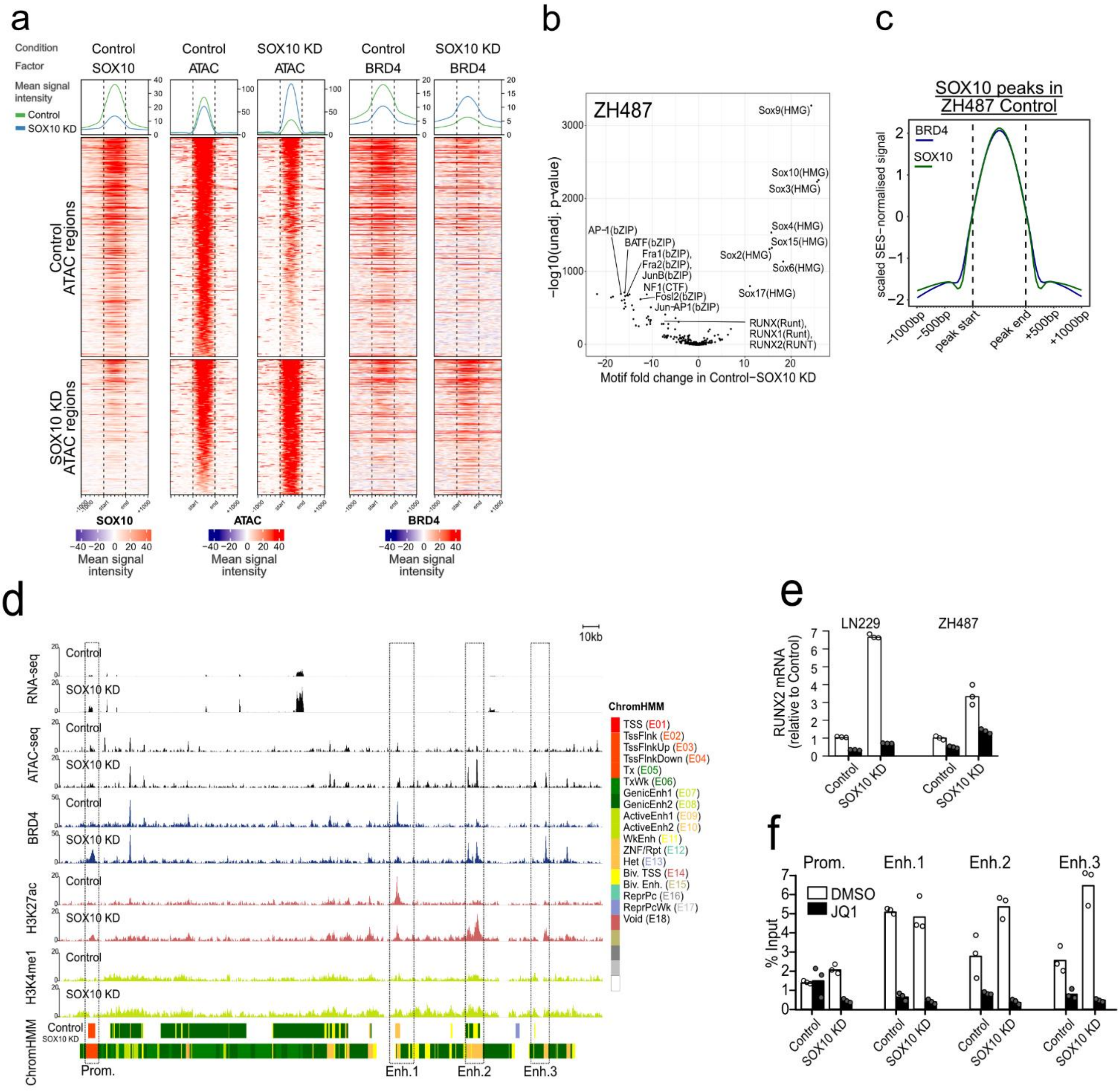
a
b
c
d
e
f
ZH487
SOX10 peaks in ZH487 Control
Motif fold change in Control−SOX10 KD
−log10(unadj. p-value)
scaled SES−normalised signal
RUNX2 mRNA (relative to Control)
% Input
LN229
ZH487
Prom.
Enh.1
Enh.2
Enh.3
DMSO
JQ1
ChromHMM
10kb

Supplementary Fig. 7. Uncropped Western blots. **a** BRD4/SOX10 co-IP in the cell line LN229 (Figure 5h). **b** SOX10 knockdown in the cell lines LN229 and ZH487 (Supplementary Fig. 5a).

a

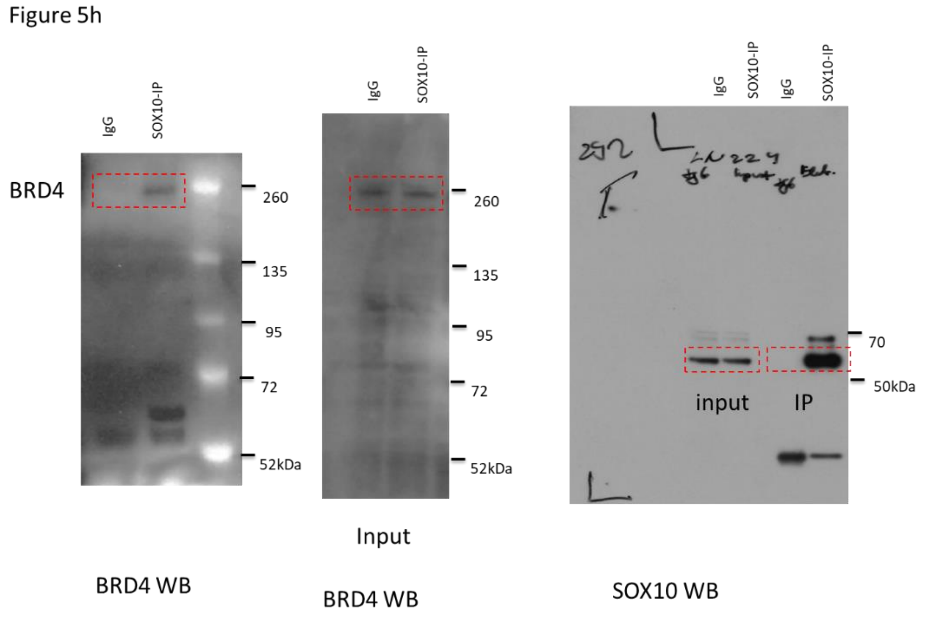

b

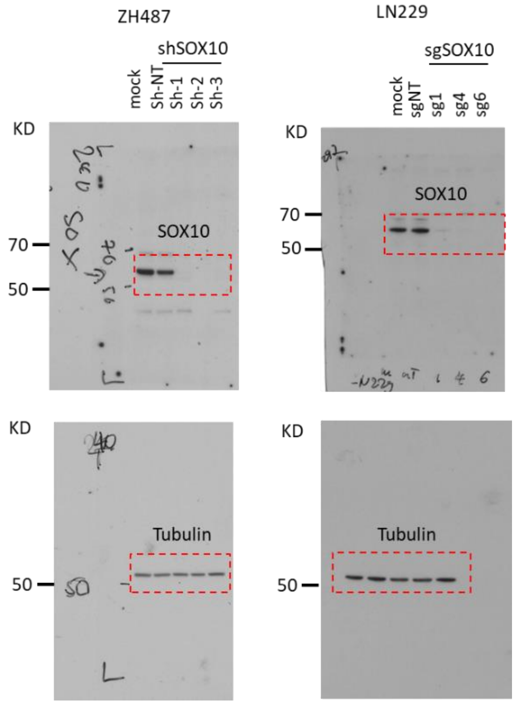

Supplementary Table 1

REAGENT or RESOURCE	SOURCE	IDENTIFIER
Antibodies		
Goat anti-human SOX10 (Western blot)	Santa Cruz	Cat#sc-17342
Rabbit anti-human SOX10 (ChIP-seq, Co-IP)	Abcam	Cat#ab155279, lot GR113617-51
Rabbit anti-Iba1 antibody (IHC)	Wako	Cat#019-19741
Rabbit polyclonal BRD4 Ab (ChIP-seq, WB and ChIP-qPCR)	Bethyl Lab.	Cat#A301-985A100
H3K27Ac (GB patient ChIP-seq)	Active Motif	Cat#AM#39133, lot 5
H3K4me1(GB patient ChIP-seq)	Active Motif	Cat#AM#38297, lot 1
H3K4me3 (GB patient ChIP-seq)	Active Motif	Cat#AM#39159, lot 2
H3K9me3 (GB patient ChIP-seq)	Active Motif	Cat#AM#39161, lot 3
H3K27me3 (GB patient ChIP-seq)	Millipore	Cat##07-449, lot 2382150
H3K36me3 (GB patient ChIP-seq)	Active Motif	Cat#AM#61101, lot 7
Rabbit polyclonal to Histone H3 (cell line ChIP-seq)	Abcam	Cat#ab1791
Rabbit polyclonal to Histone H3K4me1 (cell line ChIP-seq)	Abcam	Cat#ab8895
Rabbit polyclonal to Histone H3K4me3 (cell line ChIP-seq)	Abcam	Cat#ab8580
Rabbit polyclonal to Histone H3K9me3 (cell line ChIP-seq)	Abcam	Cat#ab8898
Rabbit polyclonal to Histone H3K27ac (cell line ChIP-seq)	Abcam	Cat#ab4729
Rabbit polyclonal to Histone H3K36me3 (cell line ChIP-seq)	Abcam	Cat#ab9050
Rabbit polyclonal to Histone H3K27me3 (cell line ChIP-seq)	Abcam	Cat#ab6002
alpha-Tubulin	Sigma	Cat#T-9026
Green Fluorescence Protein (immunofluorescence)	abcam	Cat#13970
Allograft Inflammatory Factor 1 (IBA1; immunofluorescence)	Wako	Cat#019-19741
Bacterial and Virus Strains		
TOP10 Chemically Competent E. coli	Invitrogen	Cat#C404010
Lentivirus (pLKO1, constitutive, packed in HEK293)	Sigma Aldrich	Cat# SHC001
Lentivirus (pLKO-Tet-On, packed in HEK293)	Addgene	Cat#21915
Lentivirus (CRISPRi) pU6-sgRNA EF1Alpha-puro-T2A-BFP	Addgene	Cat#60955
Biological Samples		
Human glioblastoma tissue samples	Moscow, Russia	Burdenko Neurosurgical Institute
Chemicals, Peptides, and Recombinant Proteins		
DMEM medium	Sigma	Cat#D5546
L-glutamine	Sigma	Cat#G7513
Pen-strep	Sigma	Cat#P4333
Fetal bovine serum	Gibco	Cat#10082147
Tet-Free FBS	Clontech	Cat#631106
Neurobasal medium	Invitrogen	Cat#10888-022
Human Recombinant EGF	Gibco	Cat#PHG0311
Human Recombinant FGF	Biomol	Cat#50361
B27 medium	Invitrogen	Cat#12587010
QuantiTect Rev. Transcription Kit (50)	Qiagen	Cat#205311
Puromycin	Gibco	Cat#A1113803

RNase-Free DNase Set (50)	Qiagen	Cat#79254
QIAquick PCR Purification Kit (250)	Qiagen	Cat#28106
QIAprep Spin Miniprep Kit (250)	Qiagen	Cat#27106
QIAGEN Plasmid Maxi Kit (25)	Qiagen	Cat#12163
RNeasy Mini Kit (250)	Qiagen	Cat#74106
Bioanalyzer High Sensitivity DNA Analysis	Agilent	Cat#5067-4626
Qubit™ dsDNA HS Assay Kit	Invitrogen	Cat#Q32851
Qubit™ RNA HS Assay Kit	Invitrogen	Cat# Q32852
DMEM/HAM F12	Biochrom	Cat#F4815
Critical Commercial Assays		
TruSeq Stranded Total RNA LT Sample Prep Kit	Illumina	Cat#RS-122-2201
Paired-End Sample Prep Kit	Illunima	Cat#PE-102-1002
EZ DNA Methylation Kit	Zymo Research	Cat#D5001
Magna ChIP™ A/G Chromatin Immunoprecipitation Kit	Merck-Millipore	Cat#17-10085
Deposited Data		
Raw data (restricted access)	EGA	EGAS00001003230
Processed and pseudo-anonymised data	GEO	GSE121723
Experimental Models: Cell Lines		
Human: LN229	ATCC	Cat# CRL-2611
Human: GB patient-derived ZH487	Zurich University	N/A
Human: HEK-293T	ATCC	Cat#CRL-3216
Mouse: mGB1	DKFZ	Peter Angel's lab
Oligonucleotides		
qPCR Primers (see Supplementary Data 13)	This paper	N/A
shRNA oligoes targeting human and mouse SOX10 (see Supplementary Data 13)	This paper	N/A
CRISPR-Cas9 SOX10 knockdown constructs (see Supplementary Data 13)	This paper	N/A
ChIP-qPCR primers sequence (see Supplementary Data 13)	This paper	N/A
Recombinant DNA		
pLKO.1-puro	Sigma Aldrich	Cat# SHC001
psPAX2 (lentivirus packaging plasmid)	Addgene	Cat#12260
pMD2.G (Lentivirus envelope plasmid)	Addgene	Cat#12259
Software and Algorithms		
R	R-Project	3.4.3, 3.5.1
STAR	Open Source	2.3.0e
htseq-count	Open Source	0.6.0
bwa-mem	Open Source	0.7.8
methylCtools	Open Source	1.0.0
BEDTools	Open Source	2.27.1
MethylSeekR		1.14.0
Snakemake	Open Source	3.13.3

TrimGalore	Open Source	https://github.com/FelixKrueger/ TrimGalore
deepTools2	Open Source	DeepTools (v. 2.3.1); Ref. 10
MACS2	Open Source	2.1.1.2016030 https://github.com/taoliu/MACS
BowTie2	Open Source	v. 2.3.4.39
SICER		Ref. 22
ChromHMM	Open Souce	1.19
ROSE2	Open Source	https://github.com/BradnerLab/ pipeline/
bigWigAverageOverBed	Open Source	v2
CRCmapper	Open Source	version 1.0 (December 2015) Ref. 13
GSEA	Broad Institute	v3.0
HOMER	Open Source	4.9.1
minfi	Bioconductor	1.24.0
conumee	Bioconductor	1.30.0
edgeR	Bioconductor	3.20.1
limma	Bioconductor	3.34.4
GSVA	Bioconductor	1.26.0
ESTIMATE	Bioconductor	1.0.13
clusterProfiler	Bioconductor	3.6.0
cola	Bioconductor	1.0.0
gcrma	Bioconductor	2.50.0
sva	Bioconductor	3.26.0
RTN	Bioconductor	2.3.4
ggplot2	Bioconductor	2.2.1
monocle	Open Source	2.10.1
viper	Bioconductor	1.14.0
ComplexHeatmap	Bioconductor	1.18.1, 1.19.1
Rtsne	CRAN	0.13
ChIPpeakAnno	Bioconductor	3.12.4
EnrichedHeatmap	Bioconductor	1.9.2
TxDb.Hsapiens.UCSC.hg19.knownGene	Bioconductor	3.2.2
DiffBind	Bioconductor	2.6.6
circlize	Bioconductor	0.4.6
Gviz	Bioconductor	1.22.3
epik	Open Source	https://github.com/jokergoo/epik
featureCounts in the Subread suite	Open Source	1.5.3

Supplementary Table 2

qPCR primers		
name	species	primer sequence
SOX10-F	human	CTTTCTTGTGCTGCATACGG
SOX10-R	human	AGCTCAGCAAGACGCTGG
ERBB3-F	human	CAAGTTCCCTTGAGGAGCTG
ERBB3-R	human	CATCTCGTTGCCGATTCATA
Olig2-F	human	GCTCCTCAAATCGCATCCA
Olig2-R	human	AAAGGTCATCGGGCTCTG
CSPG4-F	human	TGGCCTTCACTGTCACTGTCC
CSPG4-R	human	CACTTGCTTCTGGGCCGTCACTCG
CD44-F	human	GACACCATGGACAAGTTTTGG
CD44R	human	AAGCGGCAGGTCATATTCAA
RUNX2-F	human	TGGTTACTGTCATGGCGGGTA
RUNX2-R	human	TCTCAGATCGTTGAACCTTGCTA
FOSL2-F	human	CAGAAATTCCGGGTAGATATGCC
FOSL2-R	human	GGTATGGGTTGGACATGGAGG
SERPINE1-F	human	ACCGCAACGTGGTTTTCTCA
SERPINE1-R	human	TTGAATCCCATAGCTGCTTGAAT
ARF-F	human	GACCACGATCCTCTACAAGC
ARF-R	human	TCCCACACAGTGAAGCTGATG
DCTN2-F	human	CGCCATGGCGACCCTAAAT
DCTN2-R	human	TTGTCAGCTCCTCCGCATCGAA
Sox10-F	mouse	GTGCCAGCAAGAGCAAGCCG
Sox10-R	mouse	CTGCCTTCCCGTTCTTCCGCC
Hprt-F	mouse	CTTCCTCCTCAGACCGCTTTTT
Hprt-R	mouse	ATGTAATCCAGCAGGTCAGCAA
Gapdh-F	mouse	GTGCAGTGCCAGCCTCGTCC
Gapdh-R	mouse	CAGGCGCCCAATACGGCCAA
**ChIP-PCR primers**		
RUNX2-Prom-F	human	CCATCACCTCCATCCTCTTTC
RUNX2-Prom-R	human	AATACGCATCACAACAGCC
RUNX2-enh1-F	human	TTTTTCTGCTGCGAACAATG
RUNX2-enh1-R	human	CCCCAAAGACTTCCAAAACAC
RUNX2-enh2-F	human	CTTTCCATTCCTCTTCTCTTCC
RUNX2-enh2-R	human	CGAAAATAGCTCAGCTCTGAC
RUNX2-enh3-F	human	CCAAAAGAACGACCACAAAAC
RUNX2-enh3-R	human	CCAAAACCCTCTCCCAGAAC

Supplementary Table 3

SOX10 shRNA constructs							
name	species	sequence	strand	order number	target region	type	vector
non-targeting (NT)	human	CCGGCAACAAGATGAAGAGCACCAACTCGAGTTGGTGCTCTTCATCTTGTTGTTTTT	sense	SHC002 from Sigma	non-targeting	constitutive	pLKO.1
Gilbert et al., Cell 2014; 159:647–661	human	AATTAAAAACAACAAGATGAAGAGCACCAACTCGAGTTGGTGCTCTTCATCTTGTTG	antisense				
human shSOX10-1	human	CCGGCCTCATTCTTTGTCTGAGAAACTCGAGTTTCTCAGACAAAGAATGAGGTTTTTG	sense	TRCN0000018984	3' UTR	constitutive	pLKO.1
	human	AATTCAAAAACCTCATTCTTTGTCTGAGAAACTCGAGTTTCTCAGACAAAGAATGAGG	antisense				
human shSOX10-2	human	CCGGGCTGCTGAACGAAAGTGACAACTCGAGTTGTCACTTTCGTTCAGCAGCTTTTTG	sense	TRCN0000018988	CDS	constitutive	pLKO.1
	human	AATTCAAAAAGCTGCTGAACGAAAGTGACAACTCGAGTTGTCACTTTCGTTCAGCAGC	antisense				
human shSOX10-3	human	CCGGGCAGCCAGTATATACGACACTCTCGAGAGTGTCGTATATACTGGCTGCTTTTTG	sense	TRCN0000018987	CDS	constitutive	pLKO.1
	human	AATTCAAAAAGCAGCCAGTATATACGACACTCTCGAGAGTGTCGTATATACTGGCTGC	antisense				
pLKO-Tet-On-nontarget shRNA	human	CCGGCAACAAGATGAAGAGCACCAACTCGAGTTGGTGCTCTTCATCTTGTTGTTTTT	sense	SHC002 from Sigma	non-targeting	inducible	pLKO-Tet-On
	human	AATTAAAAACAACAAGATGAAGAGCACCAACTCGAGTTGGTGCTCTTCATCTTGTTG	antisense				
human pLKO-Tet-On-shSOX10-2	human	CCGGCCGGGCTGCTGAACGAAAGTGACAACTCGAGTTGTCACTTTCGTTCAGCAGCCCGGTTTTT	sense	TRCN0000018988	CDS	inducible	pLKO-Tet-On
	human	AATTAAAAACCGGGCTGCTGAACGAAAGTGACAACTCGAGTTGTCACTTTCGTTCAGCAGCCCGG	antisense				
mouse shSox10	mouse	CCGGTTGCTCCAGCGATACCTTAATCTCGAGATTAAGGTATCGCTGGAGCAATTTTTG	sense	TRCN0000244290	3' UTR	constitutive	pLKO.1
	mouse	AATTCAAAAATTGCTCCAGCGATACCTTAATCTCGAGATTAAGGTATCGCTGGAGCAA	antisense				
CRISPR-Cas9 SOX10 kd constructs	species	sequence	strand		target region	type	vector
NT_1 (non-targeting-1)	human	TTGGCGCCAAACGTGCCCTGACGGGTTTAAGAGC	sense		non-targeting	constitutive	pLKO.2
Gilbert et al., Cell 2014; 159:647–661	human	TTAGCTCTTAAACCCGTCAGGGCACGTTTGGCGCCAACAAG	antisense				
NT_2 (non-targeting-2)	human	TTGGTGCGATGGGGGGGTGGGTAGCGTTTAAGAGC	sense		non-targeting	constitutive	pLKO.2
Gilbert et al., Cell 2014; 159:647–661	human	TTAGCTCTTAAACGCTACCCACCCCCCCATCGCACCAACAAG	antisense				
Sg1 (SOX10_Guide1)	human	TTGATTCAGGCTCCGTCCTAACGGTTTAAGAGC	sense		CDS	constitutive	pLKO.2
	human	TTAGCTCTTAAACCGTTAGGACGGAGCCTGAATCAACAAG	antisense				
Sg4 (SOX10_Guide4)	human	TTGCGAGCTGGACCGCACACCTTGTTTAAGAGC	sense		CDS	constitutive	pLKO.2
	human	TTAGCTCTTAAACAAGGTGTGCGGTCCAGCTCGCAACAAG	antisense				
Sg6 (SOX10_Guide6)	human	TTGAGTCTCGGGCTGTCCGGCCAGTTTAAGAGC	sense		5' UTR	constitutive	pLKO.2
	human	TTAGCTCTTAAACTGGCCGGACAGCCCGAGACTCAACAAG	antisense				

Supplementary Methods

General settings for sample-wise methylation features: methylation was represented as vectors of beta values, in which the order of data points corresponds to chromosomal CpG positions. Methylation features (partially methylated domains (PMDs), lowly methylated regions (LMRs), DNA methylation valleys (DMVs)) are defined based on the comparison of methylation measurements between genome segments. Each segment should be homogeneous in its methylation, relative to outside regions. We used the Bayesian framework and dynamic programming approach of fastseg (1.20.0) [1] to identify change points in the genome corresponding to segment borders.

As fastseg does not take consideration the distance between CpG sites, each chromosome was split into blocks if the gap between two adjacent CpG sites is larger than 100kb. Blocks containing less than 50 CpG sites were removed.

While fastseg can predict segment borders, each segment must still be classified based on its methylation. To do so, we examined the distribution of the mean and standard deviation of methylation in segments. The following plots visualize the distribution of segments' methylation means and standard deviations for all tumour samples and all normal samples, separated by subtype:

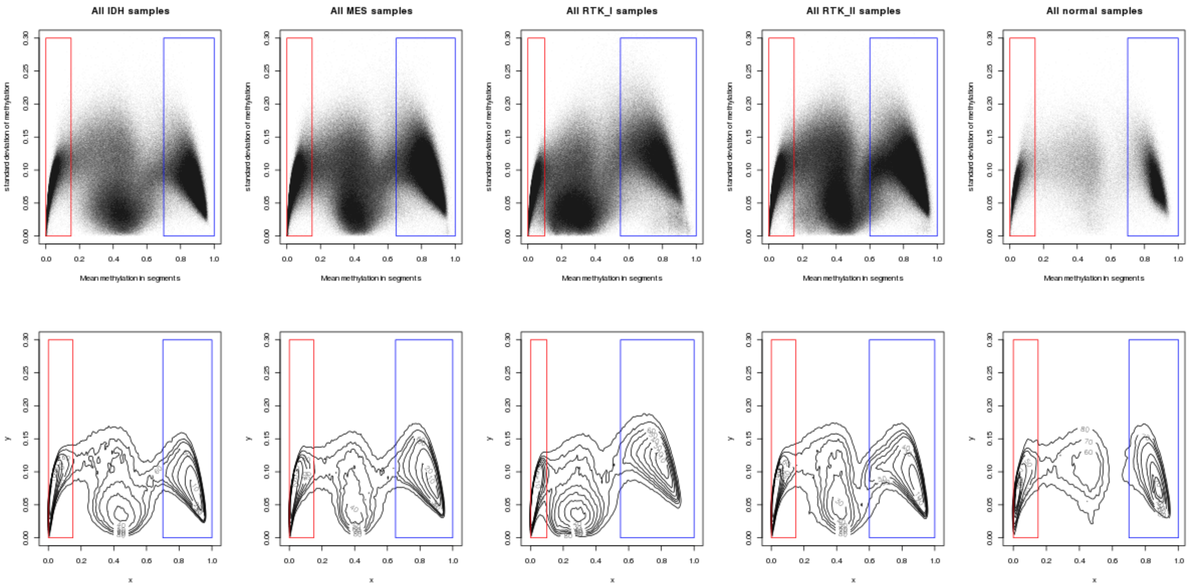

Supplementary Fig. 8. Methylation segment classification. Mean methylation (beta values; X-axis) are plotted against the standard deviation of methylation (Y-axis). Red and blue boxes indicate the low and high methylation segments for each glioblastoma subtype and normal brain samples. IDH, *n* = 12; MES, *n* = 19; RTK I, *n* = 12; RTK II, *n* = 17 samples.

It is clear that there are at least two major clusters of data points: one corresponding to low methylation and the other corresponding to high methylation. The remaining points form a sparse and heterogeneous intermediate group. According to the distribution of the points, we categorized segments into three groups using the following cutoffs:

- Group 1 segments, low methylation: IDH: mean < 0.15; MES: mean < 0.15; RTK I: mean < 0.10; RTK II: mean < 0.15. (marked by red rectangles)
- Group 3 segments, high methylation: IDH: mean > 0.7; MES: mean > 0.65; RTK I: mean > 0.55; RTK II: mean > 0.60. (marked by blue rectangles)
- Group 2 segments, intermediate methylation: all remaining segments.

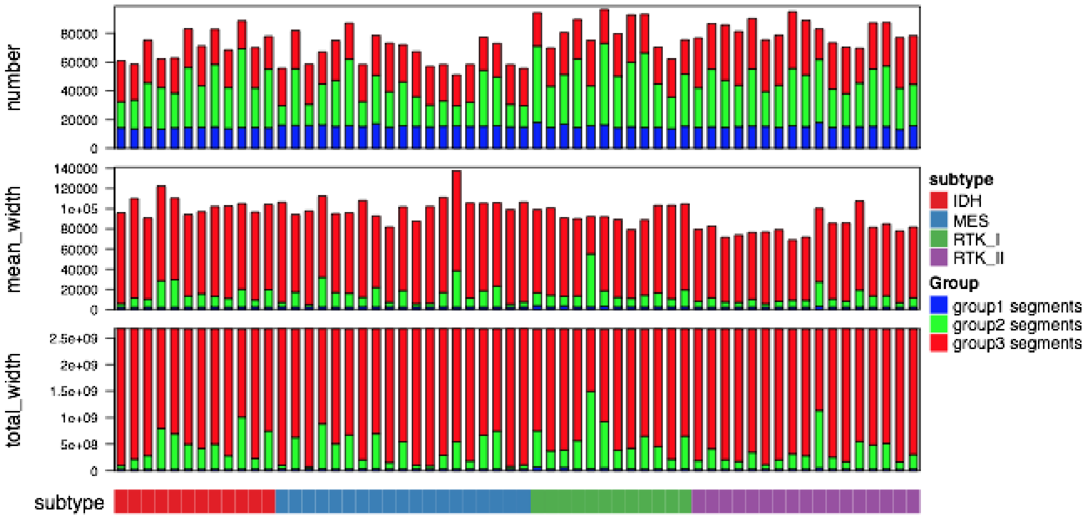

Supplementary Fig. 9. Methylation segment features. Comparison of the three methylation segment types based on their number and length in all subtypes. IDH, *n* = 12; MES, *n* = 19; RTK I, *n* = 12; RTK II, *n* = 17 samples.

The above summary plot shows the number of segments, mean segment width and the total number of base pairs of segments in all tumour samples. We observe overall that there are more group 2 segments by absolute number, but group 3 segments are much longer on average and represent the majority of the genome. For some samples which show global hypomethylation (RTK I subgroup), the group 2 segments are longer and represent most of the genome. Group 1 segments are short and occupy much smaller proportions of the genome. Next we define different methylation features based on the three sets of segments.

DNA methylation valleys. DNA methylation valleys (DMVs) are regions showing very low methylation. One major feature identifying DMVs is that they are highly hypomethylated compared to the flanking regions. As such, we looked for DMVs in group 1 segments. For each segment, we denote the mean methylation as m, we calculated the absolute methylation difference to its two neighbouring segments:

$$m_{diff} = \min(|m - m_{left}|, |m - m_{right}|)$$

and to meet the assumption that methylation drops within DMVs, we defined an additional criterion:

$$m < m_{left} \text{ and } m < m_{right}$$

To determine the cutoff of m_{diff}, we plotted m_{diff} vs segment width for all tumour samples and all normal samples. The distributions in the following plots are quite homogeneous. For DMVs, we simply set $m_{diff} > 0.5$ for IDH/MES/RTK II/normal while $m_{diff} > 0.3$ for RTK I (vertical dashed lines).

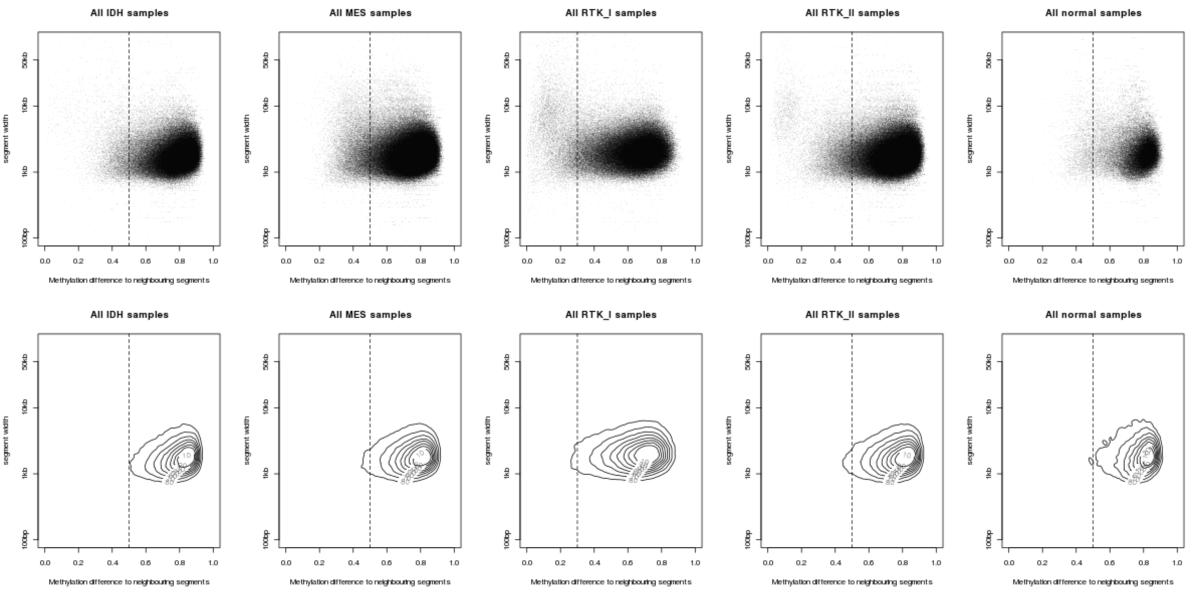

Supplementary Fig. 10. Identification of DNA methylation valleys. Methylation difference to neighbouring segments (beta values; X-axis) are plotted against the segment widths (Y-axis). The five plots in the first row were made as scatter plots and the plots in the second row were made as contour plots for visualizing the 2D density distributions of data points. Vertical dash lines corresponded to the cutoffs for selecting DMVs. IDH, *n* = 12; MES, *n* = 19; RTK I, *n* = 12; RTK II, *n* = 17 samples.

Lowly methylated regions: Similar to DMVs, lowly methylated regions (LMRs) also show lower methylation compared to neighbouring segments; however, the absolute methylation level is not as low as in DMVs. We searched for LMRs in group 2 segments. As with DMVs, we plot the absolute methylation difference versus the width of group 2 segments. In contrast to the same plots for DMVs, we observed two major clusters in these plots. One cluster corresponds to relatively higher methylation with shorter widths, and the other to relatively lower methylation with longer widths. As LMRs are normally short regions, we defined segments as LMRs if the methylation difference is larger than 0.26 and the width is less than 5kb (green rectangles on plot).

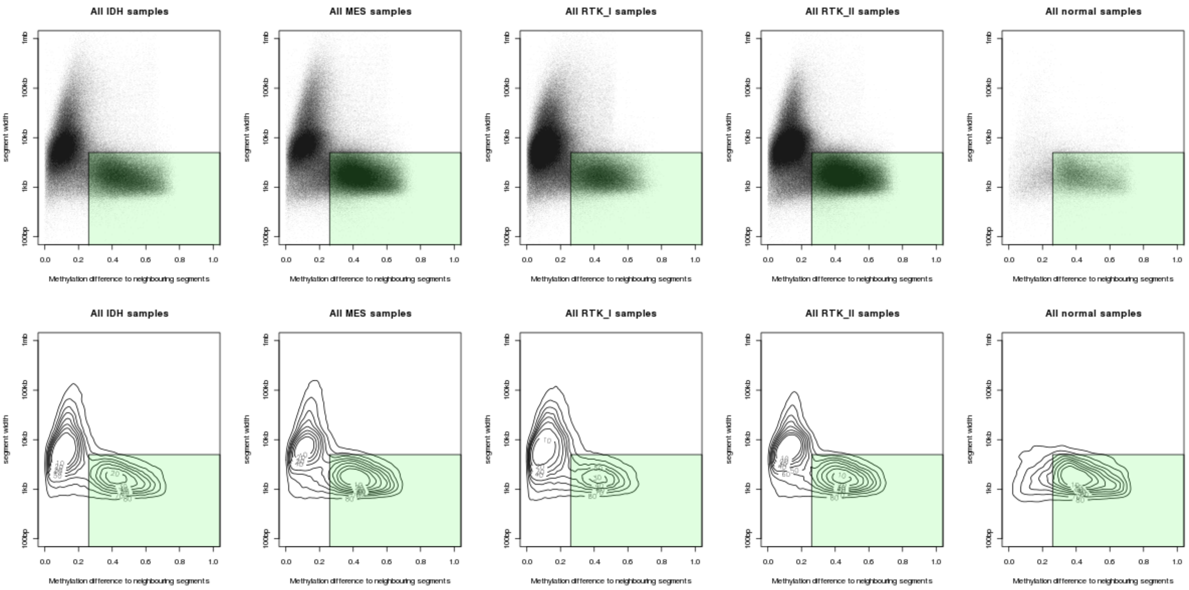

Supplementary Fig. 11. Identification of lowly methylated region. Methylation difference to neighbouring segments (beta values; X-axis) are plotted against the segment widths (Y-axis). The five plots in the first row were made as scatter plots and the plots in the second row were made as contour plots for visualizing the 2D density distributions of data points. Green rectangles covered the segments that were selected as LMRs. IDH, *n* = 12; MES, *n* = 19; RTK I, *n* = 12; RTK II, *n* = 17 samples.

Partially methylated domains. MethylSeekR (1.14.0) [2] was used to detect PMDs. The PMD segments are filtered by width > 10kb.

Consensus regions: As DMV/LMR/PMD are called for each sample separately, we defined the subtype-wide consensus feature set as follows. For k sets of e.g. LMRs, segments with a cross-sample coverage larger than or equal to 4 are kept as subtype-level consensus LMRs. Finally neighbouring LMRs are merged if the distance between them is less than 1kb, to minimise the number of small segments.

Enrichment of genomic features: we adopted the following method to calculate the enrichment of a set of methylation features (*e.g.* LMRs) in another set of genomic features (*e.g.* genes or certain chromatin states). We calculated the overlap of the two sets of regions of interest using the Jaccard coefficient, defined as the total number of basepairs in the intersection of the two sets divided by the total number of basepairs in the two sets' union. The significance of this overlap is calculated by comparison to a background set of genomic regions with a comparable CpG content. Background regions were selected as follows steps: 1) Select a proper window for the methylation feature sets. Regions in methylation feature sets are split into windows with size w, defined as the 25th quantile of all widths in that set, rounded to the thousandth digit. If w is smaller than 1kb or larger than 10kb, it is set to that value. Small windows with width less than $w/4$ are filtered out. 2) Calculate the CpG content for those windows. For this filtered window list, the number of CpG sites per 1kb is calculated and denoted as a vector $\boldsymbol{p}$. 3) Select genomic background with similar CpG content. The number of CpG sites per 1kb is also calculated for all 1kb windows in the genome, denoted as $\boldsymbol{p}_g$, and appropriate CpG-matched background genomic regions are selected by picking windows with p_g between the 5th and 95th percentiles of $\boldsymbol{p}$ from the methylation feature window list. The background distribution of overlaps is calculated by randomly permuting the input methylation regions with these CpG-matched background regions 1000 times using bedtools (v2.27.1) and calculating the Jaccard coefficient. Finally, a *z*-score of enrichment is calculated, $(s - \mu)/\sigma$, where s is the Jaccard coefficient for the two sets of regions and μ and σ are the mean and standard deviation of the Jaccard coefficient in the random permutation.

Supplementary References

1. Klambauer, G. *et al.* cn.MOPS: mixture of Poissons for discovering copy number variations in next-generation sequencing data with a low false discovery rate. *Nucleic Acids Res.* **40,** e69 (2012).
2. Burger, L., Gaidatzis, D., Schübeler, D. & Stadler, M. B. Identification of active regulatory regions from DNA methylation data. *Nucleic Acids Res.* **41,** e155 (2013).
