# Supplementary material for: Glioblastoma epigenome profiling identifies SOX10 as a master regulator of molecular tumour subtype
Source: Nat Commun. 2020 Dec 18;11:6434. doi: 10.1038/s41467-020-20225-w (PMC7749178; doi:10.1038/s41467-020-20225-w)
Supplement: Supplementary file 2 — Description of Additional Supplementary Files [file 41467_2020_20225_MOESM2_ESM.pdf]

Description of Additional Supplementary Files

File Name: Supplementary Data 1
Description: Patient Data

File Name: Supplementary Data 2
Description: MGMT methylation status

File Name: Supplementary Data 3
Description: WGBS QC

File Name: Supplementary Data 4
Description: RNA-seq QC

File Name: Supplementary Data 5
Description: ChIP-seq QC

File Name: Supplementary Data 6
Description: Methylation classifier probe IDs

File Name: Supplementary Data 7
Description: Master regulator lists

File Name: Supplementary Data 8
Description: RTN cohort A samplesheet

File Name: Supplementary Data 9
Description: RTN cohort B samplesheet

File Name: Supplementary Data 10
Description: Limma subtype signatures

File Name: Supplementary Data 11
Description: RTN results

File Name: Supplementary Data 12
Description: GSEA signatures

File Name: Supplementary Data 13
Description: ZH487 ATAC-seq DiffBind

File Name: Supplementary Data 14
Description: LN229 ATAC-seq DiffBind
