# Supplementary material for: Glioblastoma epigenome profiling identifies SOX10 as a master regulator of molecular tumour subtype
Source: Nat Commun. 2020 Dec 18;11:6434. doi: 10.1038/s41467-020-20225-w (PMC7749178; doi:10.1038/s41467-020-20225-w)
Supplement: Supplementary file 17 — Reporting Summary [file 41467_2020_20225_MOESM17_ESM.pdf]

nature research

Corresponding author(s): Bernhard Radlwimmer, Carl Herrman

Reporting Summary

Nature Research wishes to improve the reproducibility of the work that we publish. This form provides structure for consistency and transparency in reporting. For further information on Nature Research policies, see Authors & Referees and the Editorial Policy Checklist.

Statistical parameters

When statistical analyses are reported, confirm that the following items are present in the relevant location (e.g. figure legend, table legend, main text, or Methods section).

n/a	Confirmed	
☐	☒	The exact sample size (n) for each experimental group/condition, given as a discrete number and unit of measurement
☐	☒	An indication of whether measurements were taken from distinct samples or whether the same sample was measured repeatedly
☐	☒	The statistical test(s) used AND whether they are one- or two-sided<br>*Only common tests should be described solely by name; describe more complex techniques in the Methods section.*
☒	☐	A description of all covariates tested
☐	☒	A description of any assumptions or corrections, such as tests of normality and adjustment for multiple comparisons
☐	☒	A full description of the statistics including central tendency (e.g. means) or other basic estimates (e.g. regression coefficient) AND variation (e.g. standard deviation) or associated estimates of uncertainty (e.g. confidence intervals)
☐	☒	For null hypothesis testing, the test statistic (e.g. F, t, r) with confidence intervals, effect sizes, degrees of freedom and P value noted<br>*Give P values as exact values whenever suitable.*
☐	☒	For Bayesian analysis, information on the choice of priors and Markov chain Monte Carlo settings
☒	☐	For hierarchical and complex designs, identification of the appropriate level for tests and full reporting of outcomes
☐	☒	Estimates of effect sizes (e.g. Cohen's d, Pearson's r), indicating how they were calculated
☐	☒	Clearly defined error bars<br>*State explicitly what error bars represent (e.g. SD, SE, CI)*

Our web collection on statistics for biologists may be useful.

Software and code

Policy information about availability of computer code

Data collection	No software was used for data collection.
Data analysis	The following software were used:<br>version STAR 2.3.0e<br>htseq-count 0.6.0<br>bwa-mem 0.7.8<br>BEDTools 2.27.1<br>MethylSeekR 1.14.0<br>ROSE2 https://github.com/BradnerLab/pipeline/;<br>bigWigAverageOverBed v2<br>GSEA v3.0;<br>OMER 4.9.1<br>featureCounts in Subread 1.5.3<br>methylCtools v. 1.0.0<br>Snakemake v. 3.13.3<br>TrimGalore v. 0.4.2 (https://github.com/FelixKrueger/TrimGalore)<br>deepTools2 suite v. 2.4.1 (with subsequent tools SES, bamCompare,..)

MACS2 v. 2.1.1.20160309 (https://github.com/taoliu/MACS)
BowTie2 v. 2.3.4.3
samtools v. 1.3.1
SICER v. 1.1 (Ref. 22)
ChromHMM v. 1.19
CRCmapper commit b23ad55 on https://bitbucket.org/young_computation/crcmapper/ (Ref. 13)

R analyses were performed using v3.4.3 or 3.5.1, with the following packages: DESeq2 1.14.1, edgeR 3.20.1, limma 3.34.4, GSVA 1.26.0, ESTIMATE 1.0.13, clusterProfiler 3.6.0, bsseq 1.10.0, MethylSeekR 1.14.0, cola v1.0.0,, gcrma 2.50.0, sva 3.26.0, RTN 2.3.4, ggplot2 2.2.1, circlize 0.4.6, ComplexHeatmap 1.19.1, EnrichedHeatmap 1.9.2, Gviz 1.22.3, epik version hash: d3898bd, minfi 1.24.0, conumee 1.3.0; monocle 2.10.1; viper 1.14.0; Rtsne 0.13; ChIPpeakAnno 3.12.4; TxDb.Hsapiens.UCSC.hg19.knownGene 3.2.2; DiffBind 2.6.6

Extensive customised analysis scripts were used in this project. These will be deposited online at the project repository: https://github.com/dkfz-b060

For manuscripts utilizing custom algorithms or software that are central to the research but not yet described in published literature, software must be made available to editors/reviewers upon request. We strongly encourage code deposition in a community repository (e.g. GitHub). See the Nature Research guidelines for submitting code & software for further information.

Data

Policy information about availability of data

All manuscripts must include a data availability statement. This statement should provide the following information, where applicable:

- Accession codes, unique identifiers, or web links for publicly available datasets
- A list of figures that have associated raw data
- A description of any restrictions on data availability

The processed data analysed in this study is available at the GEO (SuperSeries GSE121723 [https://www.ncbi.nlm.nih.gov/geo/query/acc.cgi?acc=GSE121723]). Raw sequencing data is protected and controlled access available via the EGA (accession EGAS00001003230 and EGAS00001003953). The gene expression datasets used for the RTN analysis were obtained from E-MTAB-3073 (https://www.ebi.ac.uk/arrayexpress/experiments/E-MTAB-3073/), and GSE4290, GSE7696, GSE16011 and GSE43378 (https://www.ncbi.nlm.nih.gov/geo/query). The remaining data are available within the Article, Supplementary Information or available from the authors upon request.

Field-specific reporting

Please select the best fit for your research. If you are not sure, read the appropriate sections before making your selection.

[x] Life sciences [ ] Behavioural & social sciences [ ] Ecological, evolutionary & environmental sciences

For a reference copy of the document with all sections, see nature.com/authors/policies/ReportingSummary-flat.pdf

Life sciences study design

All studies must disclose on these points even when the disclosure is negative.

Sample size	No pre-calculation of sample size was performed before the study. As the aim of this project was to comprehensively analyse the 4 subtypes of GB, a balanced design with equal representation from each tumour subtype was chosen, subject to sample availability.
Data exclusions	No data were excluded. All figures and tables were created by the authors YW, MF, TG, CH and BR.
Replication	All computer code was written to allow reproduction of the analysis and this was performed several times to ensure that the results remained the same. Sequencing and microarray analyses of primary tumour samples were performed once per sample. Microarray analysis for GSEA were performed 2-6 times per sample, Co-IP of BCAT1 and BRD4 was done twice, qPCR and IHC of mouse tumor samples 3-5 times, and ChIP-PCR of cell lines once. ATAC-Seq analyses were done in duplicate. The group sizes of the mouse syngraft experiments were 9 and 10 animals, and the migration/invasion data derive from analyzing 10-21 wells or cells from one biological replicate.
Randomization	No randomization was performed in this study.
Blinding	Blinding for the analysis of patient samples was not relevant to our study because the data analysis was focused on identifying differences between the subtypes, and hence class labels were required.

Reporting for specific materials, systems and methods

Materials & experimental systems

n/a	Involved in the study
☒	☐ Unique biological materials
☐	☒ Antibodies
☐	☒ Eukaryotic cell lines
☒	☐ Palaeontology
☐	☒ Animals and other organisms
☐	☒ Human research participants

Methods

n/a	Involved in the study
☐	☒ ChIP-seq
☒	☐ Flow cytometry
☐	☒ MRI-based neuroimaging

Antibodies

Antibodies used

Goat anti-human SOX10 (Western blot) Santa Cruz Cat#sc-17342
Rabbit anti-human SOX10 (ChIP-seq, Co-IP) Abcam Cat#ab155279, lot GR113617-51
Rabbit polyclonal BRD4 Ab (ChIP-seq, WB and ChIP-qPCR) Bethyl Lab. Cat#A301-985A100
H3K27Ac (GB patient ChIP-seq) Active Motif Cat#AM#39133, lot 5
H3K4me1(GB patient ChIP-seq) Active Motif Cat#AM#38297, lot 1
H3K4me3 (GB patient ChIP-seq) Active Motif Cat#AM#39159, lot 2
H3K9me3 (GB patient ChIP-seq) Active Motif Cat#AM#39161, lot 3
H3K27me3 (GB patient ChIP-seq) Millipore Cat##07-449, lot 2382150
H3K36me3 (GB patient ChIP-seq) Active Motif Cat#AM#61101, lot 7
Rabbit polyclonal to Histone H3 (cell line ChIP-seq) Abcam Cat#ab1791
Rabbit polyclonal to Histone H3K4me1 (cell line ChIP-seq) Abcam Cat#ab8895
Rabbit polyclonal to Histone H3K4me3 (cell line ChIP-seq) Abcam Cat#ab8580
Rabbit polyclonal to Histone H3K9me3 (cell line ChIP-seq) Abcam Cat#ab8898
Rabbit polyclonal to Histone H3K27ac (cell line ChIP-seq) Abcam Cat#ab4729
Rabbit polyclonal to Histone H3K36me3 (cell line ChIP-seq) Abcam Cat#ab9050
Rabbit polyclonal to Histone H3K27me3 (cell line ChIP-seq) Abcam Cat#ab6002
Rabbit anti-Iba1 antibody (IHC), Wako, Cat#019-19741
alpha-Tubulin Sigma Cat#T-9026

Validation

Goat anti-human SOX10 (Western blot) Santa Cruz Cat#sc-17342
(Santa Cruz Biotechnology Cat# sc-17342, RRID:AB_2195374), Lot#?
https://antibodyregistry.org/search.php?q=AB_2195374

Rabbit anti-human SOX10 (ChIP-seq, Co-IP) Abcam Cat#ab155279, lot GR113617-51
(Abcam Cat# ab155279, RRID:AB_2650603), Lot#?
https://antibodyregistry.org/search.php?q=AB_2650603

Rabbit polyclonal BRD4 Ab (ChIP-seq, WB and ChIP-qPCR) Bethyl Lab. Cat#A301-985A100
(Thermo Fisher Scientific Cat# A301-985A100, RRID:AB_2620184), Lot#?
https://antibodyregistry.org/search.php?q=AB_2620184%3c/result%3e

H3K27Ac (GB patient ChIP-seq) Active Motif Cat#AM#39133, lot 5
(Active Motif Cat# 39133, RRID:AB_2561016), Lot#?
https://antibodyregistry.org/search.php?q=AB_2561016

H3K4me1(GB patient ChIP-seq) Active Motif Cat#AM#39297, lot 1
(Active Motif Cat# 39297, RRID:AB_2615075), Lot#?
https://antibodyregistry.org/search.php?q=AB_2615075

H3K4me3 (GB patient ChIP-seq) Active Motif Cat#AM#39159, lot 2
(Active Motif Cat# 39159, RRID:AB_2615077), Lot#?
https://antibodyregistry.org/search.php?q=AB_2615077

H3K9me3 (GB patient ChIP-seq) Active Motif Cat#AM#39161, lot 3
(Active Motif Cat# 39161, RRID:AB_2532132), Lot#?
https://antibodyregistry.org/search.php?q=AB_2532132

H3K27me3 (GB patient ChIP-seq) Millipore Cat##07-449, lot 2382150
(Millipore Cat# 07-449, RRID:AB_310624), Lot#?
https://antibodyregistry.org/search.php?q=AB_310624

H3K36me3 (GB patient ChIP-seq) Active Motif Cat#AM#61101, lot 7
(Active Motif Cat# 61101, RRID:AB_2615073), Lot#?
https://antibodyregistry.org/search.php?q=AB_2615073

Rabbit polyclonal to Histone H3 (cell line ChIP-seq) Abcam Cat#ab1791
(Abcam Cat# ab1791, RRID:AB_302613), Lot#?
https://antibodyregistry.org/search.php?q=AB_302613

Rabbit polyclonal to Histone H3K4me1 (cell line ChIP-seq) Abcam Cat#ab8895
(Abcam Cat# ab8895, RRID:AB_306847), Lot#?
https://antibodyregistry.org/search.php?q=AB_306847

Rabbit polyclonal to Histone H3K4me3 (cell line ChIP-seq) Abcam Cat#ab8580
(Abcam Cat# ab8580, RRID:AB_306649), Lot#?
https://antibodyregistry.org/search.php?q=AB_306649

Rabbit polyclonal to Histone H3K9me3 (cell line ChIP-seq) Abcam Cat#ab8898
(Abcam Cat# ab8898, RRID:AB_306848), Lot#?
https://antibodyregistry.org/search.php?q=AB_306848

Rabbit polyclonal to Histone H3K27ac (cell line ChIP-seq) Abcam Cat#ab4729
(Abcam Cat# ab4729, RRID:AB_2118291), Lot#?
https://antibodyregistry.org/search.php?q=AB_2118291

Rabbit polyclonal to Histone H3K36me3 (cell line ChIP-seq) Abcam Cat#ab9050
(Abcam Cat# ab9050, RRID:AB_306966), Lot#?
https://antibodyregistry.org/search.php?q=AB_306966

Rabbit polyclonal to Histone H3K27me3 (cell line ChIP-seq) Abcam Cat#ab6002
(Abcam Cat# ab6002, RRID:AB_305237), Lot#?
https://antibodyregistry.org/search.php?q=AB_305237

Rabbit anti-Iba1 antibody (IHC), Wako, Cat#019-19741
(Wako Cat# 019-19741, RRID:AB_839504), Lot#?
https://antibodyregistry.org/search.php?q=AB_839504

alpha-Tubulin Sigma Cat#T-9026
(Sigma-Aldrich Cat# T9026, RRID:AB_477593), Lot#?
https://antibodyregistry.org/search.php?q=AB_477593%3c/result%3e

Anti-mouse IgG, HRP-linked Antibody, Cell Signaling Technology #7076 , 1: 5000
(Cell Signaling Technology Cat# 7076, RRID:AB_330924), Lot#?
https://antibodyregistry.org/search.php?q=AB_330924

Anti-rabbit IgG, HRP-linked Antibody, Cell Signaling Technology #7074, 1:5000
(Cell Signaling Technology Cat# 7074, RRID:AB_2099233), Lot#?
https://antibodyregistry.org/search.php?q=AB_2099233

mouse anti-goat IgG-HRP, Santa Cruz sc-2354, 1:5000
(Santa Cruz Biotechnology Cat# sc-2354, RRID:AB_628490), Lot#?
https://antibodyregistry.org/search.php?q=AB_628490

Goat anti-Chicken IgY (H+L) Secondary Antibody, Alexa Fluor 488, Invitrogen A-11039, 1:1000
(Molecular Probes Cat# A-11039, RRID:AB_142924), Lot#?
https://antibodyregistry.org/search.php?q=AB_142924

Eukaryotic cell lines

Policy information about cell lines

Cell line source(s)

LN229, ATCC, Cat# CRL-2611
ZH487, GB patient-derived, Zurich University
HEK-293T, ATCC, Cat#CRL-3216

	mGB1, DKFZ, Peter Angel's lab
Authentication	Cell line identity of LN229 was confirmed by STR profiling (cell line authentication service by Multiplexion GmbH, Germany). The cell lines ZH487, HEK293T and mGB1 were not authenticated.
Mycoplasma contamination	The cell lines LN229 and ZH487, HEK293T and mGB1 were confirmed to be mycoplasm free (test performed by Multiplexion GmbH, Germany).
Commonly misidentified lines (See ICLAC register)	No commonly misidentified cell line was used in this study

Animals and other organisms

Policy information about studies involving animals; ARRIVE guidelines recommended for reporting animal research

Laboratory animals	Wild type female C57-BL6 mice were obtained from the Jackson laboratory and housed at an ambient temperature of 22.0 ± 2.0° C and 55.0 ± 10.0% humidity with a lights-on and lights-off times of 12 hours starting at 7 am and 7 pm, respectively. Tumor cells were injected at an age of 6 weeks (NT: n=10 and shSox10 n=9). MRI based neuroimaging was performed in the DKFZ MRI core facility on 57 days post-injection.
Wild animals	No wild animals were used in the study.
Field-collected samples	No field-collected samples were used in the study.

Human research participants

Policy information about studies involving human research participants

Population characteristics	The patient cohort consisted of 32 males and 28 females with an average age of 52.5 ± 11 years (mean and standard deviation). IDH1 mutations were present in 12 samples. All patients received standard therapy consisting of radiotherapy and Temozolamide treatment.
Recruitment	Retrospective study. No recruitment.

ChIP-seq

Data deposition

- [x] Confirm that both raw and final processed data have been deposited in a public database such as GEO.
- [x] Confirm that you have deposited or provided access to graph files (e.g. BED files) for the called peaks.

Data access links<br>*May remain private before publication.*	GEO (processed data): see SuperSeries GSE121723, https://www.ncbi.nlm.nih.gov/geo/query/acc.cgi?acc=GSE121723<br><br>EGA: under study accession number EGAS00001003953
Files in database submission	AK066_H3K27ac_coverage_SES_subtract.bigWig<br>AK066_H3K27ac_peaks.narrowPeak.bed<br>AK066_H3K27ac_W200-G600-FDR0.01-island.bed<br>AK066_H3K27me3_coverage_SES_subtract.bigWig<br>AK066_H3K27me3_peaks.narrowPeak.bed<br>AK066_H3K27me3_W200-G600-FDR0.01-island.bed<br>AK066_H3K4me1_coverage_SES_subtract.bigWig<br>AK066_H3K4me1_peaks.narrowPeak.bed<br>AK066_H3K4me1_W200-G600-FDR0.01-island.bed<br>AK066_H3K4me3_coverage_SES_subtract.bigWig<br>AK066_H3K4me3_peaks.narrowPeak.bed<br>AK066_H3K4me3_W200-G600-FDR0.01-island.bed<br>AK066_H3K9me3_coverage_SES_subtract.bigWig<br>AK066_H3K9me3_peaks.narrowPeak.bed<br>AK066_H3K9me3_W200-G600-FDR0.01-island.bed<br>AK071_ChromHMM_18states.bed<br>AK071_ChromHMM_simplified_7states.bed<br>AK071_H3K27ac_coverage_SES_subtract.bigWig<br>AK071_H3K27ac_peaks.narrowPeak.bed<br>AK071_H3K27ac_W200-G600-FDR0.01-island.bed<br>AK071_H3K27me3_coverage_SES_subtract.bigWig<br>AK071_H3K27me3_peaks.narrowPeak.bed<br>AK071_H3K27me3_W200-G600-FDR0.01-island.bed<br>AK071_H3K36me3_coverage_SES_subtract.bigWig

AK071_H3K36me3_peaks.narrowPeak.bed
AK071_H3K36me3_W200-G600-FDR0.01-island.bed
AK071_H3K4me1_coverage_SES_subtract.bigWig
AK071_H3K4me1_peaks.narrowPeak.bed
AK071_H3K4me1_W200-G600-FDR0.01-island.bed
AK071_H3K4me3_coverage_SES_subtract.bigWig
AK071_H3K4me3_peaks.narrowPeak.bed
AK071_H3K4me3_W200-G600-FDR0.01-island.bed
AK071_H3K9me3_coverage_SES_subtract.bigWig
AK071_H3K9me3_peaks.narrowPeak.bed
AK071_H3K9me3_W200-G600-FDR0.01-island.bed
AK076_ChromHMM_18states.bed
AK076_ChromHMM_simplified_7states.bed
AK076_H3K27ac_coverage_SES_subtract.bigWig
AK076_H3K27ac_peaks.narrowPeak.bed
AK076_H3K27ac_W200-G600-FDR0.01-island.bed
AK076_H3K27me3_coverage_SES_subtract.bigWig
AK076_H3K27me3_peaks.narrowPeak.bed
AK076_H3K27me3_W200-G600-FDR0.01-island.bed
AK076_H3K36me3_coverage_SES_subtract.bigWig
AK076_H3K36me3_peaks.narrowPeak.bed
AK076_H3K36me3_W200-G600-FDR0.01-island.bed
AK076_H3K4me1_coverage_SES_subtract.bigWig
AK076_H3K4me1_peaks.narrowPeak.bed
AK076_H3K4me1_W200-G600-FDR0.01-island.bed
AK076_H3K4me3_coverage_SES_subtract.bigWig
AK076_H3K4me3_peaks.narrowPeak.bed
AK076_H3K4me3_W200-G600-FDR0.01-island.bed
AK076_H3K9me3_coverage_SES_subtract.bigWig
AK076_H3K9me3_peaks.narrowPeak.bed
AK076_H3K9me3_W200-G600-FDR0.01-island.bed
AK089_ChromHMM_18states.bed
AK089_ChromHMM_simplified_7states.bed
AK089_H3K27ac_coverage_SES_subtract.bigWig
AK089_H3K27ac_peaks.narrowPeak.bed
AK089_H3K27ac_W200-G600-FDR0.01-island.bed
AK089_H3K27me3_coverage_SES_subtract.bigWig
AK089_H3K27me3_peaks.narrowPeak.bed
AK089_H3K27me3_W200-G600-FDR0.01-island.bed
AK089_H3K36me3_coverage_SES_subtract.bigWig
AK089_H3K36me3_peaks.narrowPeak.bed
AK089_H3K36me3_W200-G600-FDR0.01-island.bed
AK089_H3K4me1_coverage_SES_subtract.bigWig
AK089_H3K4me1_peaks.narrowPeak.bed
AK089_H3K4me1_W200-G600-FDR0.01-island.bed
AK089_H3K4me3_coverage_SES_subtract.bigWig
AK089_H3K4me3_peaks.narrowPeak.bed
AK089_H3K4me3_W200-G600-FDR0.01-island.bed
AK089_H3K9me3_coverage_SES_subtract.bigWig
AK089_H3K9me3_peaks.narrowPeak.bed
AK089_H3K9me3_W200-G600-FDR0.01-island.bed
AK091_ChromHMM_18states.bed
AK091_ChromHMM_simplified_7states.bed
AK091_H3K27ac_coverage_SES_subtract.bigWig
AK091_H3K27ac_peaks.narrowPeak.bed
AK091_H3K27ac_W200-G600-FDR0.01-island.bed
AK091_H3K27me3_coverage_SES_subtract.bigWig
AK091_H3K27me3_peaks.narrowPeak.bed
AK091_H3K27me3_W200-G600-FDR0.01-island.bed
AK091_H3K36me3_coverage_SES_subtract.bigWig
AK091_H3K36me3_peaks.narrowPeak.bed
AK091_H3K36me3_W200-G600-FDR0.01-island.bed
AK091_H3K4me1_coverage_SES_subtract.bigWig
AK091_H3K4me1_peaks.narrowPeak.bed
AK091_H3K4me1_W200-G600-FDR0.01-island.bed
AK091_H3K4me3_coverage_SES_subtract.bigWig
AK091_H3K4me3_peaks.narrowPeak.bed
AK091_H3K4me3_W200-G600-FDR0.01-island.bed

AK091_H3K9me3_coverage_SES_subtract.bigWig
AK091_H3K9me3_peaks.narrowPeak.bed
AK091_H3K9me3_W200-G600-FDR0.01-island.bed
AK100_ChromHMM_18states.bed
AK100_ChromHMM_simplified_7states.bed
AK100_H3K27ac_coverage_SES_subtract.bigWig
AK100_H3K27ac_peaks.narrowPeak.bed
AK100_H3K27ac_W200-G600-FDR0.01-island.bed
AK100_H3K27me3_coverage_SES_subtract.bigWig
AK100_H3K27me3_peaks.narrowPeak.bed
AK100_H3K27me3_W200-G600-FDR0.01-island.bed
AK100_H3K36me3_coverage_SES_subtract.bigWig
AK100_H3K36me3_peaks.narrowPeak.bed
AK100_H3K36me3_W200-G600-FDR0.01-island.bed
AK100_H3K4me1_coverage_SES_subtract.bigWig
AK100_H3K4me1_peaks.narrowPeak.bed
AK100_H3K4me1_W200-G600-FDR0.01-island.bed
AK100_H3K4me3_coverage_SES_subtract.bigWig
AK100_H3K4me3_peaks.narrowPeak.bed
AK100_H3K4me3_W200-G600-FDR0.01-island.bed
AK100_H3K9me3_coverage_SES_subtract.bigWig
AK100_H3K9me3_peaks.narrowPeak.bed
AK100_H3K9me3_W200-G600-FDR0.01-island.bed
AK124_ChromHMM_18states.bed
AK124_ChromHMM_simplified_7states.bed
AK124_H3K27ac_coverage_SES_subtract.bigWig
AK124_H3K27ac_peaks.narrowPeak.bed
AK124_H3K27ac_W200-G600-FDR0.01-island.bed
AK124_H3K27me3_coverage_SES_subtract.bigWig
AK124_H3K27me3_peaks.narrowPeak.bed
AK124_H3K27me3_W200-G600-FDR0.01-island.bed
AK124_H3K36me3_coverage_SES_subtract.bigWig
AK124_H3K36me3_peaks.narrowPeak.bed
AK124_H3K36me3_W200-G600-FDR0.01-island.bed
AK124_H3K4me1_coverage_SES_subtract.bigWig
AK124_H3K4me1_peaks.narrowPeak.bed
AK124_H3K4me1_W200-G600-FDR0.01-island.bed
AK124_H3K4me3_coverage_SES_subtract.bigWig
AK124_H3K4me3_peaks.narrowPeak.bed
AK124_H3K4me3_W200-G600-FDR0.01-island.bed
AK124_H3K9me3_coverage_SES_subtract.bigWig
AK124_H3K9me3_peaks.narrowPeak.bed
AK124_H3K9me3_W200-G600-FDR0.01-island.bed
AK139_ChromHMM_18states.bed
AK139_ChromHMM_simplified_7states.bed
AK139_H3K27ac_coverage_SES_subtract.bigWig
AK139_H3K27ac_peaks.narrowPeak.bed
AK139_H3K27ac_W200-G600-FDR0.01-island.bed
AK139_H3K27me3_coverage_SES_subtract.bigWig
AK139_H3K27me3_peaks.narrowPeak.bed
AK139_H3K27me3_W200-G600-FDR0.01-island.bed
AK139_H3K36me3_coverage_SES_subtract.bigWig
AK139_H3K36me3_peaks.narrowPeak.bed
AK139_H3K36me3_W200-G600-FDR0.01-island.bed
AK139_H3K4me1_coverage_SES_subtract.bigWig
AK139_H3K4me1_peaks.narrowPeak.bed
AK139_H3K4me1_W200-G600-FDR0.01-island.bed
AK139_H3K4me3_coverage_SES_subtract.bigWig
AK139_H3K4me3_peaks.narrowPeak.bed
AK139_H3K4me3_W200-G600-FDR0.01-island.bed
AK139_H3K9me3_coverage_SES_subtract.bigWig
AK139_H3K9me3_peaks.narrowPeak.bed
AK139_H3K9me3_W200-G600-FDR0.01-island.bed
AK142_ChromHMM_18states.bed
AK142_ChromHMM_simplified_7states.bed
AK142_H3K27ac_coverage_SES_subtract.bigWig
AK142_H3K27ac_peaks.narrowPeak.bed
AK142_H3K27ac_W200-G600-FDR0.01-island.bed

AK142_H3K27me3_coverage_SES_subtract.bigWig
AK142_H3K27me3_peaks.narrowPeak.bed
AK142_H3K27me3_W200-G600-FDR0.01-island.bed
AK142_H3K36me3_coverage_SES_subtract.bigWig
AK142_H3K36me3_peaks.narrowPeak.bed
AK142_H3K36me3_W200-G600-FDR0.01-island.bed
AK142_H3K4me1_coverage_SES_subtract.bigWig
AK142_H3K4me1_peaks.narrowPeak.bed
AK142_H3K4me1_W200-G600-FDR0.01-island.bed
AK142_H3K4me3_coverage_SES_subtract.bigWig
AK142_H3K4me3_peaks.narrowPeak.bed
AK142_H3K4me3_W200-G600-FDR0.01-island.bed
AK142_H3K9me3_coverage_SES_subtract.bigWig
AK142_H3K9me3_peaks.narrowPeak.bed
AK142_H3K9me3_W200-G600-FDR0.01-island.bed
AK149_ChromHMM_18states.bed
AK149_ChromHMM_simplified_7states.bed
AK149_H3K27ac_coverage_SES_subtract.bigWig
AK149_H3K27ac_peaks.narrowPeak.bed
AK149_H3K27ac_W200-G600-FDR0.01-island.bed
AK149_H3K27me3_coverage_SES_subtract.bigWig
AK149_H3K27me3_peaks.narrowPeak.bed
AK149_H3K27me3_W200-G600-FDR0.01-island.bed
AK149_H3K36me3_coverage_SES_subtract.bigWig
AK149_H3K36me3_peaks.narrowPeak.bed
AK149_H3K36me3_W200-G600-FDR0.01-island.bed
AK149_H3K4me1_coverage_SES_subtract.bigWig
AK149_H3K4me1_peaks.narrowPeak.bed
AK149_H3K4me1_W200-G600-FDR0.01-island.bed
AK149_H3K4me3_coverage_SES_subtract.bigWig
AK149_H3K4me3_peaks.narrowPeak.bed
AK149_H3K4me3_W200-G600-FDR0.01-island.bed
AK149_H3K9me3_coverage_SES_subtract.bigWig
AK149_H3K9me3_peaks.narrowPeak.bed
AK149_H3K9me3_W200-G600-FDR0.01-island.bed
AK153_ChromHMM_18states.bed
AK153_ChromHMM_simplified_7states.bed
AK153_H3K27ac_coverage_SES_subtract.bigWig
AK153_H3K27ac_peaks.narrowPeak.bed
AK153_H3K27ac_W200-G600-FDR0.01-island.bed
AK153_H3K27me3_coverage_SES_subtract.bigWig
AK153_H3K27me3_peaks.narrowPeak.bed
AK153_H3K27me3_W200-G600-FDR0.01-island.bed
AK153_H3K36me3_coverage_SES_subtract.bigWig
AK153_H3K36me3_peaks.narrowPeak.bed
AK153_H3K36me3_W200-G600-FDR0.01-island.bed
AK153_H3K4me1_coverage_SES_subtract.bigWig
AK153_H3K4me1_peaks.narrowPeak.bed
AK153_H3K4me1_W200-G600-FDR0.01-island.bed
AK153_H3K4me3_coverage_SES_subtract.bigWig
AK153_H3K4me3_peaks.narrowPeak.bed
AK153_H3K4me3_W200-G600-FDR0.01-island.bed
AK153_H3K9me3_coverage_SES_subtract.bigWig
AK153_H3K9me3_peaks.narrowPeak.bed
AK153_H3K9me3_W200-G600-FDR0.01-island.bed
AK156_ChromHMM_18states.bed
AK156_ChromHMM_simplified_7states.bed
AK156_H3K27ac_coverage_SES_subtract.bigWig
AK156_H3K27ac_peaks.narrowPeak.bed
AK156_H3K27ac_W200-G600-FDR0.01-island.bed
AK156_H3K27me3_coverage_SES_subtract.bigWig
AK156_H3K27me3_peaks.narrowPeak.bed
AK156_H3K27me3_W200-G600-FDR0.01-island.bed
AK156_H3K36me3_coverage_SES_subtract.bigWig
AK156_H3K36me3_peaks.narrowPeak.bed
AK156_H3K36me3_W200-G600-FDR0.01-island.bed
AK156_H3K4me1_coverage_SES_subtract.bigWig
AK156_H3K4me1_peaks.narrowPeak.bed

AK156_H3K4me1_W200-G600-FDR0.01-island.bed
AK156_H3K4me3_coverage_SES_subtract.bigWig
AK156_H3K4me3_peaks.narrowPeak.bed
AK156_H3K4me3_W200-G600-FDR0.01-island.bed
AK156_H3K9me3_coverage_SES_subtract.bigWig
AK156_H3K9me3_peaks.narrowPeak.bed
AK156_H3K9me3_W200-G600-FDR0.01-island.bed
AK158_ChromHMM_18states.bed
AK158_ChromHMM_simplified_7states.bed
AK158_H3K27ac_coverage_SES_subtract.bigWig
AK158_H3K27ac_peaks.narrowPeak.bed
AK158_H3K27ac_W200-G600-FDR0.01-island.bed
AK158_H3K27me3_coverage_SES_subtract.bigWig
AK158_H3K27me3_peaks.narrowPeak.bed
AK158_H3K27me3_W200-G600-FDR0.01-island.bed
AK158_H3K36me3_coverage_SES_subtract.bigWig
AK158_H3K36me3_peaks.narrowPeak.bed
AK158_H3K36me3_W200-G600-FDR0.01-island.bed
AK158_H3K4me1_coverage_SES_subtract.bigWig
AK158_H3K4me1_peaks.narrowPeak.bed
AK158_H3K4me1_W200-G600-FDR0.01-island.bed
AK158_H3K4me3_coverage_SES_subtract.bigWig
AK158_H3K4me3_peaks.narrowPeak.bed
AK158_H3K4me3_W200-G600-FDR0.01-island.bed
AK158_H3K9me3_coverage_SES_subtract.bigWig
AK158_H3K9me3_peaks.narrowPeak.bed
AK158_H3K9me3_W200-G600-FDR0.01-island.bed
AK173_ChromHMM_18states.bed
AK173_ChromHMM_simplified_7states.bed
AK173_H3K27ac_coverage_SES_subtract.bigWig
AK173_H3K27ac_peaks.narrowPeak.bed
AK173_H3K27ac_W200-G600-FDR0.01-island.bed
AK173_H3K27me3_coverage_SES_subtract.bigWig
AK173_H3K27me3_peaks.narrowPeak.bed
AK173_H3K27me3_W200-G600-FDR0.01-island.bed
AK173_H3K36me3_coverage_SES_subtract.bigWig
AK173_H3K36me3_peaks.narrowPeak.bed
AK173_H3K36me3_W200-G600-FDR0.01-island.bed
AK173_H3K4me1_coverage_SES_subtract.bigWig
AK173_H3K4me1_peaks.narrowPeak.bed
AK173_H3K4me1_W200-G600-FDR0.01-island.bed
AK173_H3K4me3_coverage_SES_subtract.bigWig
AK173_H3K4me3_peaks.narrowPeak.bed
AK173_H3K4me3_W200-G600-FDR0.01-island.bed
AK173_H3K9me3_coverage_SES_subtract.bigWig
AK173_H3K9me3_peaks.narrowPeak.bed
AK173_H3K9me3_W200-G600-FDR0.01-island.bed
AK178_ChromHMM_18states.bed
AK178_ChromHMM_simplified_7states.bed
AK178_H3K27ac_coverage_SES_subtract.bigWig
AK178_H3K27ac_peaks.narrowPeak.bed
AK178_H3K27ac_W200-G600-FDR0.01-island.bed
AK178_H3K27me3_coverage_SES_subtract.bigWig
AK178_H3K27me3_peaks.narrowPeak.bed
AK178_H3K27me3_W200-G600-FDR0.01-island.bed
AK178_H3K36me3_coverage_SES_subtract.bigWig
AK178_H3K36me3_peaks.narrowPeak.bed
AK178_H3K36me3_W200-G600-FDR0.01-island.bed
AK178_H3K4me1_coverage_SES_subtract.bigWig
AK178_H3K4me1_peaks.narrowPeak.bed
AK178_H3K4me1_W200-G600-FDR0.01-island.bed
AK178_H3K4me3_coverage_SES_subtract.bigWig
AK178_H3K4me3_peaks.narrowPeak.bed
AK178_H3K4me3_W200-G600-FDR0.01-island.bed
AK178_H3K9me3_coverage_SES_subtract.bigWig
AK178_H3K9me3_peaks.narrowPeak.bed
AK178_H3K9me3_W200-G600-FDR0.01-island.bed
AK183_ChromHMM_18states.bed

AK183_ChromHMM_simplified_7states.bed
AK183_H3K27ac_coverage_SES_subtract.bigWig
AK183_H3K27ac_peaks.narrowPeak.bed
AK183_H3K27ac_W200-G600-FDR0.01-island.bed
AK183_H3K27me3_coverage_SES_subtract.bigWig
AK183_H3K27me3_peaks.narrowPeak.bed
AK183_H3K27me3_W200-G600-FDR0.01-island.bed
AK183_H3K36me3_coverage_SES_subtract.bigWig
AK183_H3K36me3_peaks.narrowPeak.bed
AK183_H3K36me3_W200-G600-FDR0.01-island.bed
AK183_H3K4me1_coverage_SES_subtract.bigWig
AK183_H3K4me1_peaks.narrowPeak.bed
AK183_H3K4me1_W200-G600-FDR0.01-island.bed
AK183_H3K4me3_coverage_SES_subtract.bigWig
AK183_H3K4me3_peaks.narrowPeak.bed
AK183_H3K4me3_W200-G600-FDR0.01-island.bed
AK183_H3K9me3_coverage_SES_subtract.bigWig
AK183_H3K9me3_peaks.narrowPeak.bed
AK183_H3K9me3_W200-G600-FDR0.01-island.bed
AK199_ChromHMM_18states.bed
AK199_ChromHMM_simplified_7states.bed
AK199_H3K27ac_coverage_SES_subtract.bigWig
AK199_H3K27ac_peaks.narrowPeak.bed
AK199_H3K27ac_W200-G600-FDR0.01-island.bed
AK199_H3K27me3_coverage_SES_subtract.bigWig
AK199_H3K27me3_peaks.narrowPeak.bed
AK199_H3K27me3_W200-G600-FDR0.01-island.bed
AK199_H3K36me3_coverage_SES_subtract.bigWig
AK199_H3K36me3_peaks.narrowPeak.bed
AK199_H3K36me3_W200-G600-FDR0.01-island.bed
AK199_H3K4me1_coverage_SES_subtract.bigWig
AK199_H3K4me1_peaks.narrowPeak.bed
AK199_H3K4me1_W200-G600-FDR0.01-island.bed
AK199_H3K4me3_coverage_SES_subtract.bigWig
AK199_H3K4me3_peaks.narrowPeak.bed
AK199_H3K4me3_W200-G600-FDR0.01-island.bed
AK199_H3K9me3_coverage_SES_subtract.bigWig
AK199_H3K9me3_peaks.narrowPeak.bed
AK199_H3K9me3_W200-G600-FDR0.01-island.bed
AK213_ChromHMM_18states.bed
AK213_ChromHMM_simplified_7states.bed
AK213_H3K27ac_coverage_SES_subtract.bigWig
AK213_H3K27ac_peaks.narrowPeak.bed
AK213_H3K27ac_W200-G600-FDR0.01-island.bed
AK213_H3K27me3_coverage_SES_subtract.bigWig
AK213_H3K27me3_peaks.narrowPeak.bed
AK213_H3K27me3_W200-G600-FDR0.01-island.bed
AK213_H3K36me3_coverage_SES_subtract.bigWig
AK213_H3K36me3_peaks.narrowPeak.bed
AK213_H3K36me3_W200-G600-FDR0.01-island.bed
AK213_H3K4me1_coverage_SES_subtract.bigWig
AK213_H3K4me1_peaks.narrowPeak.bed
AK213_H3K4me1_W200-G600-FDR0.01-island.bed
AK213_H3K4me3_coverage_SES_subtract.bigWig
AK213_H3K4me3_peaks.narrowPeak.bed
AK213_H3K4me3_W200-G600-FDR0.01-island.bed
AK213_H3K9me3_coverage_SES_subtract.bigWig
AK213_H3K9me3_peaks.narrowPeak.bed
AK213_H3K9me3_W200-G600-FDR0.01-island.bed
AK216_ChromHMM_18states.bed
AK216_ChromHMM_simplified_7states.bed
AK216_H3K27ac_coverage_SES_subtract.bigWig
AK216_H3K27ac_peaks.narrowPeak.bed
AK216_H3K27ac_W200-G600-FDR0.01-island.bed
AK216_H3K27me3_coverage_SES_subtract.bigWig
AK216_H3K27me3_peaks.narrowPeak.bed
AK216_H3K27me3_W200-G600-FDR0.01-island.bed
AK216_H3K36me3_coverage_SES_subtract.bigWig

AK216_H3K36me3_peaks.narrowPeak.bed
AK216_H3K36me3_W200-G600-FDR0.01-island.bed
AK216_H3K4me1_coverage_SES_subtract.bigWig
AK216_H3K4me1_peaks.narrowPeak.bed
AK216_H3K4me1_W200-G600-FDR0.01-island.bed
AK216_H3K4me3_coverage_SES_subtract.bigWig
AK216_H3K4me3_peaks.narrowPeak.bed
AK216_H3K4me3_W200-G600-FDR0.01-island.bed
AK216_H3K9me3_coverage_SES_subtract.bigWig
AK216_H3K9me3_peaks.narrowPeak.bed
AK216_H3K9me3_W200-G600-FDR0.01-island.bed
AK231_ChromHMM_18states.bed
AK231_ChromHMM_simplified_7states.bed
AK231_H3K27ac_coverage_SES_subtract.bigWig
AK231_H3K27ac_peaks.narrowPeak.bed
AK231_H3K27ac_W200-G600-FDR0.01-island.bed
AK231_H3K27me3_coverage_SES_subtract.bigWig
AK231_H3K27me3_peaks.narrowPeak.bed
AK231_H3K27me3_W200-G600-FDR0.01-island.bed
AK231_H3K36me3_coverage_SES_subtract.bigWig
AK231_H3K36me3_peaks.narrowPeak.bed
AK231_H3K36me3_W200-G600-FDR0.01-island.bed
AK231_H3K4me1_coverage_SES_subtract.bigWig
AK231_H3K4me1_peaks.narrowPeak.bed
AK231_H3K4me1_W200-G600-FDR0.01-island.bed
AK231_H3K4me3_coverage_SES_subtract.bigWig
AK231_H3K4me3_peaks.narrowPeak.bed
AK231_H3K4me3_W200-G600-FDR0.01-island.bed
AK231_H3K9me3_coverage_SES_subtract.bigWig
AK231_H3K9me3_peaks.narrowPeak.bed
AK231_H3K9me3_W200-G600-FDR0.01-island.bed
GB_H3K27ac_superenhancers_IDH_specific_FDR_0.1_log2FC_1.bed
GB_H3K27ac_superenhancers_matrix_with_target_gene_predictions.csv
GB_H3K27ac_superenhancers_MES_specific_FDR_0.1_log2FC_1.bed
GB_H3K27ac_superenhancers_RTK_I_specific_FDR_0.1_log2FC_1.bed
GB_H3K27ac_superenhancers_RTK_II_specific_FDR_0.1_log2FC_1.bed
GB_H3K27ac_superenhancers.bed
IDH_ChromHMM_18states.bed
IDH_ChromHMM_simplified_7states.bed
IDH_subtype_H3K27ac_superenhancers.bed
MES_ChromHMM_18states.bed
MES_ChromHMM_simplified_7states.bed
MES_subtype_H3K27ac_superenhancers.bed
NBr_ChromHMM_18states.bed
NBr_ChromHMM_simplified_7states.bed
RTK_I_ChromHMM_18states.bed
RTK_I_ChromHMM_simplified_7states.bed
RTK_I_subtype_H3K27ac_superenhancers.bed
RTK_II_ChromHMM_18states.bed
RTK_II_ChromHMM_simplified_7states.bed
RTK_II_subtype_H3K27ac_superenhancers.bed
LN229_shControl_ChromHMM_18states.bed
LN229_shControl_ChromHMM_simplified_7states.bed
LN229_shControl_H3_coverage_SES_subtract.bigWig
LN229_shControl_H3K27ac_coverage_SES_subtract.bigWig
LN229_shControl_H3K27ac_peaks.narrowPeak.bed
LN229_shControl_H3K27ac_W200-G600-FDR0.01-island.bed
LN229_shControl_H3K27me3_coverage_SES_subtract.bigWig
LN229_shControl_H3K27me3_peaks.narrowPeak.bed
LN229_shControl_H3K27me3_W200-G600-FDR0.01-island.bed
LN229_shControl_H3K36me3_coverage_SES_subtract.bigWig
LN229_shControl_H3K36me3_peaks.narrowPeak.bed
LN229_shControl_H3K36me3_W200-G600-FDR0.01-island.bed
LN229_shControl_H3K4me1_coverage_SES_subtract.bigWig
LN229_shControl_H3K4me1_peaks.narrowPeak.bed
LN229_shControl_H3K4me1_W200-G600-FDR0.01-island.bed
LN229_shControl_H3K4me3_coverage_SES_subtract.bigWig
LN229_shControl_H3K4me3_peaks.narrowPeak.bed

LN229_shControl_H3K4me3_W200-G600-FDR0.01-island.bed
LN229_shControl_H3K9me3_coverage_SES_subtract.bigWig
LN229_shControl_H3K9me3_peaks.narrowPeak.bed
LN229_shControl_H3K9me3_W200-G600-FDR0.01-island.bed
LN229_shSOX10_ChromHMM_18states.bed
LN229_shSOX10_ChromHMM_simplified_7states.bed
LN229_shSOX10_H3_coverage_SES_subtract.bigWig
LN229_shSOX10_H3K27ac_coverage_SES_subtract.bigWig
LN229_shSOX10_H3K27ac_peaks.narrowPeak.bed
LN229_shSOX10_H3K27ac_W200-G600-FDR0.01-island.bed
LN229_shSOX10_H3K27me3_coverage_SES_subtract.bigWig
LN229_shSOX10_H3K27me3_peaks.narrowPeak.bed
LN229_shSOX10_H3K27me3_W200-G600-FDR0.01-island.bed
LN229_shSOX10_H3K36me3_coverage_SES_subtract.bigWig
LN229_shSOX10_H3K36me3_peaks.narrowPeak.bed
LN229_shSOX10_H3K36me3_W200-G600-FDR0.01-island.bed
LN229_shSOX10_H3K4me1_coverage_SES_subtract.bigWig
LN229_shSOX10_H3K4me1_peaks.narrowPeak.bed
LN229_shSOX10_H3K4me1_W200-G600-FDR0.01-island.bed
LN229_shSOX10_H3K4me3_coverage_SES_subtract.bigWig
LN229_shSOX10_H3K4me3_peaks.narrowPeak.bed
LN229_shSOX10_H3K4me3_W200-G600-FDR0.01-island.bed
LN229_shSOX10_H3K9me3_coverage_SES_subtract.bigWig
LN229_shSOX10_H3K9me3_peaks.narrowPeak.bed
LN229_shControl_BRD4_coverage_SES_subtract.bw
LN229_shControl_BRD4_peaks_narrowPeaks.bed
LN229_shControl_SOX10_coverage_SES_subtract.bw
LN229_shControl_SOX10_peaks_narrowPeaks.bed
LN229_shSOX10_BRD4_coverage_SES_subtract.bw
LN229_shSOX10_BRD4_peaks_narrowPeaks.bed
ZH487_shControl_BRD4_coverage_SES_subtract.bw
ZH487_shControl_BRD4_peaks_narrowPeaks.bed
ZH487_shControl_RNAseq_coverage.bigWig
ZH487_shControl_SOX10_coverage_SES_subtract.bw
ZH487_shControl_SOX10_peaks_narrowPeaks.bed
ZH487_shSOX10_BRD4_coverage_SES_subtract.bw
ZH487_shSOX10_BRD4_peaks_narrowPeaks.bed
ZH487_shSOX10_RNAseq_coverage.bigWig

Genome browser session
(e.g. UCSC)

No longer applicable.

Methodology

Replicates

A single replicate was performed for tumors and cell lines

Sequencing depth

All ChIP-seq sequencing was done as single-end sequencing with read length of 50bp.

Sample Condition Mark Uniquely mapped reads MACS2 narrow peaks (FDR=1%) MACS2 broad peaks (FDR=1%) MCS2 FRiP SICER FRiP NSC RSC
LN229 Ctrl H3 24512489 1.026785 1.73064
LN229 shSOX10 H3 21811954 1.025389 1.348918
ZH487 Ctrl H3 20613046 1.028871 1.404383
ZH487 shSOX10 H3 23258864 1.02604 1.604519

LN229 Ctrl H3K27ac 25430164 9272 8560 0.0150215 0.0892369 1.033626 2.248043
LN229 shSOX10 H3K27ac 23022472 21095 17743 0.0410475 0.14945 1.042754 1.991535
ZH487 Ctrl H3K27ac 17715461 4725 5860 0.00725722 0.0681532 1.04017 2.111029
ZH487 shSOX10 H3K27ac 20787805 698 1105 0.000828851 0.0379295 1.03143 2.165912

LN229 Ctrl H3K27me3 22816947 8019 6493 0.0144241 0.105062 1.034034 2.204209
LN229 shSOX10 H3K27me3 21192391 14961 8149 0.0338666 0.156842 1.038613 2.220496
ZH487 Ctrl H3K27me3 21346470 653 1663 0.000719135 0.13586 1.037528 1.74075
ZH487 shSOX10 H3K27me3 22259161 26830 19990 0.046341 0.266631 1.046331 2.322192

LN229 Ctrl H3K36me3 24206405 86826 40431 0.144512 0.392729 1.055404 2.248104
LN229 shSOX10 H3K36me3 24312662 92041 40935 0.181047 0.448887 1.062624 2.190289
ZH487 Ctrl H3K36me3 21845653 43863 38850 0.0593123 0.375396 1.055985 2.097624
ZH487 shSOX10 H3K36me3 22971151 44653 37864 0.0572665 0.324928 1.048436 2.140586

LN229 Ctrl H3K4me1 24958627 15978 19713 0.0211765 0.254768 1.043374 2.756624
LN229 shSOX10 H3K4me1 21793848 54237 42658 0.0881653 0.338592 1.053911 2.632628
ZH487 Ctrl H3K4me1 21062645 941 3606 0.00100885 0.195677 1.045271 2.547238
ZH487 shSOX10 H3K4me1 22429057 6585 14849 0.00664709 0.227243 1.042233 2.779748

LN229 Ctrl H3K4me3 22857403 23614 14550 0.118403 0.168187 1.085103 2.826811
LN229 shSOX10 H3K4me3 23287389 22172 15527 0.19743 0.244145 1.182384 2.400035
ZH487 Ctrl H3K4me3 21740562 19293 13887 0.1466 0.196481 1.139643 2.5651
ZH487 shSOX10 H3K4me3 32327920 20536 13579 0.119495 0.184294 1.094075 2.74555

LN229 Ctrl H3K9me3 23381932 8470 22802 0.0121544 0.345482 1.042052 1.182686
LN229 shSOX10 H3K9me3 21221052 14946 32451 0.0201362 0.390599 1.046882 1.107382
ZH487 Ctrl H3K9me3 20192760 167 360 0.000663852 0.145437 1.043036 1.029732
ZH487 shSOX10 H3K9me3 19518520 67 185 0.000354023 0.118737 1.036588 1.015953

Sample Mark Uniquely Aligned Reads MACS2 narrow peaks MACS2 broad peaks SICER peaks Macs2_FRiP SICER_FRiP NSC RSC
AK066 H3K27ac 12533680 38000 28090 40909 0.407 0.531 1.347 1.571
AK071 H3K27ac 31566410 44288 35078 46710 0.482 0.599 1.665 1.619
AK076 H3K27ac 20428917 48594 37982 45984 0.556 0.669 1.578 1.853
AK089 H3K27ac 27095860 40578 32594 47952 0.558 0.680 1.816 1.687
AK091 H3K27ac 24359133 40493 31357 49861 0.436 0.568 1.521 1.485
AK100 H3K27ac 26707287 40542 32798 46235 0.682 0.768 1.955 1.885
AK124 H3K27ac 27764449 39364 31797 45320 0.536 0.663 1.797 1.787
AK139 H3K27ac 28265580 19765 15999 39586 0.147 0.326 1.252 1.535
AK142 H3K27ac 31340856 36567 29042 38196 0.579 0.680 1.574 1.617
AK149 H3K27ac 7614120 48120 37639 35223 0.405 0.482 1.543 1.527
AK153 H3K27ac 33683525 48082 34377 53360 0.520 0.674 1.474 1.439
AK156 H3K27ac 32184220 35434 27269 39819 0.498 0.640 1.849 1.456
AK158 H3K27ac 37042079 41262 32147 45102 0.593 0.731 1.875 1.624
AK173 H3K27ac 24716555 45564 33270 51630 0.452 0.616 1.412 1.702
AK178 H3K27ac 35831618 38317 31033 48030 0.616 0.738 1.820 1.628
AK183 H3K27ac 35115386 47184 36391 57016 0.440 0.579 1.699 1.638
AK199 H3K27ac 29759682 40866 32986 46545 0.539 0.654 1.836 1.736
AK213 H3K27ac 23868584 39008 29831 45767 0.417 0.562 1.544 1.683
AK216 H3K27ac 30120840 38207 28945 44340 0.431 0.579 1.438 1.494
AK231 H3K27ac 33242415 53554 39486 52080 0.527 0.684 1.675 1.716

AK066 H3K27me3 29575965 3005 2270 50625 0.021 0.290 1.039 1.042
AK071 H3K27me3 24947338 295 503 39300 0.004 0.189 1.043 1.162
AK076 H3K27me3 41336476 2922 2900 45002 0.019 0.269 1.038 0.963
AK089 H3K27me3 45939938 10297 8229 44819 0.067 0.379 1.039 0.826
AK091 H3K27me3 37913736 1530 2028 43735 0.010 0.221 1.029 0.700
AK100 H3K27me3 23923310 8835 6535 39517 0.083 0.405 1.061 0.974
AK124 H3K27me3 19971600 8874 5900 39605 0.056 0.276 1.039 0.854
AK139 H3K27me3 25742116 5916 4462 27214 0.035 0.209 1.032 0.819
AK142 H3K27me3 24714525 10529 8062 36044 0.067 0.340 1.045 0.711
AK149 H3K27me3 19003438 2805 2970 43359 0.017 0.211 1.026 0.699
AK153 H3K27me3 18114281 2784 2806 41373 0.018 0.199 1.034 0.841
AK156 H3K27me3 18132032 9787 7396 45217 0.068 0.302 1.039 0.999
AK158 H3K27me3 19007955 14003 9376 45075 0.122 0.416 1.058 0.973
AK173 H3K27me3 60208456 15722 10412 46940 0.096 0.412 1.033 0.833
AK178 H3K27me3 42110480 3240 2961 46686 0.028 0.338 1.043 0.926
AK183 H3K27me3 41936266 12769 9433 43628 0.079 0.326 1.038 0.858
AK199 H3K27me3 19389899 16227 11294 48983 0.107 0.391 1.053 0.857
AK213 H3K27me3 23745292 2908 2994 40435 0.019 0.317 1.034 0.809
AK216 H3K27me3 23792298 3767 3998 42953 0.024 0.319 1.030 0.839
AK231 H3K27me3 18267008 4674 3842 31666 0.030 0.276 1.046 0.995

AK066 H3K4me1 12917037 19154 16916 53699 0.128 0.407 1.062 1.078
AK071 H3K4me1 30165521 35066 26172 67688 0.214 0.552 1.080 1.098
AK076 H3K4me1 11655339 40382 33790 61828 0.234 0.483 1.078 1.077
AK089 H3K4me1 28438485 34638 26382 56422 0.234 0.554 1.072 1.280
AK091 H3K4me1 19211822 23903 20696 69101 0.137 0.449 1.054 0.977
AK100 H3K4me1 25640515 41608 28623 54073 0.330 0.616 1.120 1.165
AK124 H3K4me1 28495618 46391 28027 58715 0.371 0.661 1.087 1.158
AK139 H3K4me1 20703745 20596 18269 70086 0.119 0.466 1.047 0.962

AK142 H3K4me1 31736727 54205 33209 58503 0.390 0.680 1.111 1.082
AK149 H3K4me1 5350305 29496 27701 42727 0.178 0.338 1.062 0.966
AK153 H3K4me1 26863770 34664 24864 72563 0.219 0.592 1.057 0.943
AK156 H3K4me1 12989333 44222 30258 54847 0.339 0.596 1.094 1.109
AK158 H3K4me1 17803207 39563 25868 45615 0.342 0.657 1.089 1.111
AK173 H3K4me1 16853351 38882 26852 48870 0.242 0.552 1.067 1.080
AK178 H3K4me1 22748540 36741 25084 57037 0.308 0.640 1.091 1.108
AK183 H3K4me1 35025985 26197 18458 60484 0.163 0.585 1.063 1.048
AK199 H3K4me1 29202519 35992 25668 64989 0.263 0.581 1.100 1.107
AK213 H3K4me1 18988518 25462 20507 63268 0.149 0.461 1.054 1.058
AK216 H3K4me1 22456040 32130 26676 66185 0.182 0.500 1.067 1.097
AK231 H3K4me1 11348604 46354 33827 59121 0.281 0.539 1.064 1.053

AK066 H3K4me3 37403467 20747 16190 25035 0.448 0.544 1.866 1.297
AK071 H3K4me3 24802407 24871 19966 26334 0.521 0.570 2.384 1.551
AK076 H3K4me3 48896166 25816 22270 28818 0.623 0.674 2.405 1.482
AK089 H3K4me3 64737234 26297 20237 29402 0.618 0.701 1.864 1.297
AK091 H3K4me3 49980796 27174 20669 28146 0.476 0.564 1.635 1.248
AK100 H3K4me3 36028796 27225 19461 28123 0.681 0.745 2.052 1.384
AK124 H3K4me3 29510615 27255 20575 25676 0.666 0.743 2.238 1.439
AK139 H3K4me3 36232299 27841 20025 29778 0.496 0.591 1.681 1.293
AK142 H3K4me3 38017190 25732 17545 23331 0.666 0.714 2.015 1.289
AK149 H3K4me3 25093560 24424 18497 24396 0.468 0.543 1.815 1.297
AK153 H3K4me3 30277101 27391 19235 28415 0.548 0.635 1.746 1.286
AK156 H3K4me3 23072061 24076 18983 25715 0.619 0.689 2.129 1.420
AK158 H3K4me3 26830381 23579 18089 24054 0.628 0.691 2.310 1.441
AK173 H3K4me3 57509726 27065 19449 23716 0.588 0.664 2.036 1.465
AK178 H3K4me3 40133654 26559 19759 30244 0.632 0.718 1.949 1.470
AK183 H3K4me3 52077962 25769 19032 24122 0.587 0.661 2.010 1.397
AK199 H3K4me3 35329114 24508 18011 24892 0.635 0.694 2.201 1.315
AK213 H3K4me3 38693955 26266 20038 27222 0.521 0.610 1.862 1.339
AK216 H3K4me3 26154448 25268 19618 25837 0.568 0.645 1.971 1.262
AK231 H3K4me3 28285601 26217 20555 25673 0.679 0.744 2.169 1.473

AK066 H3K9me3 16136468 1132 1059 29023 0.017 0.178 1.033 0.349
AK071 H3K9me3 17105556 3488 3388 32311 0.047 0.226 1.046 0.317
AK076 H3K9me3 38482722 3436 3740 34599 0.038 0.264 1.050 0.469
AK089 H3K9me3 43903910 2402 2392 49004 0.031 0.255 1.044 0.428
AK091 H3K9me3 51096666 1234 953 33583 0.021 0.191 1.036 0.431
AK100 H3K9me3 19031385 5493 4851 54573 0.060 0.302 1.068 0.461
AK124 H3K9me3 6316418 5554 7725 24817 0.037 0.143 1.059 0.307
AK139 H3K9me3 24241086 469 420 36194 0.008 0.163 1.042 0.651
AK142 H3K9me3 15535198 9928 9999 46935 0.068 0.336 1.072 0.545
AK149 H3K9me3 21808504 432 496 23428 0.007 0.096 1.031 0.465
AK153 H3K9me3 6732220 8860 10452 26575 0.071 0.199 1.058 0.194
AK156 H3K9me3 24139665 2349 2331 51177 0.023 0.322 1.039 0.484
AK158 H3K9me3 27618268 3305 3129 66787 0.035 0.367 1.054 0.574
AK173 H3K9me3 37066656 2029 1976 46989 0.023 0.204 1.044 0.438
AK178 H3K9me3 37453998 3220 3089 59019 0.038 0.296 1.067 0.526
AK183 H3K9me3 67181242 2026 1839 51428 0.024 0.285 1.037 0.501
AK199 H3K9me3 22437174 8679 7924 55088 0.060 0.362 1.056 0.549
AK213 H3K9me3 31005153 929 910 58555 0.012 0.247 1.042 0.660
AK216 H3K9me3 19994983 1790 1471 41876 0.029 0.192 1.041 0.362
AK231 H3K9me3 28738910 2482 2587 64086 0.023 0.276 1.050 0.645

AK066 H3K36me3 NA NA NA NA NA NA NA NA
AK071 H3K36me3 22574974 34338 21453 42190 0.204 0.515 1.071 0.984
AK076 H3K36me3 51164902 53209 28760 39039 0.443 0.679 1.114 1.072
AK089 H3K36me3 32431850 38116 22217 33571 0.260 0.536 1.053 0.827
AK091 H3K36me3 51281494 28312 18670 42173 0.172 0.491 1.058 1.013
AK100 H3K36me3 24934005 38830 20601 35648 0.335 0.607 1.092 0.916
AK124 H3K36me3 22002376 38282 20924 38160 0.266 0.551 1.077 0.945
AK139 H3K36me3 24782944 30642 20167 44438 0.201 0.476 1.074 1.139
AK142 H3K36me3 27813661 48539 25045 32969 0.349 0.628 1.092 1.022
AK149 H3K36me3 30892255 29682 18855 43832 0.174 0.501 1.054 0.909
AK153 H3K36me3 20590823 39688 24153 45462 0.246 0.536 1.072 1.012
AK156 H3K36me3 25193562 27329 16780 39023 0.186 0.537 1.060 0.988
AK158 H3K36me3 26400055 37337 19695 33668 0.319 0.650 1.072 0.900

	AK173 H3K36me3 54683540 41922 23202 36145 0.282 0.582 1.059 0.929<br>AK178 H3K36me3 45599734 31728 17886 39160 0.273 0.599 1.078 0.995<br>AK183 H3K36me3 51098620 29280 19671 35698 0.167 0.524 1.050 0.837<br>AK199 H3K36me3 19575484 36097 21908 44137 0.235 0.501 1.062 0.829<br>AK213 H3K36me3 28469409 20325 13254 47297 0.119 0.413 1.048 0.937<br>AK216 H3K36me3 29512243 41459 23775 52168 0.269 0.552 1.089 1.189<br>AK231 H3K36me3 14150415 57696 31117 36494 0.397 0.616 1.090 0.954
Antibodies	For tumour ChIPseq experiments; antibodies are from Active Motif unless otherwise specified: H3K27Ac (AM#39133, lot 5), H3K4me1 (AM#38297, lot 1), H3K4me3 (AM#39159, lot 2), H3K9me3 (AM#39161, lot 3), H3K27me3 (#07-449, lot 2382150, Millipore) and H3K36me3 (AM#61101, lot 7).<br><br>For cell line experiments: H3, ab1791; H3K4me1, ab8895; H3K4me3, ab8580; H3K9me3, ab8898; H3K27ac, ab4729; H3K36me3, ab9050; H3K27me3, ab6002; all Abcam. In addition, antibodies of BRD4 (Bethyl Lab., Cat#A301-985A100) and SOX10 (Abcam, Cat#ab155279) were also used for TF-ChIPseq.
Peak calling parameters	MACS2 narrow peaks: macs2 callpeak -t {input.ip} -c {input.control} -g hs --outdir {params.outdir} -n {params.name} -q 0.01 --SPMR<br>MACS2 broad peaks : macs2 callpeak -t {input.ip} -c {input.control} -g hs --outdir {params.outdir} -n {params.name} --broad --broad-cutoff 0.01<br>SICER peaks: SICER.sh {params.outdir} $IPBED $CONTROLBED {params.outdir} hg19 1 200 150 0.7 600 0.01
Data quality	Methods for QC determination in ChIP-seq experiments were comparable to those used by the ENCODE consortium<br>1. fraction of reads in peaks (FRiP) determined both using MACS2 narrow peaks and SICER peaks<br>2. normalized and relative strand correlation (NSC / RSC)<br>3. Number of peaks / aligned reads<br>4. visual inspection of bigwig tracks<br><br>See table above for all QC parameters
Software	The ChIP-seq analysis pipeline was implemented in Snakemake v 3.13.3; it is available under https://github.com/dkfz-b060

Magnetic resonance imaging

Experimental design

Design type	MRI was performed in resting state under anesthesia.
Design specifications	Not applicable
Behavioral performance measures	Not applicable

Acquisition

Imaging type(s)	Structural, perfusion
Field strength	1 Tesla
Sequence & imaging parameters	*Specify the pulse sequence type (gradient echo, spin echo, etc.), imaging type (EPI, spiral, etc.), field of view, matrix size, slice thickness, orientation and TE/TR/flip angle.*
Area of acquisition	Whole brain

Diffusion MRI ☐ Used ☒ Not used

Preprocessing

Preprocessing software	*Provide detail on software version and revision number and on specific parameters (model/functions, brain extraction, segmentation, smoothing kernel size, etc.).*
Normalization	*If data were normalized/standardized, describe the approach(es): specify linear or non-linear and define image types used for transformation OR indicate that data were not normalized and explain rationale for lack of normalization.*
Normalization template	*Describe the template used for normalization/transformation, specifying subject space or group standardized space (e.g. original Talairach, MNI305, ICBM152) OR indicate that the data were not normalized.*
Noise and artifact removal	*Describe your procedure(s) for artifact and structured noise removal, specifying motion parameters, tissue signals and physiological signals (heart rate, respiration).*
Volume censoring	*Define your software and/or method and criteria for volume censoring, and state the extent of such censoring.*

Statistical modeling & inference

Model type and settings

Specify type (mass univariate, multivariate, RSA, predictive, etc.) and describe essential details of the model at the first and second levels (e.g. fixed, random or mixed effects; drift or auto-correlation).

Effect(s) tested

Define precise effect in terms of the task or stimulus conditions instead of psychological concepts and indicate whether ANOVA or factorial designs were used.

Specify type of analysis: ☐ Whole brain ☐ ROI-based ☐ Both

Statistic type for inference
(See Eklund et al. 2016)

Specify voxel-wise or cluster-wise and report all relevant parameters for cluster-wise methods.

Correction

Describe the type of correction and how it is obtained for multiple comparisons (e.g. FWE, FDR, permutation or Monte Carlo).

Models & analysis

n/a	Involved in the study
☐	☐ Functional and/or effective connectivity
☐	☐ Graph analysis
☐	☐ Multivariate modeling or predictive analysis

Functional and/or effective connectivity

Report the measures of dependence used and the model details (e.g. Pearson correlation, partial correlation, mutual information).

Graph analysis

Report the dependent variable and connectivity measure, specifying weighted graph or binarized graph, subject- or group-level, and the global and/or node summaries used (e.g. clustering coefficient, efficiency, etc.).

Multivariate modeling and predictive analysis

Specify independent variables, features extraction and dimension reduction, model, training and evaluation metrics.
